# Unveiling the role of the upper respiratory tract microbiome in susceptibility and severity to COVID-19

**DOI:** 10.3389/fcimb.2025.1531084

**Published:** 2025-05-13

**Authors:** Otávio von Ameln Lovison, Fabiana Caroline Zempulski Volpato, Lorenzo Gómez Weber, Afonso Luis Barth, Adriana Simon Coitinho, Andreza Francisco Martins

**Affiliations:** ^1^ Laboratório de Pesquisa em Resistência Bacteriana (LABRESIS), Hospital de Clínicas de Porto Alegre (HCPA), Centro de Pesquisa Experimental, Porto Alegre, Brazil; ^2^ Laboratório de Microbiologia e Saúde Única do Instituto de Ciências Básicas da Saúde (ICBS) da Universidade Federal do Rio Grande do Sul, Porto Alegre, Brazil; ^3^ Núcleo de Bioinformática (Bioinformatics Core), Hospital de Clínicas de Porto Alegre (HCPA), Porto Alegre, Brazil; ^4^ Laboratório de Neuroimunologia do Instituto de Ciências Básicas da Saúde (ICBS) da Universidade Federal do Rio Grande do Sul, Porto Alegre, Brazil

**Keywords:** SARS-CoV-2, COVID-19, microbiome, upper respiratory tract, immune modulation

## Abstract

It is argued that commensal bacteria in the upper respiratory tract (URT) protect against pathogen colonization and infection, including respiratory viruses. Given that the microbiome can mediate immune modulation, a link between the URT microbiome (URTM) and COVID-19 susceptibility and severity is expected. This 16S metagenomics cross-sectional study assessed URTM composition, metabolic prediction, and association with laboratory biomarkers in non-COVID-19 pneumonia (NO-CoV), moderate (M-CoV), severe (S-CoV) COVID-19 patients, as well as COVID-19-negative, asymptomatic (NC) patients. The S-CoV group exhibited reduced URTM diversity, primarily due to a decreased abundance of eubiotic taxa. Some of these taxa (e.g., *Haemophilus* sp., *Neisseria* sp.) were also associated with inflammatory biomarkers. Multiple metabolic pathways (e.g., short-chain fatty acids, vitamin B12) linked to immune response, antiviral activity, and host susceptibility showed decreased abundance in S-CoV. These pathways could suggest potential alternatives for the therapeutic arsenal against COVID-19, providing reassurance about the progress in understanding and treating this disease.

## 1 Introduction

The severe acute respiratory syndrome coronavirus 2 (SARS-CoV-2), the causative agent of coronavirus disease 19 (COVID-19), continues to pose a global threat due to its ability to evade the immune system through genetic mutations. Despite the pandemic slowdown, immunized individuals remain susceptible to SARS-CoV-2, which is believed to keep circulating for many years (Kissler et al., 2020; Rossman et al., 2021; Barreiro and San Román, 2022).

In COVID-19, both immune suppression and runaway inflammation have been observed and can result in more severe disease, while protective immunity consists of the induction of both humoral and cell-mediated responses (Merenstein et al., 2022). The SARS-CoV-2 inhibits Interferons I and III (IFN-I and IFN-III) and pro-inflammatory responses, promoting high viral replication in the respiratory tract (Wei et al., 2020). Paradoxically, this immune suppression leads to increased circulating cytokines in COVID-19-associated pneumonia. Severe lung damage from viral replication causes epithelial barrier breakdown, triggering a ‘cytokine storm’ with both pro- and anti-inflammatory actions (Tisoncik et al., 2012; Chen et al., 2020; Hall et al., 2022), which impair lung function promoting immunoparalysis, resulting in irreversible damage (Wong et al., 2019).

In the upper respiratory tract (URT), which comprises the nasal cavity, nasopharynx, and oropharynx, high bacterial densities are found (10^3–^10^6^ U^−1^), while in the lung, which belongs to the lower respiratory tract (LRT), bacterial densities decrease (~10^2^ U^−1^). Genera characteristic of a healthy URT include *Staphylococcus* sp., *Propionibacterium* sp., *Leptotrichia* sp., *Rothia* sp., *Dolosigranulum* sp., *Haemophilus* sp., *Moraxella* sp., *Veillonella* sp.*, Prevotella* sp.*, Streptococcus* sp. and *Corynebacterium* sp (Man et al., 2017; Wypych et al., 2019). In eubiosis, these microorganisms act as ‘guardians of respiratory health’ (Wong et al., 2019; Wypych et al., 2019), avoiding colonization of the URT by pathogens, including respiratory viruses, suggesting a potential role in resisting SARS-CoV-2 infection (Bogaert et al., 2004; Wang et al., 2022).

The interactions between the microbiome and its host extend to crucial functions such as carbohydrate and protein decomposition, nutrient absorption, vitamin biosynthesis, and modulation of the immune system (Rooks and Garrett, 2016; Zheng et al., 2020). Metabolites and components from these interactions can modulate immune cells through various mechanisms, including signaling of mucosal-associated T cells (MAIT) (Smith et al., 2013; Godfrey et al., 2019; Legoux et al., 2020;, IFN-I (Flerlage et al., 2021), and dendritic cells (Schaupp et al., 2020) beyond the anti-inflammatory activity of Short-Chain Fatty Acids (SCFA) (Fukuda et al., 2011) and regulation of immunoglobulin expression (Corbett et al., 2014; Kim et al., 2016; Dalile et al., 2019; Parrot et al., 2020; Wang et al., 2022).

Recent investigations have revealed substantial dysbiosis in both intestinal and respiratory microbiomes during COVID-19, and their specific compositions are correlated with the severity of the disease and its outcomes (Ferreira et al., 2020; Lloréns-Rico et al., 2021). Additionally, SARS-CoV-2 infection has been found to heighten the susceptibility of patients to secondary pathogens, thereby playing a significant role in exacerbating morbidity and mortality associated with COVID-19 (Cox et al., 2020; Yeoh et al., 2021). Besides, the microbiome has been identified as a modulator of immune responses and diseases in the airways, increasing interest in the interaction between COVID-19 and this microbiome (Merenstein et al., 2022). This study aimed to delve into these interactions at a functional level, identifying taxa and pathways in the URT associated with COVID-19 severity.

## 2 Methods

### 2.1 Study design

This study employed a cross-sectional design focused on analyzing the upper respiratory tract microbiome in the context of COVID-19. 16S rRNA amplicon sequencing was used to assess composition and predict functionality.

A total of 88 samples of nasal and oropharyngeal combined swabs were provided by the Hospital de Clinicas de Porto Alegre (HCPA) biobank. Swabs were immersed in sterile saline solution for routine SARS-CoV-2 screening using rt-qPCR (National Center for Immunization and Respiratory Diseases (U.S.). Monteiro et al., 2023) for diagnostic purposes and stored at -80°C. The experimental groups were categorized based on the World Health Organization severity level classification (World Health Organization, 2020):

Group 1 (M-COV, n = 22): Moderate COVID-19 - Patients with signs and symptoms of pneumonia, respiratory distress syndrome (ARDS), SpO2 ≥ 90%, and a positive rt-qPCR test for SARS-CoV-2.
Group 2 (NO-COV, n = 22): Non-COVID pneumonia - Patients with signs and symptoms of pneumonia, ARDS, SpO2 ≥ 90%, and two negative rt-qPCR test for SARS-CoV-2.
Group 3 (S-COV, n = 22): Severe COVID-19 - Patients with severe signs and symptoms of pneumonia, ARDS, breathing rate > 30/min., SpO2 < 90%, and a positive rt-qPCR test for SARS-CoV-2.
Group 4 (NC, n = 22): Asymptomatic individuals - Highly exposed to SARS-CoV-2, submitted to routine screening with negative results.

In addition to the COVID-19 severity classification, inclusion criteria comprised patients who underwent rt-qPCR for SARS-CoV-2 screening and laboratory tests for biomarkers (except for the NC group), namely: alanine aminotransferase (ALT), aspartate aminotransferase (AST), c-reactive protein (CRP), creatine phosphokinase (CPK), d-dimer (DDI), hemogram, lactate dehydrogenase (LDH), serum creatinine (CRE) and urea. Demographic characteristics and clinical data were obtained from electronic medical records through a query requested by the Center of Data Science of the HCPA. The study was conducted following the Declaration of Helsinki, and the protocol was approved by the Ethics Committee of HCPA (IRB 00000921, CAAE 38972220.3.0000.5327), ensuring compliance with ethical standards and guidelines for human research. The COVID-19 vaccination was unavailable in Brazil during the study period (between July 2020 and February 2021).

### 2.2 Microbiome data generation

#### 2.2.1 DNA extraction, PCR, and sequencing

The DNA extraction was performed with the DNeasy^®^ Blood and Tissue extraction kit (QIAGEN^®^, Canada) following the manufacturer’s recommendations with minor adaptations. The adaptation consists of performing a bead beating procedure with silica/zirconia beads in a MP Biochemicals FastPrep24 Sample Preparation (RRID: SCR_018599) using a 30s protocol at 6.0 m/s repeated three times, after the incubation with proteinase K. The total nucleic acids were eluted in 60 μL of TE buffer (Tris-EDTA pH 8,0) and submitted to amplification of the v3v4 hypervariable region of 16S rRNA using the 16S Metagenomic Sequencing Library Preparation Illumina^®^ (Illumina, USA, 2013; Illumina, 2013). A negative control (molecular grade water) was included for quality assurance. The libraries were sequenced on an Illumina MiSeq System (RRID: SCR_016379) platform using a v2 Reagent Kit (2x250bp; average coverage ~200.000 reads/sample) (Illumina, Inc.). Extreme care was taken to mitigate the possibility of environmental contamination during the entire sample processing (Kirstahler et al., 2018).

#### 2.2.2 Bioinformatics analyses

The bioinformatics analyses were performed with the open-source software R Project for Statistical Computing (RRID: SCR_001905) v. 4.3.2 (Eye Holes) (R Core Team, 2021), the development interface RStudio (RRID: SCR_000432) v. 2022.12.0-353 (RStudio Team, 2020), and packages of the project Bioconductor (RRID: SCR_006442) v. 3.16 (Huber et al., 2015).

Raw reads were processed according to a previous report (Callahan et al., 2016b) using the DADA2 (RRID: SCR_023519) algorithm (Callahan et al., 2016a). Briefly, reads were quality-checked, trimmed, filtered, and truncated to position 240. Paired-end joining, determination of amplicon sequence variants (ASV), and removal of chimeric sequences were performed, followed by the taxonomic assignment using the expanded Human Oral Microbiome Database (RRID: SCR_025964) (eHOMD 16S rRNA RefSeq Version 15.22) (Escapa et al., 2020). The ASV sequences, counts, taxonomy tables, and sample metadata were merged into a phyloseq (RRID: SCR_013080) object (McMurdie and Holmes, 2013) to proceed with the analyses. All the detailed parameters are presented in the Data Sheet 2.

Taxonomic agglomeration was performed to identify the dominant taxa and their relative abundance at phyla, genera, and species levels. The Compositional Data Analysis approach (CODA) (Aitchison, 1982) was adopted to evaluate the microbiome composition using the Centered Log-Ratio (CLR) for log-ratio transformation (Pawlowsky-Glahn et al., 2015; Greenacre et al., 2021; Bars-Cortina, 2022).

The metabolic prediction was performed with the PICRUSt2 (RRID: SCR_022647) pipeline (Douglas et al., 2020). The functional prediction and the pathway inference were performed with a modified version of the MinPath (Minimal set of Pathways) (Ye and Doak, 2009) tool using the MetaCyc (RRID: SCR_007778) database (Caspi et al., 2016). The pathway annotation was performed with the ggpicrust2 (RRID: SCR_025965) package (Yang et al., 2023).

#### 2.2.3 Statistical analysis

α-diversity was performed using the Shannon index followed by the Wilcoxon Rank-sum test (Haegeman et al., 2013). β-diversity was conducted by the Principal Component Analysis (PCA) using Aitchison distance (Aitchison, 1982; Calle, 2019). The statistical significance and the proportion of explained variance were assessed by Permutation Multivariate Analysis of Variance (PERMANOVA) (Anderson, 2017) for experimental groups and each confounding factor (age, sex, and batch).

The taxonomic differential abundance analysis was performed using the Analysis of Compositions of Microbiomes with Bias Correction 2 (ANCOM-BC2 (RRID: SCR_024901)) algorithm (Lin and Peddada, 2020) for the global test (NC as the reference group) and multiple pairwise comparisons at the genus level. The pathway’s differential abundance was evaluated using the LinDA (RRID: SCR_025966) (linear models for differential abundance analysis of microbiome compositional data) (Zhou et al., 2022) algorithm.

We accounted for multiple tests using the Benjamini-Hochberg (BH) method (Benjamini and Hochberg, 1995) for false discovery rate (FDR) and a mixed directional false discovery rate (mdFDR) control using a family-wise error controlling procedure by Holm method (Holm, 1979), with an alpha of 0.05.

We performed a pairwise log-ratio exploratory analysis with the coda4microbiome (Calle et al., 2023) package to evaluate the relationships among taxa and clinical variables. Spearman’s correlation coefficient was used to measure the association between the variables and the predictions of a generalized linear model (GLM). All the detailed parameters used for statistical analysis are presented in Data Sheet 3.

#### 2.2.4 Code availability

The PICRUSt2 (Douglas et al., 2020) is available at https://github.com/picrust/picrust2 Custom scripts and data generated for this paper can be accessible from “Lovison_et_al_2024_COVID19_URT_microbiome”, GitHub (https://github.com/otaviolovison/Lovison_et_al_2024_COVID19_URT_microbiome).

## 3 Results

Among the 88 selected samples, nine were excluded due to the impossibility of extracting high-quality DNA or unsatisfactory sequencing results. The samples and their respective experimental groups that remained for the analysis were M-COV, n = 22; NO-COV, n = 19; S-COV, n = 20; and NC, n = 18. Data Sheet 1 presents a flowchart representing experimental design and the bioinformatics workflow.

The quality check as well as the error rates analysis from the preprocessing and the complete track of the reads are shown in Supplementary Material (**Data Sheet 2**, **Table 1 - Supplementary Data 1**). After the filtering step ( Data Sheet 3- 0.1 Data preparation), 11 phyla, 200 genera, 364 species, and 50,347 ASVs were identified. The complete description, representative proportions, and overall relative abundance of these taxa are presented in Supplementary Material (**Table 1 - Supplementary Data 2**-**Table 1 - Supplementary Data 5**).

### 3.1 α-diversity and β-diversity

**Figure 1:**
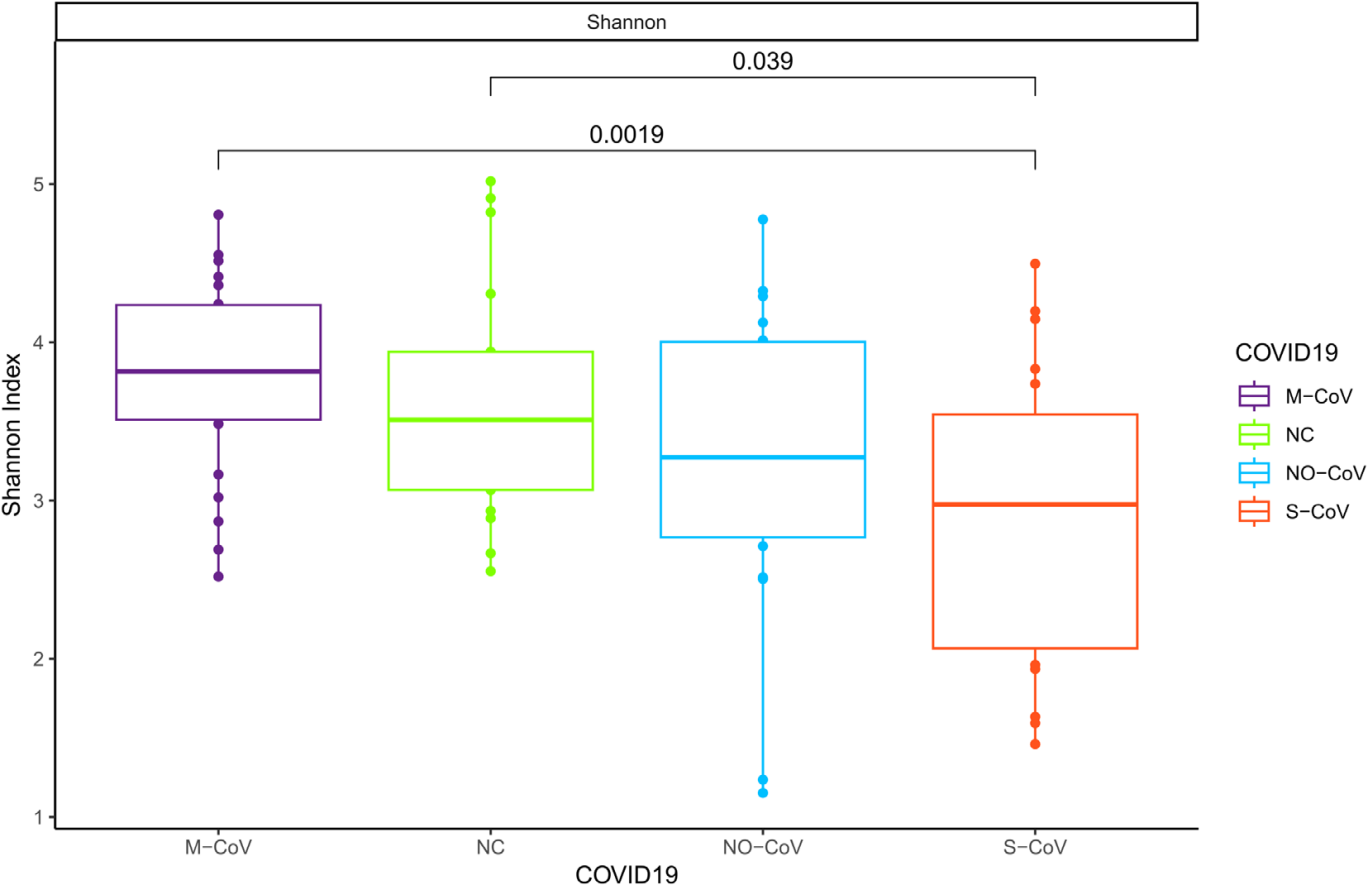
α-diversity analysis by Shannon index for COVID-19 groups. The Wilcoxon rank-sum test assessed statistical significance. M-CoV: moderate COVID-19; NC: Negative Control; NO-CoV: patients with pneumonia, ARDS, SpO2 ≥ 90% and a negative rt-qPCR test for SARS-CoV-2; S-CoV: severe COVID-19. The pairwise comparison shows statistical differences between M-CoV and S-CoV (p = 0.0019) and between S-CoV and NC (p = 0.039).

The S-CoV group presented the lowest α-diversity (mean = 2.90, standard error (SD) = 0.94). In the pairwise comparison, statistically significant differences were observed (**Figure 1**) between M-CoV and S-CoV (p = 0.0019) and between S-CoV and NC (p = 0.039). The α-diversity summarized statistics are presented in **Supplementary Material** (**Table 1 - Supplementary Data 6**).

No statistically significant difference was detected among groups for β-diversity ( Data Sheet 3 - Exploratory Analysis, 2.0 - Beta Diversity).

### 3.2 Differential abundance analysis

**Figure 2:**
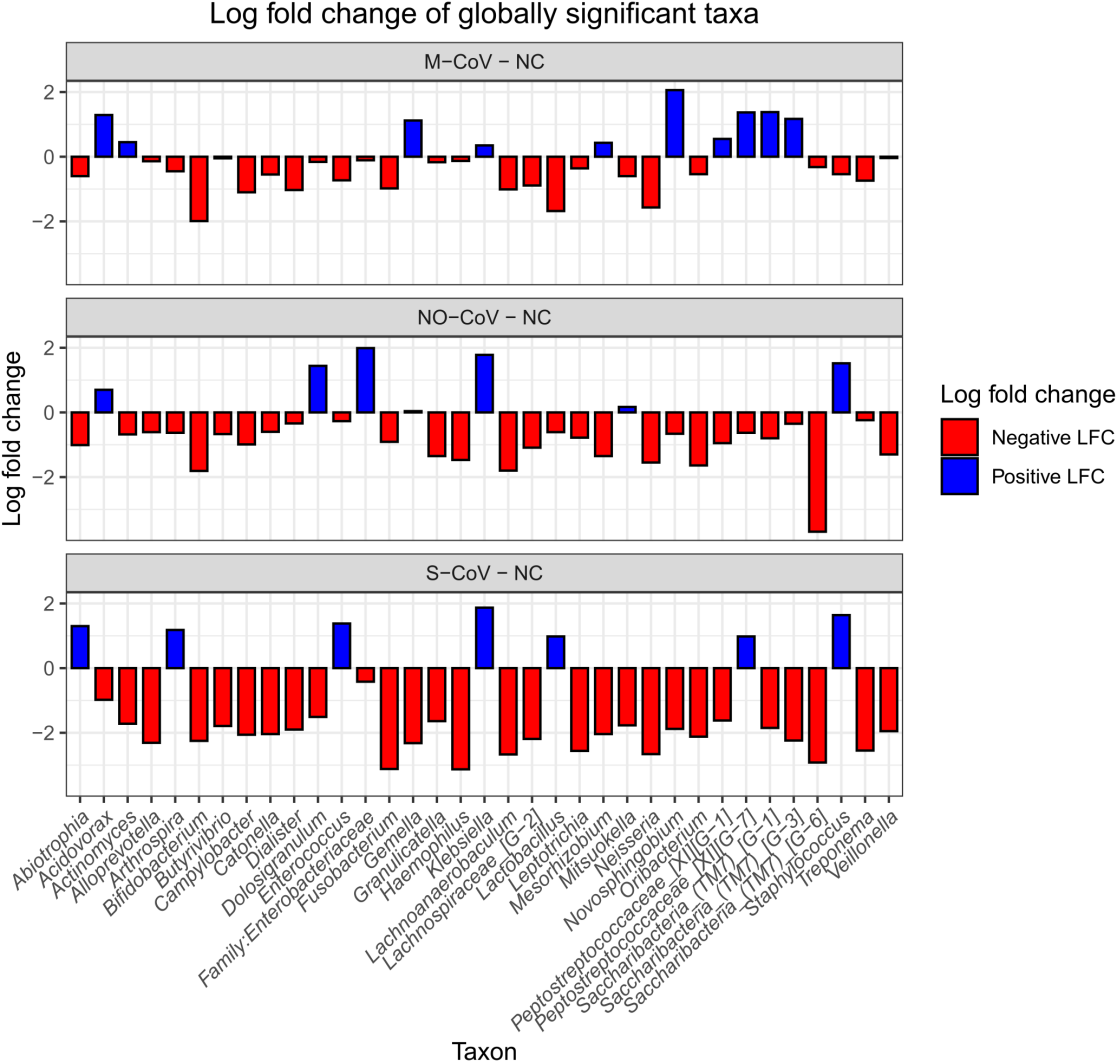
Log fold change (LFC) of globally significant taxa with NC as the reference group. M-CoV: moderate COVID-19; NC: Negative Control; NO-CoV: patients with pneumonia, ARDS, SpO2 ≥ 90% and a negative rt-qPCR test for SARS-CoV-2; S-CoV: severe COVID-19. Negative LFCs are colored red, and positive LFCs are colored blue.

Several taxa presented global differential abundance in comparison with the NC group. The genera *Abiotrophia* sp., *Arthrospira* sp., *Enterococcus* sp. and *Lactobacillus* sp. showed positive differential abundance for S-CoV while presenting negative for M-CoV and NO-CoV. Conversely, *Acidovorax* sp. showed positive differential abundance for M-CoV and NO-CoV while presenting negative for S-CoV. *Gemella* sp., *Mesorhizobium* sp., *Novosphingobium* sp., and *Saccharibacteria*_(TM7) were enriched only in M-CoV, while *Dolosigranulum* sp. and family *Enterobacteriaceae* were only increased in NO-CoV (**Figure 2**).

The S-CoV group presented the most significant reduced differential abundance of eubiotic taxa and enrichment of potential pathogens such as *Enterococcus* sp., *Klebsiella* sp., and *Staphylococcus* sp. (**Figure 2**).

**Figure 3:**
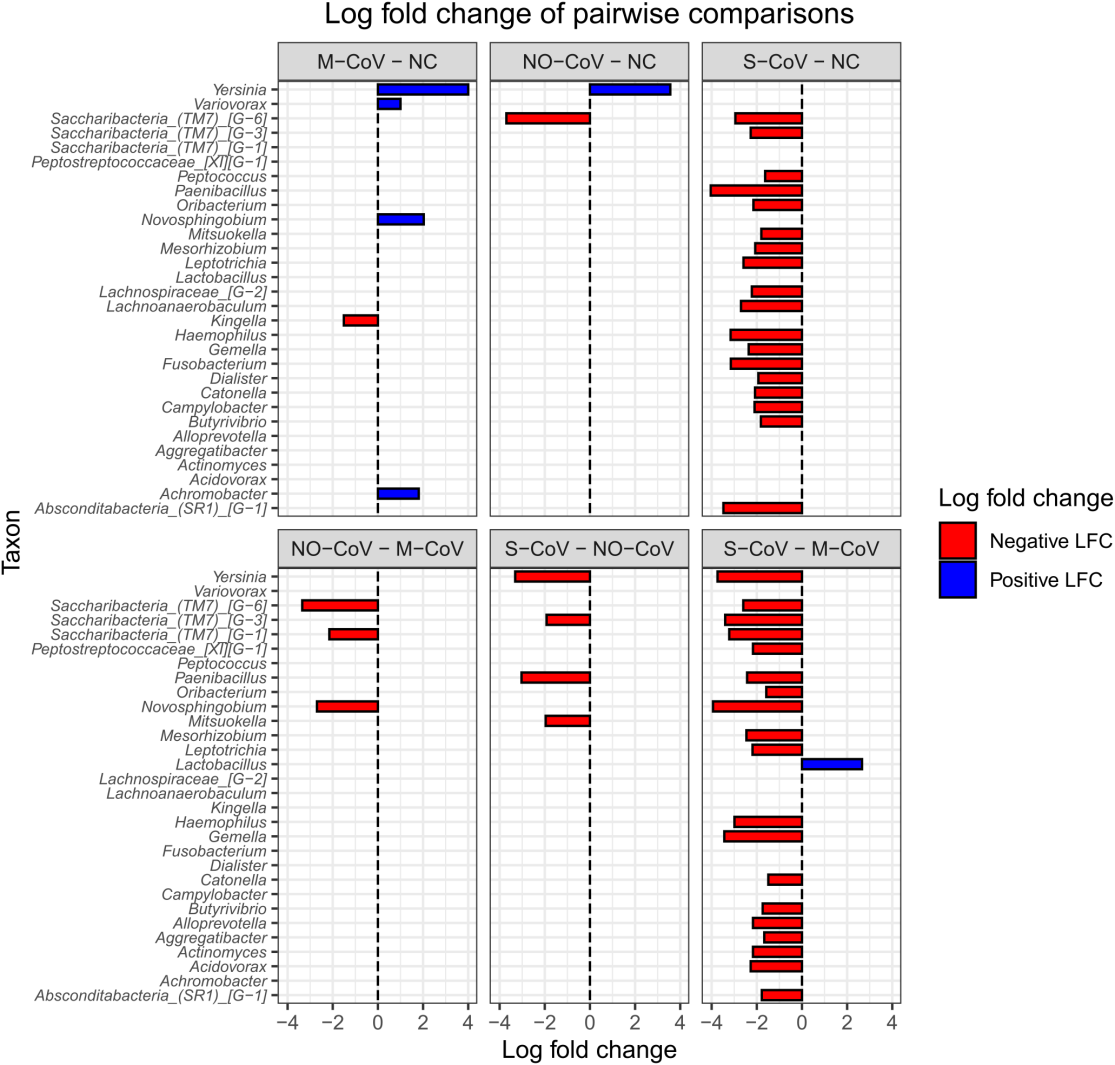
Log fold change for multiple pairwise comparisons between experimental groups. Only statistically significant results (p-adjusted) are demonstrated in the plot. M-CoV: moderate COVID-19; NC: Negative Control; NO-CoV: patients with pneumonia, ARDS, SpO2 ≥ 90% and a negative rt-qPCR test for SARS-CoV-2; S-CoV: severe COVID-19. Negative LFCs are colored red, and positive LFCs are colored blue.

In the pairwise comparisons (**Figure 3**), differential abundance in taxa was observed among all COVID-19 groups, with the most significant differences observed between S-CoV and M-CoV (*Lactobacillus* sp., presented positive differential abundance for S-CoV) as well as S-CoV and NC group (no taxon presented positive LFC for S-CoV). All data generated from the differential abundance analysis are presented in **Supplementary Material** (**Table 1 - Supplementary Data 7**-**Table 1 - Supplementary Data 10**).

### 3.3 Metabolic prediction

**Figure 4:**
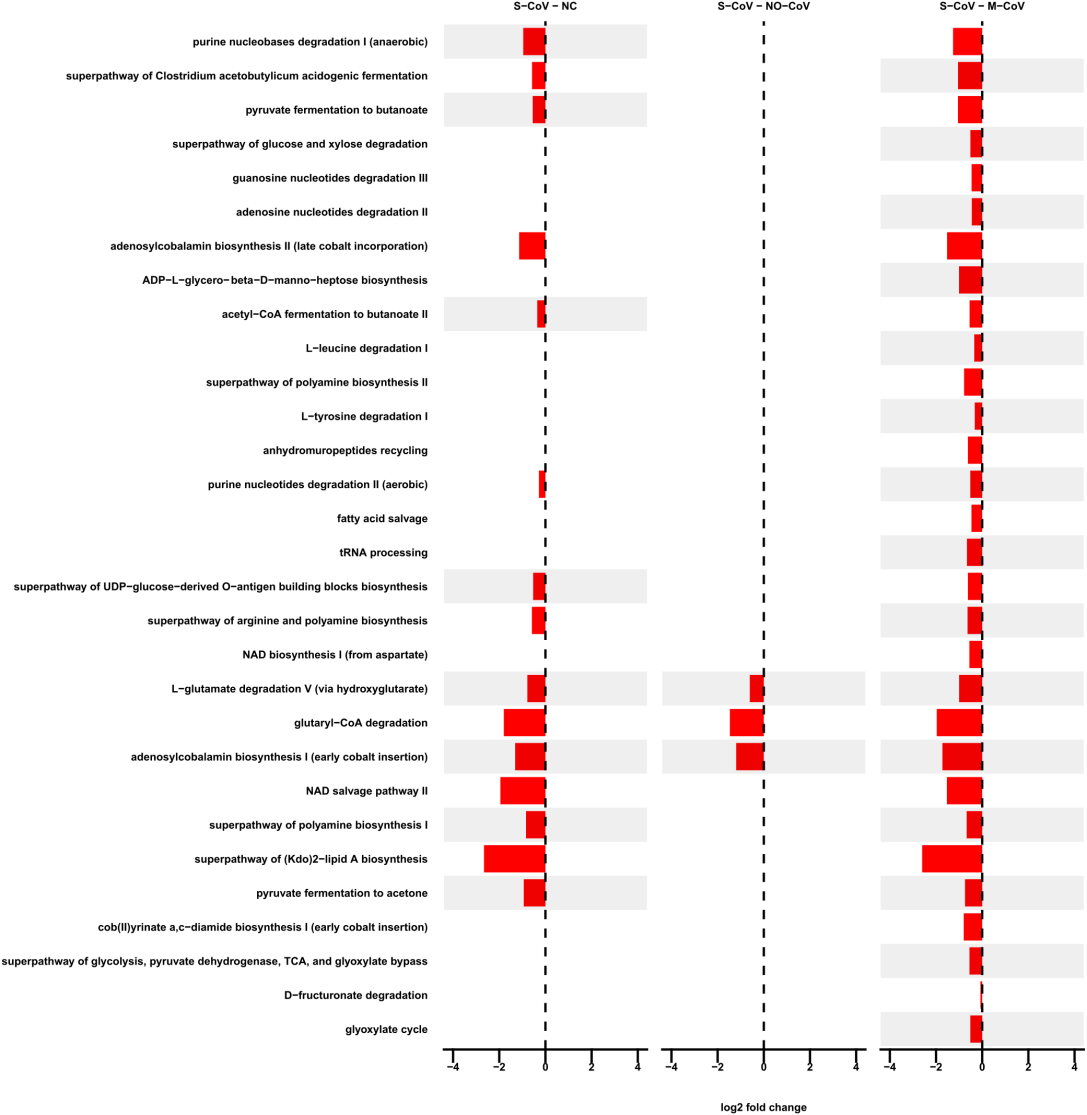
Differential abundance of metabolic pathways. Only statistically significant results (p-adjusted) for the top 30 pathways with the highest relative abundance are presented. NC - Negative Control; M-CoV - moderate COVID-19; NO-CoV - patients with pneumonia, ARDS, SpO2 ≥ 90% and a negative rt-qPCR test for SARS-CoV-2; S-CoV - severe COVID-19. Negative LFCs are colored red, and positive LFCs are colored blue. .

A total of 146 pathways presented statistically significant results in the differential abundance analysis (**Table 1-Supplementary Data 11, Table 1-Supplementary Data 12**), and the 30 metabolic pathways with the highest relative abundance are illustrated in **Figure 4**. It is possible to highlight that adenosylcobalamin (vitamin B12) and demethylmenaquinol−6 (vitamin K) biosynthesis; aromatic compounds degradation; purine nucleobases degradation; amino acid degradation; SCFA metabolism; sugar biosynthesis/lipopolysaccharide (LPS) biosynthesis/O-Antigen biosynthesis; steroid hormones degradation; amines and polyamines biosynthesis; amide, amidine, amine, and polyamine degradation; sugar degradation; tRNA processing; NAD metabolism; and protein N−glycosylation are among the most differentially abundant predicted ontologies.

The most pronounced differences were observed between S-CoV and M-CoV, followed by S-CoV and NC. All the data generated from the pathways’ differential abundance are presented in **Supplementary Material** (**Table 1 - Supplementary Data 11**,**Table 1 - Supplementary Data 12**).

### 3.4 Pairwise log-ratio exploratory analysis

Differentially abundant taxa between experimental groups also presented significant associations with clinical variables (**Supplementary Material - Table 2**). The pair (s) of taxa whose log-ratio was more associated with the variables, the name of the most important taxa, and the correlation-like plot that provides the association of each pairwise log-ratio and their respective correlation values with each clinical variable are presented in the Data Sheet 3 – 5.0 Coda4Microbiome.

## 4 Discussion

Our study evaluated the upper respiratory tract microbiome in COVID-19 patients, revealing that severe cases (S-CoV) exhibit a statistically significant reduction in α-diversity compared to mild cases (M-CoV) and non-COVID patients (NC). This observation is consistent with previous reports linking reduced diversity to increased COVID-19 severity (Mostafa et al., 2020; Soffritti et al., 2021; Ke et al., 2022). Although β-diversity did not show significant differences - likely due to confounding factors such as antibiotic use and comorbidities (Maes et al.; Soffritti et al.; Nardelli et al., 2021) - the overall disturbance in microbial homeostasis appears to be associated with a more severe disease phenotype.

The URT microbiome was dominated by genera such as *Streptococcus* sp., *Prevotella* sp., *Staphylococcus* sp., *Propionibacterium* sp., *Leptotrichia* sp., *Rothia* sp., *Dolosigranulum* sp., *Haemophilus* sp., *Moraxella* sp., *Veillonella* sp., and *Corynebacterium* sp (Man et al., 2017; Wypych et al., 2019). Among these, certain opportunistic species - such as *S. aureus* and *H. influenzae -* are well-known contributors to pneumonia in critically ill COVID-19 patients (Bai et al., 2022). Notably, *Abiotrophia* sp. was enriched exclusively in the S-CoV group, which is positively correlated with COVID-19 severity (Hernández-Terán et al., 2021; Soffritti et al., 2021). Additionally, taxa like *Saccharibacteria* (specifically, *Saccharibacteria*_[(TM7]_(G1), (G3) and (G6))) were found in higher abundance in NC and paucisymptomatic patients, whereas ‘non-*Saccharibacteria*’ underrepresented taxa such as *Mesorhizobium* sp., *Oribacterium* sp., and *Dolosigranulum* sp. were reduced in S-CoV and associated with inflammatory biomarkers, suggesting a protective effect (Bosch et al., 2016; Brugger et al., 2016).

A group of beneficial taxa - particularly members of the *Lachnospiraceae* family (e.g., *Catonella* sp., *Lachnoanaerobaculum* sp., *Butyrivibrio* sp.) - were enriched in the NC group and inversely related to disease severity. These butyrate-producing bacteria have recognized roles in immune modulation and anti-inflammatory activity (Al Bataineh et al., 2021), suggesting that their depletion in S-CoV could impair protective mechanisms against SARS-CoV-2 infection. Similarly, *Neisseria* sp. - which may enhance anti-SARS-CoV-2 antibody production through interactions with T- and B-cells (Hung and Christodoulides, 2013; Santana-Mederos et al., 2022) - displayed a reduced differential abundance in S-CoV, further linking microbial composition to immune functionality.

In parallel with taxonomic alterations, our metabolic predictions revealed significant changes in key pathways that appear to reflect the functional impact of microbial dysbiosis in COVID-19. One set of interrelated findings involves the modulation of steroid hormone synthesis and vitamin metabolism. The S-CoV group showed a reduced abundance of pathways involved in steroid hormone synthesis (including androstenedione degradation), a process regulated by ACTH and the renin-angiotensin-aldosterone system (RAAS) that is linked to COVID-19 severity (Pal, 2020; Sezer et al., 2022). This observation is paralleled by the depletion of eubiotic taxa from the order *Corynebacteriales* (related to vitamin B12 biosynthesis) and the family *Propionibacteriaceae* (linked to vitamin B12, vitamin K biosynthesis, and SCFA metabolism) in S-CoV. In contrast, taxa such as *Novosphingobium* sp. were enriched only in M-CoV and correlated with critical inflammatory biomarkers, suggesting their possible role in mitigating severe inflammation.

Another functionally connected group of pathways relates to mucin metabolism and immune modulation. Several genera within the *Peptostreptococcaceae* family, known for cleaving and transporting mucin-associated monosaccharides (Flynn et al., 2016), were reduced in S-CoV and NC compared to M-CoV. This reduction aligns with observed decreases in the superpathway of fucose and rhamnose degradation—processes that influence mucin O-glycosylation and subsequent IgG Fc fucosylation (Bennett et al., 2012; Flynn et al., 2016; Wlodarska et al., 2017; Wang, 2019; Chakraborty et al., 2021). Such alterations may disrupt immune system activation and inflammatory signaling, further exacerbating disease severity.

Further, metabolic pathways involved in glycosaminoglycan (GAG) biosynthesis - evidenced by decreased superpathway of glucose and xylose degradation - may enhance the availability of xylose for GAG synthesis. This is of particular interest given that both *Enterococcus* sp. and *Lactobacillus* sp. (which carry glycosaminoglycan genetic clusters; (Kawai et al., 2018)) were enriched in S-CoV, suggesting a potential role in viral attachment via the S protein and consequent lung inflammation (Cagno et al., 2019; Kim et al., 2020).

Additional metabolic disruptions include reductions in pathways related to aromatic compound degradation, purine nucleobase metabolism, and amino acid degradation (e.g., L-tryptophan, L-glutamate, and L-lysine). These changes have been implicated in altered inflammatory responses and immune regulation (Chakraborty et al., 2021; Wu et al., 2021; Casas-Sanchez et al., 2022; Kasahara et al., 2023). The diminished capacity for polyamine and arginine metabolism, which are linked to enhanced IgA responses and vascular function (Durante, 2013, Durante, 2022; Rodriguez et al., 2017; Falck-Jones et al., 2021; Martí I Líndez and Reith, 2021; Zhao et al., 2023), further underscores the potential for metabolic impairment to contribute to severe COVID-19 outcomes.

Moreover, the decreased predicted abundance of pathways related to the biosynthesis of key microbial-derived components, such as vitamin B12 (Tamura et al., 1999; Yoshii et al., 2019) and vitamin K (Ragab et al., 2020; Kudelko et al., 2021), as well as SCFA metabolism (Dalile et al., 2019; Ashique et al., 2022; Batista et al., 2022), suggests that the loss of these beneficial metabolites may impair immune regulation and anti-inflammatory responses. This is reinforced by the observed reduction in taxa associated with SCFA production, including members of *Lachnospiraceae* and *Propionibacteriaceae*.

Lastly, the observed decreases in lipopolysaccharide (LPS) biosynthesis pathways (Maldonado et al., 2016; Bradley et al., 2022), in NAD metabolism (Zheng et al., 2022) and tRNA processing (Katanski et al., 2022) further indicate that key inflammatory and metabolic responses are disrupted in severe COVID-19.

Collectively, the close interrelation between specific taxa and their metabolic functions suggests that dysbiosis in the URT may directly impact host immune responses. The depletion of beneficial microbes such as *Dolosigranulum* sp. and butyrate-producing *Lachnospiraceae*, along with impaired vitamin and SCFA biosynthesis, could compromise mucosal immunity and facilitate an exaggerated inflammatory response. In addition, alterations in steroid hormone synthesis pathways, mucin glycosylation, and polyamine metabolism likely contribute to the cytokine dysregulation observed in severe COVID-19. This complex network of taxon - metabolic interactions underscores the potential of the URT microbiome as a modifiable factor in the progression of COVID-19.

## 5 Conclusions

This study demonstrates that the primary differences in URT microbiome composition and functionality are associated with COVID-19 severity rather than with SARS-CoV-2 infection. Severe COVID-19 is characterized by a significant reduction in α-diversity, a depletion of protective eubiotic taxa, and widespread alterations in metabolic pathways essential for immune modulation and host homeostasis. Despite inherent limitations (cross-sectional design, not controlling for confounding factors as age, antibiotic use, comorbidities), these findings suggest that specific microbial taxa – such as *Lachnospiraceae, Propionibacteriaceae, Dolosigranulum* sp. - and their metabolites - such as SCFAs, vitamins B12 and K, and key amino acids - could serve as promising targets for therapeutic intervention. Despite inherent limitations, our results provide a foundation for future studies aimed at restoring microbial homeostasis to mitigate severe respiratory outcomes.

## Data Availability

The datasets presented in this study can be found in online repositories. The names of the repository/repositories and accession number(s) can be found in the article/**Supplementary Material**.
