# Supplementary material for: Unveiling the role of the upper respiratory tract microbiome in susceptibility and severity to COVID-19
Source: Front Cell Infect Microbiol. 2025 May 13;15:1531084. doi: 10.3389/fcimb.2025.1531084 (PMC12106449; doi:10.3389/fcimb.2025.1531084)
Supplement: Supplementary file 1 [file DataSheet1.pdf]

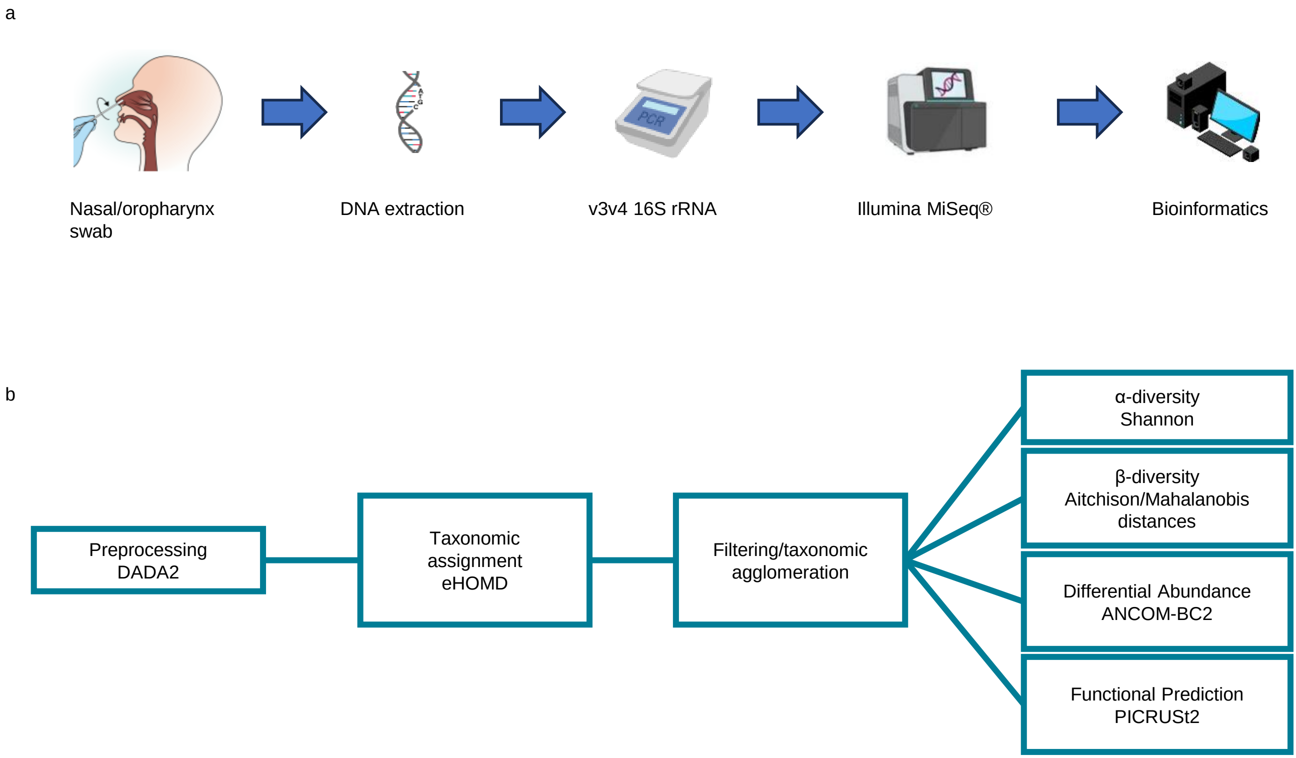

Flowchart of experimental design and bioinformatics. a. Experimental workflow from sample collection to bioinformatics analysis. b. Bioinformatics workflow from data preprocessing to final results.
