# Supplementary material for: Unveiling the role of the upper respiratory tract microbiome in susceptibility and severity to COVID-19
Source: Front Cell Infect Microbiol. 2025 May 13;15:1531084. doi: 10.3389/fcimb.2025.1531084 (PMC12106449; doi:10.3389/fcimb.2025.1531084)
Supplement: Supplementary file 2 [file DataSheet2.pdf]

UNVEILING THE ROLE OF THE UPPER RESPIRATORY TRACT MICROBIOME IN SUSCEPTIBILITY AND SEVERITY TO COVID-19

Otávio von Ameln Lovison

2024-11-13

Contents

0 - Prep 1
- 0.1 - Load libraries . . . 2
- 0.2 Download main data . . . 2
- 0.3 Preprocessing . . . 2
  - 0.3.1 Setting the paths . . . 2
  - 0.3.2 Forward Quality Plot . . . 2
  - 0.3.3 Reverse Quality Plot . . . 2
  - 0.3.4 Filter and trim . . . 5
  - 0.3.5 Dereplication . . . 5
  - 0.3.6 Learning errors . . . 5
  - 0.3.7 Forward error rates . . . 5
  - 0.3.8 Reverse error rates . . . 7
  - 0.3.9 Sample Inference . . . 8
  - 0.3.10 Merge paired reads . . . 8
  - 0.3.11 Construct Sequence Table . . . 8
  - 0.3.12 Remove chimeras and check the output . . . 8
  - 0.3.13 Remove non-target-length sequences . . . 8
  - 0.3.14 Track reads through the pipeline . . . 8
  - 0.3.15 Assign taxonomy . . . 9
  - 0.3.16 Metadata engineering . . . 9
  - 0.3.17 Construct the phyloseq object . . . 10

0 - Prep

The data used for this analysis is from the project 'Proteomics and Metagenomics for Identification and Characterization of COVID-19 Biomarkers', ethics approval 4.355.906, Hospital de Clinicas de Porto Alegre (HCPA). The bioinformatics analyses were performed in the Bioinformatics Core of HCPA. This document presents the microbiome analysis workflow for this project.

In this analysis we include 79 combined nasal and oropharynx swabs from HCPA biobank, collected to perform rt-qPCR for SARS-CoV-2 detection. The samples were selected using COVID-19 severity class (WHO, 2020), as follows: Group 1 (M-CoV, n = 22): positive rt-qPCR for SARS-CoV-2 - COVID-19 - moderate; Group 2 (NO-CoV, n = 19, control group): negative rt-qPCR for SARS-CoV-2 (confirmed with a second test), previously classified as moderate COVID-19 by the physician; Group 3 (S-CoV, n = 20): positive rt-qPCR for SARS-CoV-2 - COVID-19 - severe/critical; Group 4 (NC, n = 18, control group): asymptomatic, highly exposed patients and healthcare workers, who tested negative by rt-qPCR for SARS-CoV-2 screening.

0.1 - Load libraries

```
rm(list = ls())
library(knitr)
library(phyloseq)
library(ggplot2)
library(gridExtra)
library(dada2)
library(rio)
```

0.2 Download main data

```
###Raw data (fastq files)
download.file('https://www.ncbi.nlm.nih.gov/sra/PRJNA1133028',sep = '/'))

# clean environment
rm(list = ls(all = TRUE))
```

0.3 Preprocessing

0.3.1 Setting the paths

```
set.seed(100)
load('Lovison_etal_2023_COVID19_16S_preprocessing.RData')
#Setting the paths and listing files
miseq_path <-
  file.path("/home/metagenomica/LABRESIS/OtavioLovison/COVID19Microbiomes/FASTQ")
filt_path <-
  file.path("/home/metagenomica/LABRESIS/OtavioLovison/COVID19Microbiomes/FASTQ/
            filtered")
fns <-
  sort(list.files("/home/metagenomica/LABRESIS/OtavioLovison/COVID19Microbiomes/FASTQ",
                  full.names = TRUE))
fnFs <- fns[grepl("R1", fns)]
fnRs <- fns[grepl("R2", fns)]
```

0.3.2 Forward Quality Plot

```
ii <- sample(length(fnFs), 1)
for(i in ii) { print(plotQualityProfile(fnFs[1:6]) + ggtitle("Fwd")) }
```

In the grayscale, a heatmap illustrates the frequency of each quality score at every base position. The green line represents the median quality score at each position, while the orange lines depict the quartiles of the quality score distribution. Additionally, the red line indicates the scaled proportion of reads that extend to, at least, that particular position.

0.3.3 Reverse Quality Plot

```
for(i in ii) { print(plotQualityProfile(fnRs[1:6]) + ggtitle("Rev")) }
```

In the grayscale, a heatmap illustrates the frequency of each quality score at every base position. The green line represents the median quality score at each position, while the orange lines depict the quartiles of the

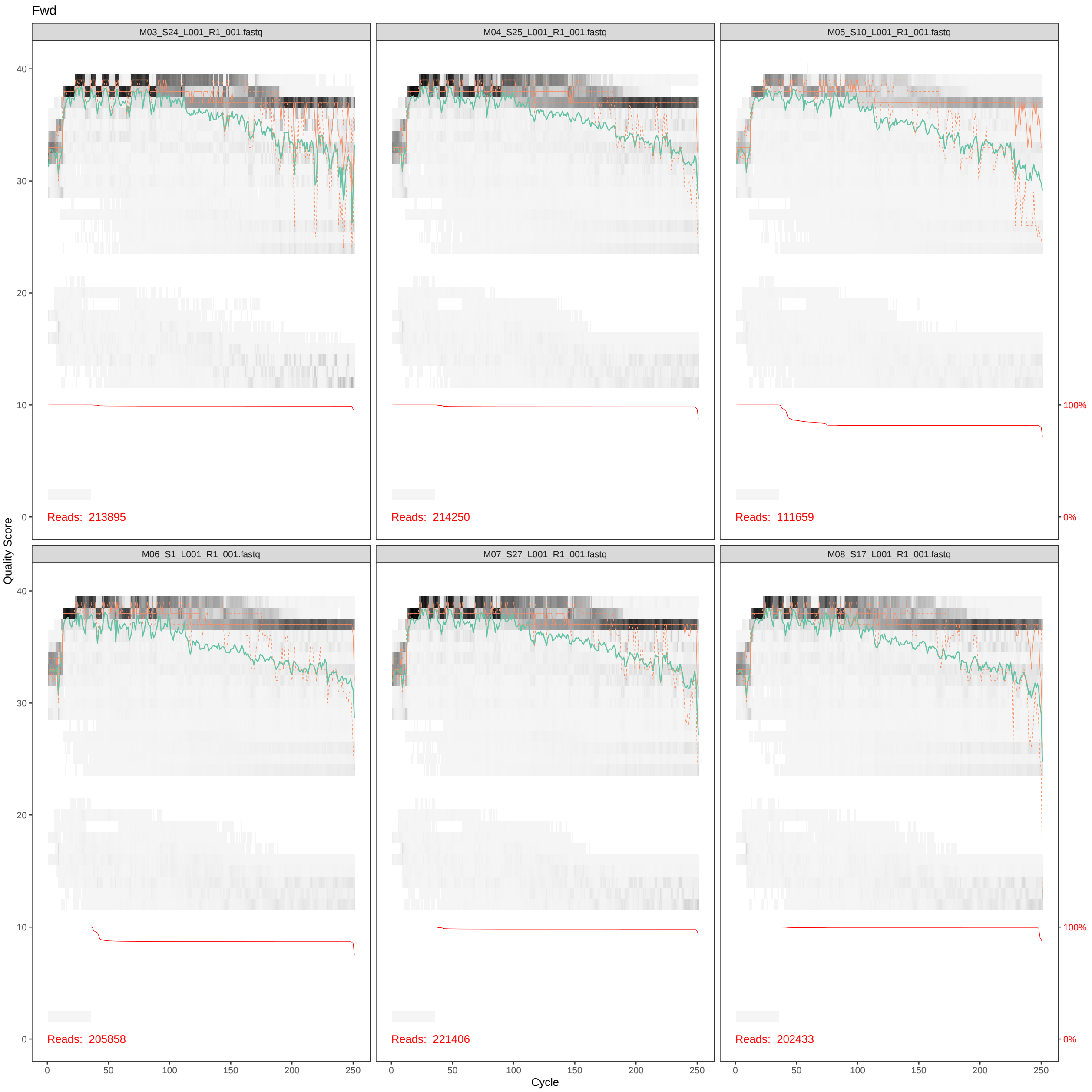

Figure 1: Forward reads quality plot

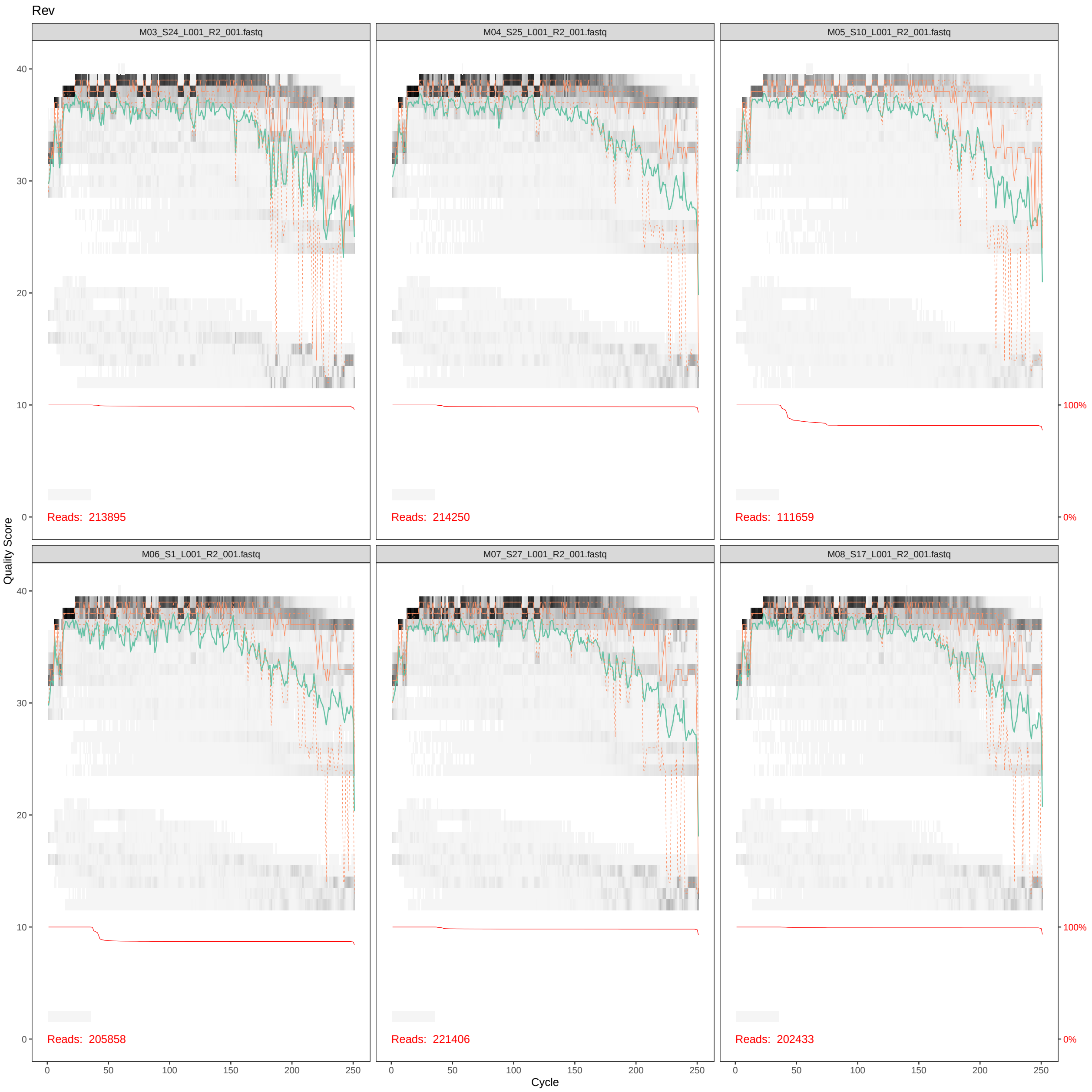

Figure 2: Reverse reads quality plot.

quality score distribution. Additionally, the red line indicates the scaled proportion of reads that extend to, at least, that particular position.

0.3.4 Filter and trim

```
if(!file_test("-d", filt_path)) dir.create(filt_path)
filtFs <- file.path(filt_path, basename(fnFs))
filtRs <- file.path(filt_path, basename(fnRs))

out <- data.frame(reads.in = c(), reads.out = c(), sample = c())
for(i in seq_along(fnFs)) {
  z <- fastqPairedFilter(c(fnFs[[i]], fnRs[[i]]),
                         c(filtFs[[i]], filtRs[[i]]),
                         trimLeft=c(17, 21),
                         truncLen=c(240, 240),
                         maxN=0, maxEE=c(2,2), rm.phix = TRUE,
                         compress=TRUE, multithread=TRUE)
  out <- rbind(out, z)
}

save(file = "/home/metagenomica/LABRESIS/OtavioLovison/COVID19Microbiomes/
     Lovison_etal_2023_COVID19_16S_preprocessing.RData",list =
       c('out', 'miseq_path','filt_path','fns','fnFs', 'fnRs', 'ii', 'filtFs', 'filtRs'))
#rm(list = ls())
```

0.3.5 Dereplication

```
load('Lovison_etal_2023_COVID19_16S_preprocessing.RData')
derepFs <- derepFastq(filtFs)
derepRs <- derepFastq(filtRs)
sam.names <- sapply(strsplit(basename(fnFs), "_"), `[`, 1)
names(derepFs) <- sam.names
names(derepRs) <- sam.names
save(file = "/home/metagenomica/LABRESIS/OtavioLovison/COVID19Microbiomes/
     Lovison_etal_2023_COVID19_16S_preprocessing.RData",list =
       c('out', 'miseq_path','filt_path','fns','fnFs', 'fnRs', 'ii', 'filtFs',
         'filtRs', 'sam.names', 'derepFs', 'derepRs'))
rm(list = ls())
```

0.3.6 Learning errors

```
load('Lovison_etal_2023_COVID19_16S_preprocessing.RData')
ddF <- dada(derepFs[1:79], err=NULL, selfConsist=TRUE, MAX_CONSIST = 20)
ddR <- dada(derepRs[1:79], err=NULL, selfConsist=TRUE, MAX_CONSIST = 20)
save(file = "/home/metagenomica/LABRESIS/OtavioLovison/COVID19Microbiomes/
     Lovison_etal_2023_COVID19_16S_preprocessing.RData",list =
       c('out', 'miseq_path','filt_path','fns','fnFs', 'fnRs', 'ii', 'filtFs',
         'filtRs', 'sam.names', 'derepFs', 'derepRs', 'ddF', 'ddR'))
```

0.3.7 Forward error rates

```
plotErrors(ddF)
```

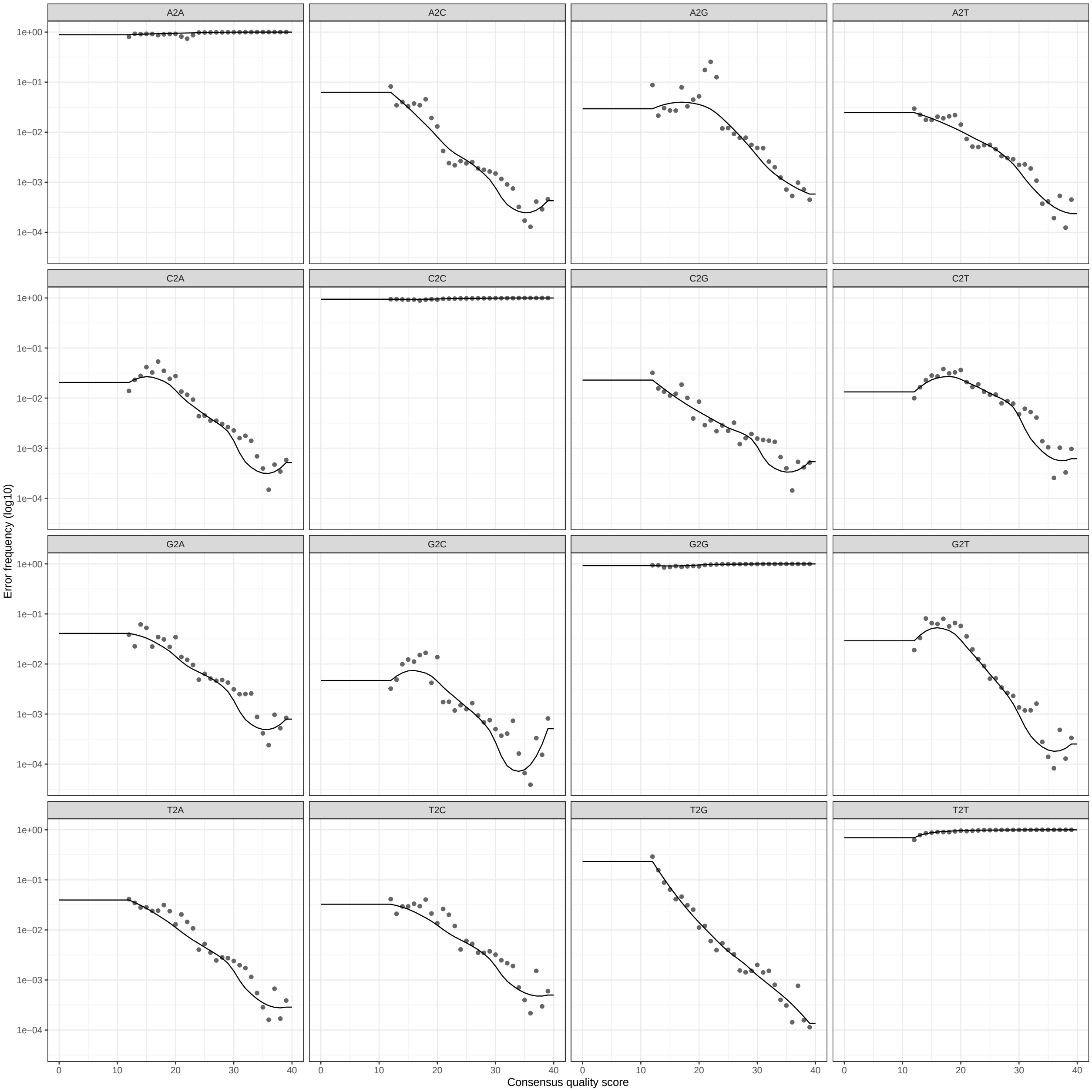

Figure 3: Forward reads error rates.

Points are the observed error rates for each consensus quality score. The black line shows the estimated error rates after convergence of the machine-learning algorithm.

0.3.8 Reverse error rates

```
plotErrors(ddR)
```

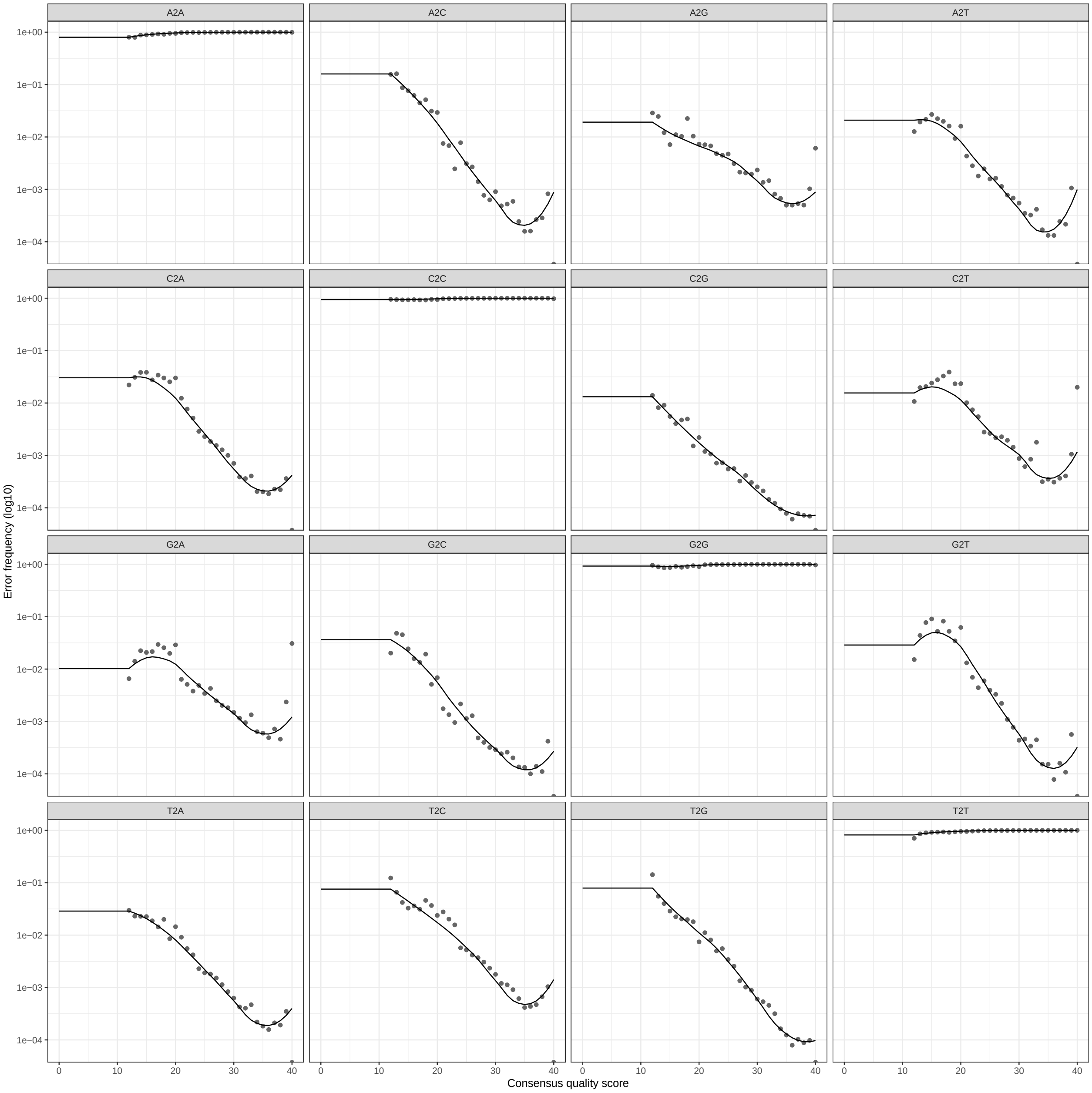

Figure 4: Reverse reads error rates.

```
#rm(list = ls())
```

Points are the observed error rates for each consensus quality score. The black line shows the estimated error rates after convergence of the machine-learning algorithm.

0.3.9 Sample Inference

```
load('Lovison_etal_2023_COVID19_16S_preprocessing.RData')
dadaFs <- dada(derepFs, err=ddF[[1]]$err_out, pool=TRUE, multithread = TRUE)
dadaRs <- dada(derepRs, err=ddR[[1]]$err_out, pool=TRUE, multithread = TRUE)
save(file = "/home/metagenomica/LABRESIS/OtavioLovison/COVID19Microbiomes/
     Lovison_etal_COVID19_preprocessing.RData",list =
       c('out','miseq_path','filt_path','fns','fnFs', 'fnRs', 'ii', 'filtFs',
         'filtRs', 'sam.names', 'derepFs', 'derepRs', 'ddF', 'ddR', 'dadaFs', 'dadaRs'))
rm(list = ls())
```

0.3.10 Merge paired reads

```
load('Lovison_etal_2023_COVID19_16S_preprocessing.RData')
mergers <- mergePairs(dadaFs, derepFs, dadaRs, derepRs, verbose = TRUE)
save(file = "Lovison_etal_2023_COVID19_16S_preprocessing.RData",list =
       c('out','miseq_path','filt_path','fns','fnFs', 'fnRs', 'ii', 'filtFs',
         'filtRs', 'sam.names', 'derepFs', 'derepRs', 'ddF', 'ddR', 'dadaFs',
         'dadaRs', 'mergers'))
rm(list = ls())
```

0.3.11 Construct Sequence Table

```
load('Lovison_etal_2023_COVID19_16S_preprocessing.RData')
seqtab.all <- makeSequenceTable(mergers[!grepl("Mock", names(mergers))])
dim(seqtab.all)
save(file = "Lovison_etal_2023_COVID19_16S_preprocessing.RData",
     list=c('out','miseq_path','filt_path','fns','fnFs', 'fnRs', 'ii', 'filtFs',
            'filtRs', 'sam.names', 'derepFs', 'derepRs', 'ddF', 'ddR', 'dadaFs',
            'dadaRs', 'mergers', 'seqtab.all'))
```

0.3.12 Remove chimeras and check the output

```
seqtab2 <- removeBimeraDenovo(seqtab.all, multithread=TRUE, verbose = TRUE)
sum(seqtab2)/sum(seqtab.all)
save(file = "Lovison_etal_2023_COVID19_16S_preprocessing.RData",list=
       c('out','miseq_path','filt_path','fns','fnFs', 'fnRs', 'ii', 'filtFs',
         'filtRs', 'sam.names', 'derepFs', 'derepRs', 'ddF', 'ddR', 'dadaFs',
         'dadaRs', 'mergers', 'seqtab.all', 'seqtab2'))
rm(list = ls())
```

0.3.13 Remove non-target-length sequences

```
load('Lovison_etal_2023_COVID19_16S_preprocessing.RData')
table(nchar(getSequences(seqtab2)))
seqtab <- seqtab2[,nchar(colnames(seqtab2)) %in% seq(398,430)]
table(nchar(getSequences(seqtab)))
```

0.3.14 Track reads through the pipeline

```
getN <- function(x) sum(getUniques(x))
track <- cbind(out, sapply(dadaFs, getN),sapply(dadaRs, getN),
```

```
                sapply(mergers, getN), rowSums(seqtab))
colnames(track) <- c("input", "filtered", "denoisedF", "denoisedR", "merged",
                      "nonchim")
export(track, "Reads through the pipeline.xlsx", rowNames = TRUE)
```

0.3.15 Assign taxonomy

```
taxtab <- assignTaxonomy(seqtab, "/home/metagenomica/Bancos_de_dados/eHOMD/
                          eHOMD_RefSeq_dada2_V15.22.fasta.gz")
taxtab <- addSpecies(taxtab, "/home/metagenomica/Bancos_de_dados/eHOMD/
                      eHOMD_RefSeq_dada2_assign_species_V15.22.fasta.gz")
save(file = "/home/metagenomica/LABRESIS/OtavioLovison/COVID19Microbiomes/
     Lovison_etal_2023_COVID19_preprocessing.RData",list=
       c('out','miseq_path','filt_path','fns','fnFs', 'fnRs', 'ii', 'filtFs',
         'filtRs', 'sam.names', 'derepFs', 'derepRs', 'ddF', 'ddR', 'dadaFs',
         'dadaRs', 'mergers', 'seqtab.all', 'seqtab2', 'seqtab', 'taxtab'))
rm(list = ls())
```

0.3.16 Metadata engineering

```
# Load the required libraries
library(readxl)
library(dplyr)
library(tidyr)

# Read the first Excel file
curated_dataset <- read_excel("/home/metagenomica/LABRESIS/OtavioLovison/
                               curated_dataset.xlsx")

# Convert "Extraction_date" to date-time format
curated_dataset$Extraction_date <- as.POSIXct(curated_dataset$Extraction_date,
                                   format = "%Y-%m-%dT%H:%M:%S.%OS", tz = "UTC")

# Group the data by "BiobankID" and "Analyte",
# and create a new column with the rank of each "Extraction_date"
curated_dataset <- curated_dataset %>%
  group_by(BiobankID, Analyte) %>%
  mutate(rank = dense_rank(Extraction_date))

# Create a new column with the name of the "Analyte" followed by the rank
curated_dataset$Analyte_ranked <- paste0(curated_dataset$Analyte,
                                         " (", curated_dataset$rank, ")")

# Pivot the data to wide format based on the new "Analyte_ranked" column and the
#"Result" column
curated_dataset_wide <- spread(curated_dataset, key = "Analyte_ranked",
                               value = "Result")

# Read the second Excel file
Lovison_etal_metadata <- read_excel("/home/metagenomica/LABRESIS/OtavioLovison/
                                     Lovison_etal_metadata.xlsx")

#Converting the "BiobankID" in 'double' format
```

```
Lovison_etal_metadata$BiobankID <- as.double(Lovison_etal_metadata$BiobankID)

# Merge the two data frames by "BiobankID"
merged_data <- left_join(Lovison_etal_metadata, curated_dataset_wide,
                         by = "BiobankID")

#Removing some columns
merged_data <- select(merged_data, -Analyte, -rank, -Extraction_date,
                      -CollectionDate, -Exam, -HospitalLocation)

#Summarize data
summarized_data <- merged_data %>%
  group_by(sampleID) %>%
  summarize(across(where(is.numeric), mean, na.rm = TRUE), .groups = "drop")

summarized_data <- inner_join(Lovison_etal_metadata, summarized_data,
                              by = "sampleID")

#Removing low quality samples from metadata to match for phyloseq object
summarized_data_filtered <- summarized_data %>%
  filter(sampleID != "M02", sampleID != "M09", sampleID != "M49",
         sampleID != "M71", sampleID != "M79")

# Export the merged data to a CSV file
write.csv(summarized_data_filtered, file = "summarized_data_filtered.csv",
          sep=",", dec=".", row.names = FALSE)

#Importing the manually curated summarized data
samdf <- read.csv("summarized_data_filtered2.csv")
rownames(samdf) <- samdf$sampleID
```

0.3.17 Construct the phyloseq object

```
#Producing ps object
ps <- phyloseq(tax_table(taxtab),
               sample_data(samdf),
               otu_table(seqtab, taxa_are_rows = FALSE))
ps
saveRDS(ps, file = "ps.rds")

# Renaming sequences for better plotting
dna <- Biostrings::DNAStringSet(taxa_names(ps))
names(dna) <- taxa_names(ps)
ps.dna <- merge_phyloseq(ps, dna)
taxa_names(ps.dna) <- paste0("ASV", seq(ntaxa(ps.dna)))
ps.dna

saveRDS(ps.dna, file = "ps.dna.rds")

rm(list = ls())

ps <- readRDS("ps.rds")
ps.dna <- readRDS("ps.dna.rds")
```

```r
save(file = "Lovison_etal_2023_COVID19_RAWdata.RData",list = c('ps', 'ps.dna'))
```

```r
sessionInfo()
```

```
## R version 4.4.1 (2024-06-14)
## Platform: x86_64-pc-linux-gnu
## Running under: Ubuntu 22.04.5 LTS
##
## Matrix products: default
## BLAS:   /usr/lib/x86_64-linux-gnu/blas/libblas.so.3.10.0
## LAPACK: /usr/lib/x86_64-linux-gnu/lapack/liblapack.so.3.10.0
##
## locale:
##  [1] LC_CTYPE=pt_BR.UTF-8       LC_NUMERIC=C
##  [3] LC_TIME=pt_BR.UTF-8        LC_COLLATE=pt_BR.UTF-8
##  [5] LC_MONETARY=pt_BR.UTF-8    LC_MESSAGES=pt_BR.UTF-8
##  [7] LC_PAPER=pt_BR.UTF-8       LC_NAME=C
##  [9] LC_ADDRESS=C               LC_TELEPHONE=C
## [11] LC_MEASUREMENT=pt_BR.UTF-8 LC_IDENTIFICATION=C
##
## time zone: America/Sao_Paulo
## tzcode source: system (glibc)
##
## attached base packages:
## [1] stats     graphics  grDevices utils     datasets  methods   base
##
## other attached packages:
## [1] rio_1.2.2       dada2_1.32.0    Rcpp_1.0.13     gridExtra_2.3
## [5] ggplot2_3.5.1   phyloseq_1.48.0 knitr_1.49
##
## loaded via a namespace (and not attached):
##  [1] bitops_1.0-8                deldir_2.0-4
##  [3] permute_0.9-7               rlang_1.1.4
##  [5] magrittr_2.0.3              ade4_1.7-22
##  [7] matrixStats_1.3.0           compiler_4.4.1
##  [9] mgcv_1.9-1                  png_0.1-8
## [11] vctrs_0.6.5                 reshape2_1.4.4
## [13] stringr_1.5.1               pwalign_1.0.0
## [15] pkgconfig_2.0.3             crayon_1.5.2
## [17] fastmap_1.2.0               XVector_0.44.0
## [19] labeling_0.4.3              utf8_1.2.4
## [21] Rsamtools_2.20.0            rmarkdown_2.29
## [23] UCSC.utils_1.0.0            tinytex_0.54
## [25] xfun_0.49                   zlibbioc_1.50.0
## [27] GenomeInfoDb_1.40.1         jsonlite_1.8.8
## [29] biomformat_1.32.0           rhdf5filters_1.16.0
## [31] DelayedArray_0.30.1         Rhdf5lib_1.26.0
## [33] BiocParallel_1.38.0         jpeg_0.1-10
## [35] parallel_4.4.1              cluster_2.1.6
## [37] R6_2.5.1                    stringi_1.8.4
## [39] RColorBrewer_1.1-3          GenomicRanges_1.56.1
## [41] SummarizedExperiment_1.34.0 iterators_1.0.14
## [43] IRanges_2.38.1              Matrix_1.7-1
## [45] splines_4.4.1               igraph_2.0.3
```

```
## [47] tidyselect_1.2.1          rstudioapi_0.15.0
## [49] abind_1.4-5               yaml_2.3.8
## [51] vegan_2.6-6.1             codetools_0.2-19
## [53] hwriter_1.3.2.1           lattice_0.22-5
## [55] tibble_3.2.1              plyr_1.8.9
## [57] Biobase_2.64.0            withr_3.0.1
## [59] ShortRead_1.62.0          evaluate_0.23
## [61] survival_3.7-0            RcppParallel_5.1.9
## [63] Biostrings_2.72.1         pillar_1.9.0
## [65] MatrixGenerics_1.16.0     foreach_1.5.2
## [67] stats4_4.4.1              generics_0.1.3
## [69] S4Vectors_0.42.1          munsell_0.5.1
## [71] scales_1.3.0              glue_1.7.0
## [73] tools_4.4.1               interp_1.1-6
## [75] data.table_1.15.0         GenomicAlignments_1.40.0
## [77] rhdf5_2.48.0              grid_4.4.1
## [79] ape_5.8                   latticeExtra_0.6-30
## [81] colorspace_2.1-1          nlme_3.1-165
## [83] GenomeInfoDbData_1.2.12   cli_3.6.3
## [85] fansi_1.0.6               S4Arrays_1.4.1
## [87] dplyr_1.1.4               gtable_0.3.5
## [89] digest_0.6.34             BiocGenerics_0.50.0
## [91] SparseArray_1.4.8         farver_2.1.2
## [93] htmltools_0.5.7           multtest_2.60.0
## [95] lifecycle_1.0.4           httr_1.4.7
## [97] MASS_7.3-61
```
