# Supplementary material for: Unveiling the role of the upper respiratory tract microbiome in susceptibility and severity to COVID-19
Source: Front Cell Infect Microbiol. 2025 May 13;15:1531084. doi: 10.3389/fcimb.2025.1531084 (PMC12106449; doi:10.3389/fcimb.2025.1531084)
Supplement: Supplementary file 3 [file DataSheet3.pdf]

UNVEILING THE ROLE OF THE UPPER RESPIRATORY TRACT MICROBIOME IN SUSCEPTIBILITY AND SEVERITY TO COVID-19

Otávio von Ameln Lovison

2025-03-25

Contents

0 - Prep 1
0.1 Data preparation . . . 2

0.2 Microbiome overview 3
0.2.1 Ajusting for calculations and tables . . . 3
0.2.2 Dominant taxa and their overall relative abundance . . . 4
0.2.2.1 Tables . . . 4

1.0 Alpha-diversity 5
1.1 Alpha-diversity analysis by Shannon diversity index . . . 5

2.0 Beta-diversity 7
2.1 Principal Component Analysis (PCA) with the Aitchison (Euclidean) distance . . . 7
2.1.1 PERMANOVA for Aitchison distance . . . 8

3.0 Differential Abundance 10
3.1 Structural zeros on genus level . . . 10
3.2 ANCOM-BC2 Global test . . . 11
3.3 ANCOM-BC2 multiple pairwise comparisons on genus level . . . 12

4.0 Functional and metabolic prediction with PICRUSt2 (Phylogenetic Investigation of Communities by Reconstruction of Unobserved States) 13
4.1 Exporting the data for PICRUSt2 . . . 13
4.2 Metabolic prediction - MetaCyc Pathways . . . 14

5.0 Coda4Microbiome 44
5.1 Measure of Association . . . 44
5.1.1 Log-ratio exploratory analysis for a continuous variable . . . 44

6.0 Session info 106

0 - Prep

The data used for this analysis is from the project 'Proteomics and Metagenomics for Identification and Characterization of COVID-19 Biomarkers', ethics approval 4.355.906, Hospital de Clinicas de Porto Alegre (HCPA). The bioinformatics analyses were performed in the Bioinformatics Core of HCPA. This document presents the microbiome analysis workflow for this project.

In this analysis we include 79 combined nasal and oropharynx swabs from HCPA biobank, collected to perform rt-qPCR for SARS-CoV-2 detection. The samples were selected using COVID-19 severity class (WHO, 2020), as follows: Group 1 (M-CoV, n = 22): positive rt-qPCR for SARS-CoV-2 - COVID-19 - moderate; Group 2 (NO-CoV, n = 19, control group): negative rt-qPCR for SARS-CoV-2 (confirmed with a second test), previously classified as moderate COVID-19 by the physician; Group 3 (S-CoV, n = 20): positive rt-qPCR for SARS-CoV-2 - COVID-19 - severe/critical; Group 4 (NC, n = 18, control group): asymptomatic, highly exposed patients and healthcare workers, who tested negative by rt-qPCR for SARS-CoV-2 screening.

0.1 Data preparation

```
library(phyloseq)
library(ggpubr)
library(tidyverse)
library(rio)
ps.dna <- readRDS("ps.dna.rds")

rank_names(ps.dna)
```

```
## [1] "Kingdom" "Phylum"  "Class"   "Order"   "Family"  "Genus"   "Species"
```

```
table(tax_table(ps.dna)[, "Phylum"], exclude = NULL)
```

```
##
## Absconditabacteria_(SR1)           Actinobacteria            Bacteroidetes
##                       91                     7204                    10120
##               Chlamydiae                 Chlorobi              Chloroflexi
##                        2                        2                        4
##            Cyanobacteria               Firmicutes             Fusobacteria
##                       14                    21809                     1560
##     Gracilibacteria_(GN02)           Proteobacteria  Saccharibacteria_(TM7)
##                       15                     9061                      305
##             Spirochaetes            Synergistetes                     <NA>
##                      127                       41                     1383
```

```
ps0.dna <- subset_taxa(ps.dna, !is.na(Phylum) & !Phylum %in% c("", "uncharacterized"))

prevdf = apply(X = otu_table(ps0.dna),
               MARGIN = ifelse(taxa_are_rows(ps0.dna), yes = 1, no = 2),
               FUN = function(x){sum(x > 0)})

prevdf = data.frame(Prevalence = prevdf,
                    TotalAbundance = taxa_sums(ps0.dna),
                    tax_table(ps0.dna))

plyr::ddply(prevdf, "Phylum", function(df1){cbind(mean(df1$Prevalence),
                                                   sum(df1$Prevalence))})
```

```
##                      Phylum        1     2
## 1  Absconditabacteria_(SR1) 1.802198   164
## 2            Actinobacteria 1.818157 13098
## 3             Bacteroidetes 1.905040 19279
## 4                Chlamydiae 1.500000     3
## 5                  Chlorobi 3.000000     6
## 6               Chloroflexi 1.750000     7
## 7             Cyanobacteria 3.642857    51
```

```
## 8                Firmicutes 1.678160 36599
## 9              Fusobacteria 2.695513  4205
## 10   Gracilibacteria_(GN02) 2.400000    36
## 11            Proteobacteria 1.996799 18093
## 12   Saccharibacteria_(TM7) 2.613115   797
## 13              Spirochaetes 2.527559   321
## 14             Synergistetes 2.804878   115
```

```r
filterPhyla = c("Chloroflexi", "Chlorobi", "Chlamydiae")
ps1.dna = subset_taxa(ps0.dna, !Phylum %in% filterPhyla)
ps1.dna
```

```
## phyloseq-class experiment-level object
## otu_table()   OTU Table:         [ 50347 taxa and 79 samples ]
## sample_data() Sample Data:       [ 79 samples by 46 sample variables ]
## tax_table()   Taxonomy Table:    [ 50347 taxa by 7 taxonomic ranks ]
## refseq()      DNAStringSet:      [ 50347 reference sequences ]
```

```r
saveRDS(ps1.dna, file = "ps1.dna.rds")

ps1.dna.genus <- tax_glom(ps1.dna, "Genus", NArm = FALSE)

saveRDS(ps1.dna.genus, file = "ps1.dna.genus.rds")
```

A total of 15,301,705 reads were provided as initial input. After filtering, denoising, merging, chimera treatment and removal of non-target-length sequences, 6,276,040 sequences remained. After taxonomic assignment, a total of 51,738 ASVs were obtained. The ASVs unannotated at phyla level were removed as well as those that represent low prevalence phyla (Chlamydiae, Chlorobi and Chloroflexi). In total, 11 phyla, 200 genera, 364 species and 50,347 ASVs were identified.

0.2 Microbiome overview

0.2.1 Ajusting for calculations and tables

```r
ps1.dna <- readRDS("ps1.dna.rds")

ps.dna.phy <- tax_glom(ps1.dna, "Phylum", NArm = TRUE)
ps.dna.genus <- tax_glom(ps1.dna, "Genus", NArm = TRUE)
ps.dna.species <- tax_glom(ps1.dna, "Species", NArm = TRUE)

# ASV level
ps.ra <- transform_sample_counts(ps1.dna, function(x) x/sum(x))

# Phylum level
taxa_names(ps.dna.phy) <- tax_table(ps.dna.phy)[, 2]
ps.phy.ra <- transform_sample_counts(ps.dna.phy, function(x) x/sum(x))

# Genus level
ps.genus.ra <- transform_sample_counts(ps.dna.genus, function(x) x/sum(x))
genus.melt <- psmelt(ps.genus.ra)

# Species level
ps.species.ra <- transform_sample_counts(ps.dna.species, function(x) x/sum(x))
```

0.2.2 Dominant taxa and their overall relative abundance

0.2.2.1 Tables

The distribution of the reads are here summarized on phylum, genus, species and individual ASV level.

```r
library("rio")
library("kableExtra")

df2 <- data.frame(tax_table(ps.dna.phy),
                  taxprc = 100*taxa_sums(ps.phy.ra)/length(sample_names(ps.phy.ra)))
df3 <- data.frame(tax_table(ps.dna.genus),
                  taxprc = 100*taxa_sums(ps.genus.ra)/length(sample_names(ps.genus.ra)))
df4 <- data.frame(tax_table(ps.dna.species),
                  taxprc = 100*taxa_sums(ps.species.ra)/
                    length(sample_names(ps.species.ra)))
df5 <- data.frame(tax_table(ps1.dna),
                  taxprc = 100*taxa_sums(ps.ra)/length(sample_names(ps.ra)))

df.count <- data.frame(Included = c("All", "> 0.01%", "> 0.1%","> 1%"),
  Phylum = c(nrow(df2),sum(df2$taxprc > 0.01),sum(df2$taxprc > 0.1),sum(df2$taxprc > 1)),
  Genus = c(nrow(df3),sum(df3$taxprc > 0.01),sum(df3$taxprc > 0.1),sum(df3$taxprc > 1)),
  Species = c(nrow(df4),sum(df4$taxprc > 0.01),sum(df4$taxprc > 0.1),sum(df4$taxprc > 1)),
  ASV = c(nrow(df5),sum(df5$taxprc > 0.01),sum(df5$taxprc > 0.1),sum(df5$taxprc > 1)))

# Count of ASV and species in upper respiratory tract samples
kable(df.count, row.names = F,digits = 1,
  caption =
    'Table S1. Abundance of phyla, genera, species, and ASV in microbiome samples')%>%
  kable_classic(full_width = F, position = "left")
export(df.count,
      'Abundance of phyla, genera, species, and ASV in microbiome samples.xlsx')

# Top 6 dominating phyla (prc abundance)
kable(head(df2[order(df2$taxprc,decreasing = T),c("Kingdom","Phylum","taxprc")]),
  row.names = F,digits = 1, caption = 'Table S2. Average abundance according to phylum',
  col.names = c("Kingdom","Phylum","Abundance (%)"))%>%
  kable_classic(full_width = F, position = "left")
export(df2, 'Average abundance according to phylum.xlsx')

# Top 6 dominating (genera prc abundance)
kable(head(df3[order(df3$taxprc,decreasing = T),
  c("Kingdom","Phylum","Class","Order","Family","Genus","taxprc")]),
  row.names = F,digits = 1, caption = 'Table S3. Average abundance according to genus',
  col.names = c("Kingdom","Phylum","Class","Order","Family","Genus","Abundance (%)"))%>%
  kable_classic(full_width = F, position = "left")
export(df3, 'Average abundance according to genus.xlsx')

# Top 6 dominating species (prc abundance)
kable(head(df4[order(df4$taxprc,decreasing = T),
  c("Kingdom","Phylum","Class","Order","Family","Genus","Species","taxprc")]),
  row.names = F,digits = 1,
  caption = 'Table S4. Average abundance according to Species',
  col.names =
  c("Kingdom","Phylum","Class","Order","Family","Genus","Species","Abundance (%)"))%>%
```

```
  kable_classic(full_width = F, position = "left")
export(df4, 'Average abundance according to species.xlsx')

# clean environment
rm(list = ls(all = TRUE))
```

The data generated here are presented in Supplementary Material (Table 1 - Supplementary Data 2-5).

1.0 Alpha-diversity

1.1 Alpha-diversity analysis by Shannon diversity index

```
library(phyloseq)
library(ggpubr)
ps <- readRDS("ps1.dna.rds")

#M72 is a NC group outlier and should be removed
ps_nO <- subset_samples(ps, sampleID != "M72")

#Calculating for inspection
df.adiv <- cbind(data.frame(sample_data(ps)),
                 estimate_richness(ps, measures = c("Observed","Shannon")))

#Summary
df.adiv.summary <- df.adiv %>%
  group_by(COVID19) %>%
  summarise(n = n(), Observed_mean = mean(Observed), Observed_sd = sd(Observed),
            Shannon_mean = mean(Shannon), Shannon_sd = sd(Shannon))
export(df.adiv.summary, 'Alpha diversity summarised statistics.xlsx')

#Adjusting the agglomerated phyloseq object for further analyses
ps1.dna.genus <- readRDS("ps1.dna.genus.rds")
ps1.dna.genus_nO <- subset_samples(ps1.dna.genus, sampleID != "M72")

#Salvando objetos sem outliers
saveRDS(ps_nO, "ps_nO.rds")
saveRDS(ps1.dna.genus_nO, "ps1.dna.genus_nO.rds")

#Preparando o plot
comparisons <- list(c("M-CoV","NO-CoV"), c("M-CoV","S-CoV"), c("M-CoV","NC"),
                    c("NO-CoV","S-CoV"), c("NO-CoV","NC"), c("NC","S-CoV"))
palette <- c("#68228B","#7FFF00","#00bfff", "#FF521C")
groups <- c("M-CoV", "NO-CoV", "S-CoV", "NC")

alpha_div <- plot_richness(ps_nO, x="COVID19", color="COVID19", measures= "Shannon") +
  stat_compare_means(method="wilcox.test", comparisons = comparisons) + geom_boxplot() +
  theme_classic() + scale_color_manual(values = palette) +
  ylab("Shannon Index")

alpha_div$data$COVID19 <- factor(alpha_div$data$COVID19, levels = groups)

#Only statistically significant differences are flagged for better visualization.
alpha_div
```

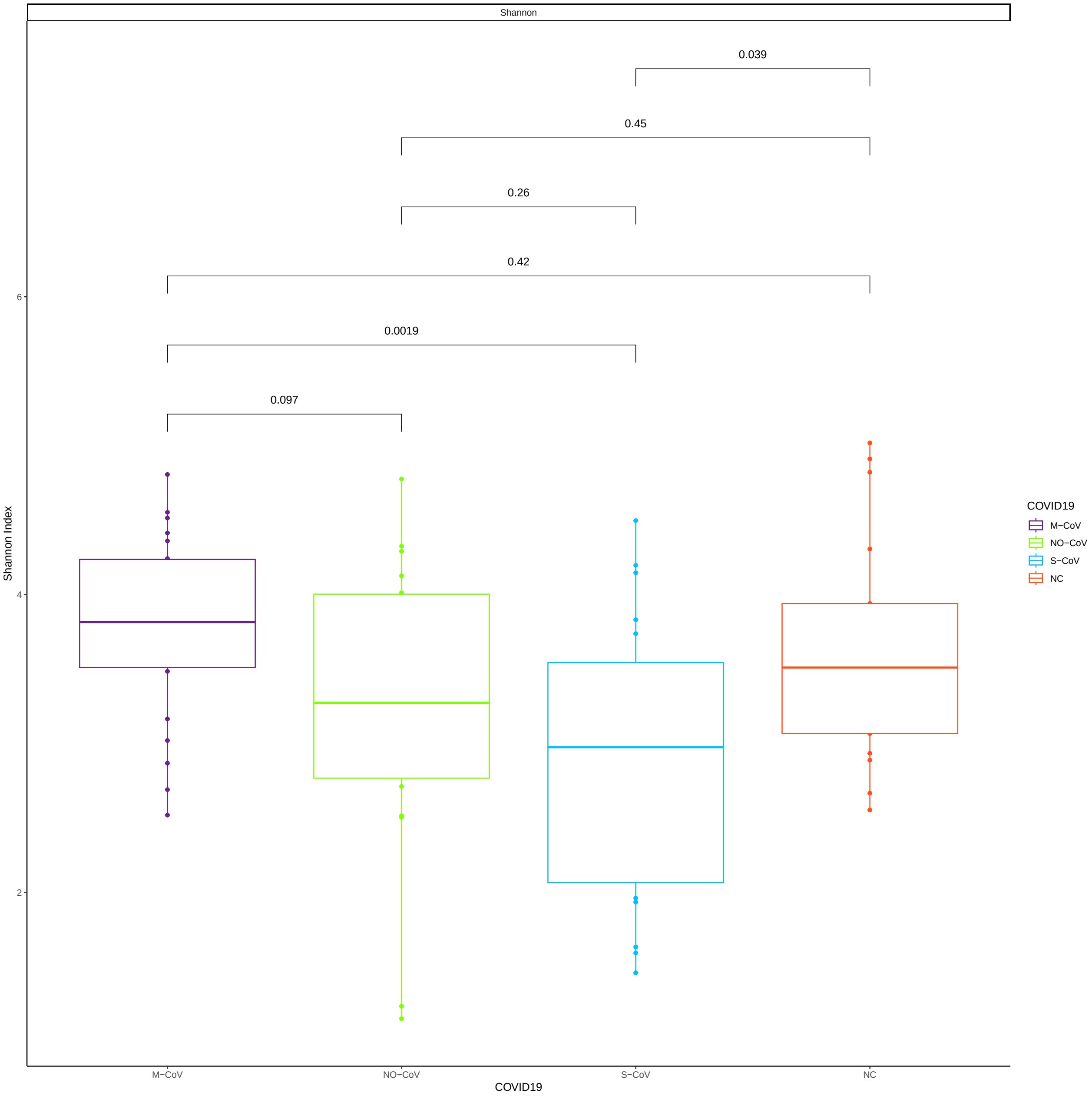

Figure 1: Boxplot of alpha diversity with Shannon diversity index presenting all p-values.

We removed M72, an NC group outlier. The alpha-diversity plot only with statistically significant p-values is presented in the main article.

2.0 Beta-diversity

2.1 Principal Component Analysis (PCA) with the Aitchison (Euclidean) distance

```
library(vegan)
library(ggrepel)
# Defining colors for plotting
palette <- c("#68228B","#7FFF00","#00bfff", "#FF521C")

ps <- readRDS("ps1.dna.genus_n0.rds")
table <- as.data.frame(otu_table(ps))

# Handling zeros
library(zCompositions)
table_no0 <- cmultRepl(table, output = "p-counts")
```

```
## No. adjusted imputations:  2158
```

```
# This function implements methods for imputing zeros in compositional count data
# sets based on a Bayesian-multiplicative replacement. These proportions are re-scaled
# to produce a compositionally-equivalent matrix of pseudo-counts (output="p-counts")
# which preserves the ratios between parts.

# Before carrying out the transformation, we must meet the compositionality requirements
table_no0 <- table_no0/apply(table_no0,1,sum)
head(rowSums(table_no0)) #All must be 1
```

```
## M03 M04 M05 M06 M07 M08
##   1   1   1   1   1   1
```

```
tail(rowSums(table_no0))
```

```
## M83 M84 M85 M86 M87 M88
##   1   1   1   1   1   1
```

```
#Saving for further analyses
saveRDS(table_no0, file = "table_no0.rds")

#Extracting metadata
meta_data <- microbiome::meta(ps)

#Saving for further analyses
saveRDS(meta_data, file = "meta_data.rds")

#CLR transformation (Centered Log Ratio)
library(easyCODA)
library(ecodist)
clr_data <- CLR(table_no0)

#Saving for further analyses
saveRDS(clr_data, file = "clr_data.rds")
```

```r
#Computing distances
clr_pca <- clr_data$LR
euclidean_dist <- vegan::vegdist(clr_pca, method = "euclidean")
euclidean_pca <- ecodist::pco(euclidean_dist)
euclidean_pca_df <- data.frame(pca1 = euclidean_pca$vectors[,1],
                               pca2 = euclidean_pca$vectors[,2])
euclidean_distances <- data.frame(euclidean_pca$values)
euclidean_sample_status_pca_df <- cbind(euclidean_pca_df,
                                        sample_status = meta_data$COVID19)
euclidean_sample_status_pca_df <- cbind(euclidean_sample_status_pca_df,
                                        sampleID = meta_data$sampleID)
#Making the plot
euclidean_sample_status_plot <- ggplot(data = euclidean_sample_status_pca_df,
                                       aes(x=pca1, y=pca2,
                                           color = sample_status)) +
  geom_point() +
  labs(x = "PC1",
       y = "PC2",
       title = "PCA with Aitchison distance") + stat_ellipse()+
  geom_text_repel(data = subset(euclidean_sample_status_pca_df, pca1 >0.0035),
  aes(pca1, pca2, label=sampleID)) + theme(title = element_text(size = 12)) +
  scale_color_manual(values = palette) + theme_classic()

groups <- c("M-CoV", "NO-CoV", "S-CoV", "NC")

euclidean_sample_status_plot$data$sample_status <-
  as.character(euclidean_sample_status_plot$data$sample_status)
euclidean_sample_status_plot$data$sample_status <-
  factor(euclidean_sample_status_plot$data$sample_status, levels = groups)

#Plot
euclidean_sample_status_plot
```

2.1.1 PERMANOVA for Aitchison distance

```r
adonis2(euclidean_dist ~ meta_data$Age + meta_data$Sex + meta_data$batch +
          meta_data$COVID19, data = meta_data, strata = meta_data$COVID19)
```

```
## Permutation test for adonis under reduced model
## Blocks:  strata
## Permutation: free
## Number of permutations: 999
##
## adonis2(formula = euclidean_dist ~ meta_data$Age + meta_data$Sex + meta_data$batch + meta_data$COVID
##          Df SumOfSqs      R2      F Pr(>F)
## Model     9    10768 0.17446 1.5967  0.056 .
## Residual 68    50953 0.82554
## Total    77    61721 1.00000
## ---
## Signif. codes:  0 '***' 0.001 '**' 0.01 '*' 0.05 '.' 0.1 ' ' 1
```

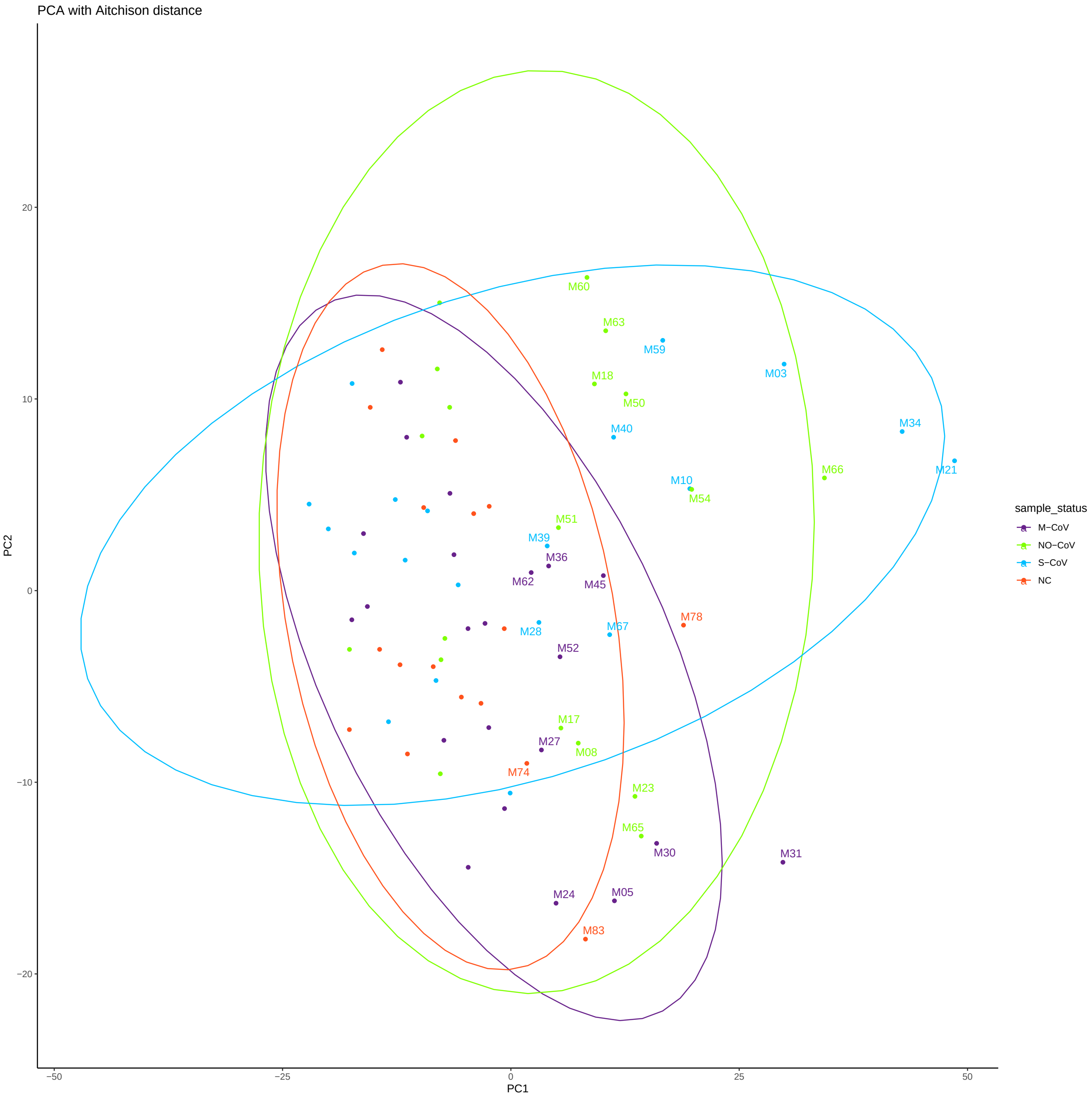

Figure 2: Principal Component Analysis (PCA) with the Aitchison distance

3.0 Differential Abundance

We used ANCOMBC2 for differential abundance analysis

Adjusting the data

```
ps <- readRDS("ps_n0.rds")
tse = mia::makeTreeSummarizedExperimentFromPhyloseq(ps)

# Note that by default, levels of a categorical variable in R are sorted alphabetically.
# The reference level must be the first.
tse$COVID19 = factor(tse$COVID19, levels = c("NC", "M-CoV", "NO-CoV", "S-CoV"))

# You can verify the change by checking:
levels(tse@colData@listData$COVID19)

print(tse)
```

We decided to use NC group as the reference level.

Run ANCOMBC2

```
set.seed(123)

output = ancombc2(data = tse, assay_name = "counts", tax_level = "Genus",
                  fix_formula = "batch + Age + Sex + COVID19", rand_formula = NULL,
                  p_adj_method = "BH",
                  prv_cut = 0.10, lib_cut = 1000, s0_perc = 0.05,
                  group = "COVID19", struc_zero = TRUE, neg_lb = TRUE,
                  alpha = 0.05, n_cl = 8, verbose = TRUE,
                  global = TRUE, pairwise = TRUE, dunnet = FALSE, trend = FALSE,
                  iter_control = list(tol = 1e-2, max_iter = 20,
                                      verbose = TRUE),
                  em_control = list(tol = 1e-5, max_iter = 100),
                  lme_control = lme4::lmerControl(),
                  mdfdr_control = list(fwer_ctrl_method = "holm", B = 100))

save(file = 'ANCOMBC.RData',list = "output")

# clean environment
rm(list = ls(all = TRUE))
```

3.1 Structural zeros on genus level

```
load('ANCOMBC_global.RData')
library("tidyr")
library("rio")
library("DT")
tab_zero = output$zero_ind

export(tab_zero, "ANCOMBC2_structural_zeros.xlsx")
```

The structural zeros are presented in Supplementary information - Supplementary data 8.

Some structural zeros were detected in our experimental groups. Of note, Achromobacter sp., Absconditabacteria_(SR1)_[G-1], Bacillus sp., order Corynebacteriales, family Propionibacteriaceae, order Sphingomonadales and family Prevotellaceae were structural zeros in S-CoV.

3.2 ANCOM-BC2 Global test

```
res_prim = output$res
res_global = output$res_global
export(res_prim, "ANCOMBC2_primary_analysis.xlsx")
export(res_global, "ANCOMBC2_global_test.xlsx")
```

Global test Plot

```
df_COVID = res_prim %>%
    dplyr::select(taxon, contains("COVID19"))
df_fig_global = df_COVID %>%
    dplyr::left_join(res_global %>%
                       dplyr::transmute(taxon,
                                        diff_COVID19 = diff_abn,
                                        passed_ss = passed_ss)) %>%
    dplyr::filter(diff_COVID19 == TRUE) %>%
    dplyr::mutate(`lfc_M-CoV` = `lfc_COVID19M-CoV`,
                  `lfc_NO-CoV` = `lfc_COVID19NO-CoV`,
                  `lfc_S-CoV` = `lfc_COVID19S-CoV`) %>%
    dplyr::transmute(taxon,
                     `M-CoV - NC` = round(`lfc_M-CoV`, 2),
                     `NO-CoV - NC` = round(`lfc_NO-CoV`, 2),
                     `S-CoV - NC` = round(`lfc_S-CoV`, 2)) %>%
    tidyr::pivot_longer(cols = `M-CoV - NC`:`NO-CoV - NC`:`S-CoV - NC`,
                        names_to = "group", values_to = "value") %>%
    dplyr::arrange(taxon)

df_fig_global$group = factor(df_fig_global$group,
                             levels = c("M-CoV - NC",
                                        "NO-CoV - NC",
                                        "S-CoV - NC"))

df_fig_global$taxon <- sub("Genus:", "", df_fig_global$taxon)
df_fig_global$group <- as.character(df_fig_global$group)

#Plot
fig_global = df_fig_global %>%
  ggplot(aes(x = taxon, y = value,
  fill = ifelse(value >= 0, "Positive LFC", "Negative LFC"))) +
  geom_bar(stat = "identity", width = 0.7, color = "black",
  position = position_dodge(width = 0.4)) +
  labs(x = "Taxon", y = "Log fold change",
  title = "Log fold change of globally significant taxa") +
  scale_fill_manual(values = c("Positive LFC" = "blue", "Negative LFC" = "red"),
  name = "Log fold change") +
  facet_wrap(~group, nrow = 3) +
  theme_bw() +
  theme(plot.title = element_text(hjust = 0.5),
  axis.text.x = element_text(angle = 45, hjust = 1, face = "italic"))

ggsave("Fig 2 ANCOMBC2 Global test.pdf", plot = fig_global, width = 180, height = 170,
       units = "mm", dpi = 600)
```

The global test plot is presented in the article.

3.3 ANCOM-BC2 multiple pairwise comparisons on genus level

```
res_pair = output$res_pair
export(res_pair, "ANCOMBC2_pairwise_comparisons.xlsx")
```

Pairwise comparisons plot

```
df_fig_pair = res_pair %>%
  filter(`diff_COVID19Group 2` == TRUE & `passed_ss_COVID19Group 2` == TRUE |
  `diff_COVID19Group 3` == TRUE & `passed_ss_COVID19Group 3` == TRUE |
  `diff_COVID19Group 4` == TRUE & `passed_ss_COVID19Group 4` == TRUE |
  `diff_COVID19Group 3_COVID19Group 2` & `passed_ss_COVID19Group 3_COVID19Group 2` |
  `diff_COVID19Group 4_COVID19Group 2` & `passed_ss_COVID19Group 4_COVID19Group 2` |
  `diff_COVID19Group 4_COVID19Group 3` & `passed_ss_COVID19Group 4_COVID19Group 3`) %>%
  mutate(`lfc_Group 2` = ifelse(`diff_COVID19Group 2` == TRUE,
                                 `lfc_COVID19Group 2`, 0),
         `lfc_Group 3` = ifelse(`diff_COVID19Group 3` == TRUE,
                                 `lfc_COVID19Group 3`, 0),
         `lfc_Group 4` = ifelse(`diff_COVID19Group 4` == TRUE,
                                 -1 * `lfc_COVID19Group 4`, 0),
         `lfc_Group 3_Group 2` = ifelse(`diff_COVID19Group 3_COVID19Group 2` == TRUE,
                                 `lfc_COVID19Group 3_COVID19Group 2`, 0),
         `lfc_Group 4_Group 2` = ifelse(`diff_COVID19Group 4_COVID19Group 2` == TRUE,
                                 -1 * `lfc_COVID19Group 4_COVID19Group 2`, 0),
         `lfc_Group 4_Group 3` = ifelse(`diff_COVID19Group 4_COVID19Group 3` == TRUE,
                                 -1 * `lfc_COVID19Group 4_COVID19Group 3`, 0)) %>%
    transmute(taxon,
              `Group 2 - Group 1` = round(`lfc_Group 2`, 2),
              `Group 3 - Group 1` = round(`lfc_Group 3`, 2),
              `Group 1 - Group 4` = round(`lfc_Group 4`, 2),
              `Group 3 - Group 2` = round(`lfc_Group 3_Group 2`, 2),
              `Group 2 - Group 4` = round(`lfc_Group 4_Group 2`, 2),
              `Group 3 - Group 4` = round(`lfc_Group 4_Group 3`, 2)) %>%
    pivot_longer(cols = `Group 2 - Group 1`:`Group 3 - Group 1`:`Group 1 - Group 4`:
                  `Group 3 - Group 2`:`Group 2 - Group 4`:`Group 3 - Group 4`,
                  names_to = "group", values_to = "value") %>%
                  arrange(taxon)

df_fig_pair$taxon <- sub("Genus:", "", df_fig_pair$taxon)
df_fig_pair$group <- as.character(df_fig_pair$group)

df_fig_pair$group[df_fig_pair$group == "Group 2 - Group 1"] <- "NO-CoV - M-CoV"
df_fig_pair$group[df_fig_pair$group == "Group 3 - Group 1"] <- "S-CoV - M-CoV"
df_fig_pair$group[df_fig_pair$group == "Group 1 - Group 4"] <- "M-CoV - NC"
df_fig_pair$group[df_fig_pair$group == "Group 3 - Group 2"] <- "S-CoV - NO-CoV"
df_fig_pair$group[df_fig_pair$group == "Group 2 - Group 4"] <- "NO-CoV - NC"
df_fig_pair$group[df_fig_pair$group == "Group 3 - Group 4"] <- "S-CoV - NC"

df_fig_pair$group = factor(df_fig_pair$group,
                           levels = c("M-CoV - NC",
                                      "NO-CoV - NC",
                                      "S-CoV - NC",
                                      "NO-CoV - M-CoV",
                                      "S-CoV - NO-CoV",
```

```
                                              "S-CoV - M-CoV"))

fig_pair = df_fig_pair %>%
  ggplot(aes(x = taxon, y = value,
  fill = ifelse(value >= 0, "Positive LFC", "Negative LFC"))) +
  geom_bar(stat = "identity", width = 0.7, color = "black",
  position = position_dodge(width = 0.4)) +
  labs(x = "Taxon", y = "Log fold change",
  title = "Log fold change of pairwise comparisons") +
  scale_fill_manual(values = c("Positive LFC" = "blue", "Negative LFC" = "red"),
  name = "Log fold change") +
  facet_wrap(~group, nrow = 2) +
  theme_bw() +
  theme(plot.title = element_text(hjust = 0.5),
  axis.text.y = element_text(angle = 0, hjust = 1, face = "italic", size = 7)) +
  coord_flip()

ggsave("Fig 3 ANCOMBC2 Pairwise Comparisons.pdf", plot = fig_pair, width = 180,
       height = 170, units = "mm", dpi = 600)
```

The differential abundance pairwise comparisons plot is presented in the article.

4.0 Functional and metabolic prediction with PICRUSt2 (Phylogenetic Investigation of Communities by Reconstruction of Unobserved States)

4.1 Exporting the data for PICRUSt2

```
# Exporting the data for PICRUSt2
library(phyloseq)
library(Biostrings)

ps <- readRDS("ps_n0.rds")

# Exporting sequences fasta
sequences <- refseq(ps)
sequences_char <- as.character(sequences)
fasta_headers <- paste(">", names(sequences), sep = "")
fasta_content <- paste(fasta_headers, sequences_char, sep = "\n")
writeLines(fasta_content, "sequences.fasta")

# Exportar feature table Biom format file
library(biomformat)
packageVersion("biomformat")
otu <- t(as(otu_table(ps), "matrix"))  # 't' if taxa_are_rows=FALSE
otu_biom <- make_biom(data = otu)
write_biom(otu_biom, "otu_biom.biom")

# Export metadata
sam_data <- as.matrix(sample_data(ps))
write.table(sam_data, "sample_metadata.txt", sep = "\t", row.names = FALSE, col.names = TRUE,
    quote = FALSE)
```

```r
# clean environment
rm(list = ls(all = TRUE))
```

4.2 Metabolic prediction - MetaCyc Pathways

```r
#Metabolic prediction - MetaCyc Pathways
library(phyloseq)
library(readr)
library(ggpicrust2)
library(tibble)
library(tidyverse)
library(ggprism)
library(patchwork)
library(dplyr)
library(rio)
abundance_file <-
 "/home/metagenomica/LABRESIS/OtavioLovison/Paper/PICRUSt2/picrust2_out_pipeline/
pathways_out/path_abun_unstrat.tsv"
metadata <- read_delim(
    "/home/metagenomica/LABRESIS/OtavioLovison/Paper/PICRUSt2/sample_metadata.txt",
    delim = "\t",
    escape_double = FALSE,
    trim_ws = TRUE
)

metacyc_abundance <- read_delim(abundance_file, delim = "\t", col_names = TRUE,
                                trim_ws = TRUE)

#DAA1
abundance <- metacyc_abundance %>% column_to_rownames("pathway")
group <- "COVID19"
reference <- "Group 1"
formula <- "~Age + Sex + batch + Group_group_nonsense_"
p.adjust <- "BH"

if (!tibble::is_tibble(metadata)) {
        message("Converting metadata to tibble...")
        metadata <- tibble::as_tibble(metadata)
    }
    sample_names <- colnames(abundance)
    message("Sample names extracted.")
    message("Identifying matching columns in metadata...")
    matches <- base::lapply(metadata, function(x) {
        intersect(sample_names, x)
    })
    matching_columns <- names(metadata)[sapply(matches, function(x) {
        length(x) == length(sample_names)
    })][1]
    if (!is.null(matching_columns)) {
        message(paste("Matching columns identified:", matching_columns,
            ". This is important for ensuring data consistency."))
    }
```

```
  switch(is.null(select), `TRUE` = {
      message("Using all columns in abundance.")
  }, `FALSE` = {
      message("Filtering the abundance and metadata...")
      abundance <- abundance[, colnames(abundance) %in% select]
      metadata <- metadata[as.matrix(metadata[, matching_columns]) %in%
          select, ]
  })
  sample_names <- colnames(abundance)
  message("Converting abundance to a matrix...")
  abundance_mat <- as.matrix(abundance)
  message("Reordering metadata...")
  metadata_order <- match(sample_names, as.matrix(metadata[,
      matching_columns]))
  metadata <- metadata[metadata_order, ]
  message("Converting metadata to a matrix and data frame...")
  metadata_mat <- as.matrix(metadata)
  metadata_df <- as.data.frame(metadata)
  message("Extracting group information...")
  Group <- factor(metadata_mat[, group])
  Level <- levels(Group)
  length_Level <- length(Level)

LinDA_metadata_df <- metadata_df
LinDA_colnames <- colnames(LinDA_metadata_df)
LinDA_colnames[LinDA_colnames == group] <- "Group_group_nonsense_"
colnames(LinDA_metadata_df) <- LinDA_colnames
rownames(LinDA_metadata_df) <- LinDA_metadata_df[, matching_columns]
LinDA_metadata_df <- dplyr::select(LinDA_metadata_df, -matching_columns)
LinDA_metadata_df$Group_group_nonsense_ <-
  factor(LinDA_metadata_df$Group_group_nonsense_)
      if (length_Level != 2) {
          if (is.null(reference)) {
              stop("Error: A reference group is required when using LinDA or
                   limma voom for comparisons among more than two groups.
                   Please specify a reference group.")
          }
          LinDA_metadata_df$Group_group_nonsense_ <-
            stats::relevel(LinDA_metadata_df$Group_group_nonsense_,
              ref = reference)
      }
      message("Performing LinDA analysis...")
      LinDA_results <- MicrobiomeStat::linda(abundance, LinDA_metadata_df,
          formula = formula, alpha = 0.05)$output
      message("Processing LinDA results...")
      length(LinDA_results)
      if (length_Level != 2) {
          for (i in 1:length(LinDA_results)) {
              LinDA_results[[i]] <- cbind(feature = rownames(LinDA_results[[i]]),
                method = "LinDA", group1 = substr(names(LinDA_results)[i],
                  22, stop = nchar(names(LinDA_results)[i])),
                group2 = reference, p_values = LinDA_results[[i]]$pvalue)
          }
```

```r
        } else {
            for (i in 1:length(LinDA_results)) {
                LinDA_results[[i]] <- cbind(feature = rownames(LinDA_results[[i]]),
                  method = "LinDA", group1 = Level[1], group2 = Level[2],
                  p_values = LinDA_results[[i]]$pvalue)
            }
        }
        message("LinDA analysis is complete.")
        LinDA_results <- LinDA_results[7:length(LinDA_results)]
        p_values_matrix <- as.matrix(do.call(rbind, LinDA_results))
        p_values_df <- as.data.frame(p_values_matrix)

        valid_p_adjust <- c("BH", "holm", "bonferroni", "hochberg",
        "fdr", "none")
    if (!p.adjust %in% valid_p_adjust) {
        stop(paste("Invalid p.adjust method. Please choose from:",
            paste(valid_p_adjust, collapse = ", ")))
    }
    if (!exists("p_values_df") || nrow(p_values_df) == 0) {
        stop("Notice: There are no statistical significances detected.
             This is not an error, but it might indicate that your data does not
             contain any values passing the set significance threshold (p<=0.05).
             You may refer to the tutorial's FAQ for further help and suggestions.")
    }
    switch(p.adjust, BH = {
        adjusted_p_values <- p.adjust(p_values_df$p_values, method = "BH")
    }, holm = {
        adjusted_p_values <- p.adjust(p_values_df$p_values, method = "holm")
    }, bonferroni = {
        adjusted_p_values <- p.adjust(p_values_df$p_values, method = "bonferroni")
    }, hochberg = {
        adjusted_p_values <- p.adjust(p_values_df$p_values, method = "hochberg")
    }, fdr = {
        adjusted_p_values <- p.adjust(p_values_df$p_values, method = "fdr")
    }, none = {
        adjusted_p_values <- p.adjust(p_values_df$p_values, method = "none")
    })
    daa_results_df1 <- cbind(p_values_df, adj_method = p.adjust,
        p_adjust = adjusted_p_values)

##################################################################################
#DAA2
abundance <- metacyc_abundance %>% column_to_rownames("pathway")
group <- "COVID19"
reference <- "Group 2"
formula <- "~Age + Sex + batch + Group_group_nonsense_"
p.adjust <- "BH"

if (!tibble::is_tibble(metadata)) {
        message("Converting metadata to tibble...")
        metadata <- tibble::as_tibble(metadata)
    }
    sample_names <- colnames(abundance)
```

```
  message("Sample names extracted.")
  message("Identifying matching columns in metadata...")
  matches <- base::lapply(metadata, function(x) {
      intersect(sample_names, x)
  })
  matching_columns <- names(metadata)[sapply(matches, function(x) {
      length(x) == length(sample_names)
  })][1]
  if (!is.null(matching_columns)) {
      message(paste("Matching columns identified:", matching_columns,
          ". This is important for ensuring data consistency."))
  }

  switch(is.null(select), `TRUE` = {
      message("Using all columns in abundance.")
  }, `FALSE` = {
      message("Filtering the abundance and metadata...")
      abundance <- abundance[, colnames(abundance) %in% select]
      metadata <- metadata[as.matrix(metadata[, matching_columns]) %in%
          select, ]
  })
  sample_names <- colnames(abundance)
  message("Converting abundance to a matrix...")
  abundance_mat <- as.matrix(abundance)
  message("Reordering metadata...")
  metadata_order <- match(sample_names, as.matrix(metadata[,
      matching_columns]))
  metadata <- metadata[metadata_order, ]
  message("Converting metadata to a matrix and data frame...")
  metadata_mat <- as.matrix(metadata)
  metadata_df <- as.data.frame(metadata)
  message("Extracting group information...")
  Group <- factor(metadata_mat[, group])
  Level <- levels(Group)
  length_Level <- length(Level)

LinDA_metadata_df <- metadata_df
LinDA_colnames <- colnames(LinDA_metadata_df)
LinDA_colnames[LinDA_colnames == group] <- "Group_group_nonsense_"
colnames(LinDA_metadata_df) <- LinDA_colnames
rownames(LinDA_metadata_df) <- LinDA_metadata_df[, matching_columns]
LinDA_metadata_df <- dplyr::select(LinDA_metadata_df,
          -matching_columns)
LinDA_metadata_df$Group_group_nonsense_ <-
  factor(LinDA_metadata_df$Group_group_nonsense_)
    if (length_Level != 2) {
        if (is.null(reference)) {
            stop("Error: A reference group is required when using LinDA or
limma voom for comparisons among more than two groups.
Please specify a reference group.")
          }
          LinDA_metadata_df$Group_group_nonsense_ <-
            stats::relevel(LinDA_metadata_df$Group_group_nonsense_,
```

```r
                ref = reference)
        }
        message("Performing LinDA analysis...")
        LinDA_results <- MicrobiomeStat::linda(abundance, LinDA_metadata_df,
            formula = formula, alpha = 0.05)$output
        message("Processing LinDA results...")
        length(LinDA_results)
        if (length_Level != 2) {
            for (i in 1:length(LinDA_results)) {
                LinDA_results[[i]] <- cbind(feature = rownames(LinDA_results[[i]]),
                  method = "LinDA", group1 = substr(names(LinDA_results)[i],
                    22, stop = nchar(names(LinDA_results)[i])),
                  group2 = reference, p_values = LinDA_results[[i]]$pvalue)
            }
        } else {
            for (i in 1:length(LinDA_results)) {
                LinDA_results[[i]] <- cbind(feature = rownames(LinDA_results[[i]]),
                  method = "LinDA", group1 = Level[1], group2 = Level[2],
                  p_values = LinDA_results[[i]]$pvalue)
            }
        }
        message("LinDA analysis is complete.")
        LinDA_results <- LinDA_results[7:length(LinDA_results)]
        p_values_matrix <- as.matrix(do.call(rbind, LinDA_results))
        p_values_df <- as.data.frame(p_values_matrix)

        valid_p_adjust <- c("BH", "holm", "bonferroni", "hochberg",
        "fdr", "none")
    if (!p.adjust %in% valid_p_adjust) {
        stop(paste("Invalid p.adjust method. Please choose from:",
            paste(valid_p_adjust, collapse = ", ")))
    }
    if (!exists("p_values_df") || nrow(p_values_df) == 0) {
      stop("Notice: There are no statistical significances detected.
        This is not an error, but it might indicate that your data does not contain
             any values passing the set significance threshold (p<=0.05).
             You may refer to the tutorial's FAQ for further help and suggestions.")
    }
    switch(p.adjust, BH = {
        adjusted_p_values <- p.adjust(p_values_df$p_values, method = "BH")
    }, holm = {
        adjusted_p_values <- p.adjust(p_values_df$p_values, method = "holm")
    }, bonferroni = {
        adjusted_p_values <- p.adjust(p_values_df$p_values, method = "bonferroni")
    }, hochberg = {
        adjusted_p_values <- p.adjust(p_values_df$p_values, method = "hochberg")
    }, fdr = {
        adjusted_p_values <- p.adjust(p_values_df$p_values, method = "fdr")
    }, none = {
        adjusted_p_values <- p.adjust(p_values_df$p_values, method = "none")
    })
    daa_results_df2 <- cbind(p_values_df, adj_method = p.adjust,
        p_adjust = adjusted_p_values)
```

```r
###################################################################################
#DAA3
abundance <- metacyc_abundance %>% column_to_rownames("pathway")
group <- "COVID19"
reference <- "Group 3"
formula <- "~Age + Sex + batch + Group_group_nonsense_"
p.adjust <- "BH"

if (!tibble::is_tibble(metadata)) {
        message("Converting metadata to tibble...")
        metadata <- tibble::as_tibble(metadata)
    }
    sample_names <- colnames(abundance)
    message("Sample names extracted.")
    message("Identifying matching columns in metadata...")
    matches <- base::lapply(metadata, function(x) {
        intersect(sample_names, x)
    })
    matching_columns <- names(metadata)[sapply(matches, function(x) {
        length(x) == length(sample_names)
    })][1]
    if (!is.null(matching_columns)) {
        message(paste("Matching columns identified:", matching_columns,
            ". This is important for ensuring data consistency."))
    }

    switch(is.null(select), `TRUE` = {
        message("Using all columns in abundance.")
    }, `FALSE` = {
        message("Filtering the abundance and metadata...")
        abundance <- abundance[, colnames(abundance) %in% select]
        metadata <- metadata[as.matrix(metadata[, matching_columns]) %in%
            select, ]
    })
    sample_names <- colnames(abundance)
    message("Converting abundance to a matrix...")
    abundance_mat <- as.matrix(abundance)
    message("Reordering metadata...")
    metadata_order <- match(sample_names, as.matrix(metadata[,
        matching_columns]))
    metadata <- metadata[metadata_order, ]
    message("Converting metadata to a matrix and data frame...")
    metadata_mat <- as.matrix(metadata)
    metadata_df <- as.data.frame(metadata)
    message("Extracting group information...")
    Group <- factor(metadata_mat[, group])
    Level <- levels(Group)
    length_Level <- length(Level)

        LinDA_metadata_df <- metadata_df
        LinDA_colnames <- colnames(LinDA_metadata_df)
        LinDA_colnames[LinDA_colnames == group] <- "Group_group_nonsense_"
        colnames(LinDA_metadata_df) <- LinDA_colnames
```

```
    rownames(LinDA_metadata_df) <- LinDA_metadata_df[, matching_columns]
    LinDA_metadata_df <- dplyr::select(LinDA_metadata_df,
        -matching_columns)
    LinDA_metadata_df$Group_group_nonsense_ <-
      factor(LinDA_metadata_df$Group_group_nonsense_)
    if (length_Level != 2) {
        if (is.null(reference)) {
            stop("Error: A reference group is required when using LinDA or
                 limma voom for comparisons among more than two groups.
                 Please specify a reference group.")
        }
        LinDA_metadata_df$Group_group_nonsense_ <-
          stats::relevel(LinDA_metadata_df$Group_group_nonsense_,
            ref = reference)
    }
    message("Performing LinDA analysis...")
    LinDA_results <- MicrobiomeStat::linda(abundance, LinDA_metadata_df,
        formula = formula, alpha = 0.05)$output
    message("Processing LinDA results...")
    length(LinDA_results)
    if (length_Level != 2) {
        for (i in 1:length(LinDA_results)) {
            LinDA_results[[i]] <- cbind(feature = rownames(LinDA_results[[i]]),
              method = "LinDA", group1 = substr(names(LinDA_results)[i],
                22, stop = nchar(names(LinDA_results)[i])),
              group2 = reference, p_values = LinDA_results[[i]]$pvalue)
        }
    } else {
        for (i in 1:length(LinDA_results)) {
            LinDA_results[[i]] <- cbind(feature = rownames(LinDA_results[[i]]),
              method = "LinDA", group1 = Level[1], group2 = Level[2],
              p_values = LinDA_results[[i]]$pvalue)
        }
    }
    message("LinDA analysis is complete.")
    LinDA_results <- LinDA_results[7:length(LinDA_results)]
    p_values_matrix <- as.matrix(do.call(rbind, LinDA_results))
    p_values_df <- as.data.frame(p_values_matrix)

    valid_p_adjust <- c("BH", "holm", "bonferroni", "hochberg",
    "fdr", "none")
if (!p.adjust %in% valid_p_adjust) {
    stop(paste("Invalid p.adjust method. Please choose from:",
        paste(valid_p_adjust, collapse = ", ")))
}
if (!exists("p_values_df") || nrow(p_values_df) == 0) {
  stop("Notice: There are no statistical significances detected.
    This is not an error, but it might indicate that your data does not contain
    any values passing the set significance threshold (p<=0.05).
    You may refer to the tutorial's FAQ for further help and suggestions.")
}
switch(p.adjust, BH = {
    adjusted_p_values <- p.adjust(p_values_df$p_values, method = "BH")
```

```r
    }, holm = {
        adjusted_p_values <- p.adjust(p_values_df$p_values, method = "holm")
    }, bonferroni = {
        adjusted_p_values <- p.adjust(p_values_df$p_values, method = "bonferroni")
    }, hochberg = {
        adjusted_p_values <- p.adjust(p_values_df$p_values, method = "hochberg")
    }, fdr = {
        adjusted_p_values <- p.adjust(p_values_df$p_values, method = "fdr")
    }, none = {
        adjusted_p_values <- p.adjust(p_values_df$p_values, method = "none")
    })
    daa_results_df3 <- cbind(p_values_df, adj_method = p.adjust,
        p_adjust = adjusted_p_values)

################################################################################

# Annotation
metacyc_daa_annotated_results_df1 <- pathway_annotation(pathway = "MetaCyc",
                                    daa_results_df = daa_results_df1, ko_to_kegg = FALSE)
metacyc_daa_annotated_results_df2 <- pathway_annotation(pathway = "MetaCyc",
                                    daa_results_df = daa_results_df2, ko_to_kegg = FALSE)
metacyc_daa_annotated_results_df3 <- pathway_annotation(pathway = "MetaCyc",
                                    daa_results_df = daa_results_df3, ko_to_kegg = FALSE)

#Filtering duplicates
filtered_df2 <- metacyc_daa_annotated_results_df2 %>%
  filter(group1 != "Group 1")

filtered_df3 <- metacyc_daa_annotated_results_df3 %>%
  filter(group1 != "Group 1" & group1 != "Group 2")

#Concatenating
metacyc_daa_all_results_df <-
  rbind(metacyc_daa_annotated_results_df1, filtered_df2, filtered_df3)

#Adjusting variable names for plotting
metacyc_daa_all_results_df$group1[metacyc_daa_all_results_df$group1 == "Group 2"] <-
  "NO-CoV"
metacyc_daa_all_results_df$group1[metacyc_daa_all_results_df$group1 == "Group 3"] <-
  "S-CoV"
metacyc_daa_all_results_df$group1[metacyc_daa_all_results_df$group1 == "Group 4"] <-
  "NC"
metacyc_daa_all_results_df$group2[metacyc_daa_all_results_df$group2 == "Group 1"] <-
  "M-CoV"
metacyc_daa_all_results_df$group2[metacyc_daa_all_results_df$group2 == "Group 2"] <-
  "NO-CoV"
metacyc_daa_all_results_df$group2[metacyc_daa_all_results_df$group2 == "Group 3"] <-
  "S-CoV"
metadata$COVID19[metadata$COVID19 == "Group 1"] <- "M-CoV"
metadata$COVID19[metadata$COVID19 == "Group 2"] <- "NO-CoV"
metadata$COVID19[metadata$COVID19 == "Group 3"] <- "S-CoV"
metadata$COVID19[metadata$COVID19 == "Group 4"] <- "NC"
```

```r
# Making the plot
abundance = metacyc_abundance %>% column_to_rownames("pathway")
daa_results_df = metacyc_daa_all_results_df
Group = metadata$COVID19
p_values_threshold = 0.05
order = "group"
select = NULL
ko_to_kegg = FALSE
p_value_bar = TRUE
colors = c("M-CoV"="#68228B","NO-CoV"="#7FFF00","S-CoV"="#00bfff","NC"="#FF521C")
x_lab = "description"

# Identifying missing pathways
missing_pathways <- daa_results_df[is.na(daa_results_df$pathway_name), "feature"]

# Extracting colnames
column_names <- colnames(daa_results_df)

# Excluding missing annotations
daa_results_df <- daa_results_df[!is.na(daa_results_df[,x_lab]),]

#Assigning abundance matrix
errorbar_abundance_mat <- as.matrix(abundance)

#Filtering statistically significant results
daa_results_filtered_df <-
      daa_results_df[daa_results_df$p_adjust < p_values_threshold,]

if (!is.null(select)) {
  daa_results_filtered_sub_df <-
        daa_results_filtered_df[daa_results_filtered_df$feature %in% select, ]
    } else {
      daa_results_filtered_sub_df <- daa_results_filtered_df
    }

    if (nrow(daa_results_filtered_sub_df) > 30) {
      message(
        paste0(
    "The number of features with statistical significance exceeds 30,
    leading to suboptimal visualization. ",
    "Please use 'select' to reduce the number of features.\n",
    "Currently, you have these features: ",
    paste(paste0('"', daa_results_filtered_sub_df$feature, '"'), collapse = ", "), ".\n",
    "You can find the statistically significant features with the following command:\n",
    "daa_results_df %>% filter(p_adjust < 0.05) %>% select(c(\"feature\",\"p_adjust\"))"
        )
      )
      # stop()
    }

    if (nrow(daa_results_filtered_sub_df) == 0){
      stop(
        "Visualization with 'pathway_errorbar' cannot be performed because there are no
```

```r
        features with statistical significance. ",
        "For possible solutions, please check the FAQ section of the tutorial."
      )
    }

# Convert to relative abundance
relative_abundance_mat <- apply(t(errorbar_abundance_mat), 1, function(x)
  x / sum(x))

# Subset to only include the features present in daa_results_filtered_sub_df$feature
sub_relative_abundance_mat <-
  relative_abundance_mat[rownames(relative_abundance_mat) %in%
                         daa_results_filtered_sub_df$feature,]

# Create a matrix for the error bars
error_bar_matrix <- cbind(
      sample = colnames(sub_relative_abundance_mat),
      group = Group,
      t(sub_relative_abundance_mat)
    )

error_bar_df <- as.data.frame(error_bar_matrix)
error_bar_df$group <- factor(Group, levels = levels(as.factor(Group)))
error_bar_pivot_longer_df <- tidyr::pivot_longer(error_bar_df,-c(sample, group))
error_bar_pivot_longer_tibble <-
  mutate(error_bar_pivot_longer_df, group = as.factor(group))
error_bar_pivot_longer_tibble$sample <- factor(error_bar_pivot_longer_tibble$sample)
error_bar_pivot_longer_tibble$name <- factor(error_bar_pivot_longer_tibble$name)
error_bar_pivot_longer_tibble$value <- as.numeric(error_bar_pivot_longer_tibble$value)

error_bar_pivot_longer_tibble_summarised <- error_bar_pivot_longer_tibble %>%
  group_by(name, group) %>%
  summarise(mean = mean(value), sd = stats::sd(value))
#error_bar_pivot_longer_tibble_summarised <-
#error_bar_pivot_longer_tibble_summarised %>% mutate(group2 = "nonsense")
 switch(
 order,
 "p_values" = {
 #order <- order(daa_results_filtered_sub_df$p_adjust)
 order <- order(daa_results_filtered_sub_df$feature)
   },
 "name" = {
 order <- order(daa_results_filtered_sub_df$feature)
   },
 "group" = {
 daa_results_filtered_sub_df$pro <- 1
 for (i in levels(error_bar_pivot_longer_tibble_summarised$name)) {
 error_bar_pivot_longer_tibble_summarised_sub <-
 error_bar_pivot_longer_tibble_summarised[error_bar_pivot_longer_tibble_summarised$name ==
                                                      i,]
 pro_group <-
 error_bar_pivot_longer_tibble_summarised_sub
 [error_bar_pivot_longer_tibble_summarised_sub$mean ==
```

```
 max(error_bar_pivot_longer_tibble_summarised_sub$mean),]$group
 pro_group <- as.vector(pro_group)
 daa_results_filtered_sub_df[daa_results_filtered_sub_df$feature ==
                                  i,]$pro <- pro_group
    }
 order <-
 order(daa_results_filtered_sub_df$pro,
       daa_results_filtered_sub_df$p_adjust)
   },
  "pathway_class" = {
   if (!"pathway_class" %in% colnames(daa_results_filtered_sub_df)) {
  stop(
  "The 'pathway_class' column is missing in the
  'daa_results_filtered_sub_df' data frame. ",
  "Please use the 'pathway_annotation' function to annotate the 'pathway_daa' results."
  )
   }
  order <- order(
  daa_results_filtered_sub_df$pathway_class,
  daa_results_filtered_sub_df$p_adjust
    )
      },
      {
        order <- order
      }
    )

daa_results_filtered_sub_df <- daa_results_filtered_sub_df[order,]
error_bar_pivot_longer_tibble_summarised_ordered <- data.frame(name = NULL,
        group = NULL,
        mean = NULL,
        sd = NULL
      )
  for (i in daa_results_filtered_sub_df$feature) {
  error_bar_pivot_longer_tibble_summarised_ordered <-
  rbind(
  error_bar_pivot_longer_tibble_summarised_ordered,
  error_bar_pivot_longer_tibble_summarised
  [error_bar_pivot_longer_tibble_summarised$name ==
                                              i,]
        )
    }
    if (ko_to_kegg == FALSE){
      error_bar_pivot_longer_tibble_summarised_ordered[, x_lab] <-
        rep(daa_results_filtered_sub_df[, x_lab], each = length(levels(
          factor(error_bar_pivot_longer_tibble_summarised_ordered$group)
        )))
    }

    error_bar_pivot_longer_tibble_summarised_ordered$name <-
  factor(error_bar_pivot_longer_tibble_summarised_ordered$description,
         levels = unique(rev(daa_results_filtered_sub_df$description)))
```

```
    #daa_results_filtered_sub_df$feature <- factor(daa_results_filtered_sub_df$feature,
    #levels = unique(rev(daa_results_filtered_sub_df$feature)))
ordem <- unique(rev(daa_results_filtered_sub_df$description))
ordem

error_bar_pivot_longer_tibble_summarised_ordered <-
  error_bar_pivot_longer_tibble_summarised_ordered[!duplicated
  (error_bar_pivot_longer_tibble_summarised_ordered[c(1,2)]),]

#Exporting
export(error_bar_pivot_longer_tibble_summarised_ordered,
       "metab_predic_bar_errorbar.xlsx")

bar_errorbar <- ggplot2::ggplot(error_bar_pivot_longer_tibble_summarised_ordered,
                                #nolint: object_usage_linter.
             ggplot2::aes(mean, name, fill = group)) + # nolint
        #ggplot2::geom_errorbar(
        #ggplot2::aes(xmax = mean + sd, xmin = 0),
        #position = ggplot2::position_dodge2(width = 0.8, reverse = TRUE),
        #width = 0.8,
        #size = 0.5,
        #color = "black"
      #) +
  ggplot2::geom_bar(stat = "identity",
  position = ggplot2::position_dodge2(width = 0.8, reverse = TRUE), width = 0.8) +
  GGally::geom_stripped_cols(width = 10) +
  ggplot2::scale_fill_manual(values = colors) +
  ggplot2::scale_color_manual(values = colors) +
  ggprism::theme_prism() +
  ggplot2::scale_x_continuous(expand = c(0, 0),
                 guide = "prism_offset_minor",) +
  ggplot2::scale_y_discrete
  (labels = rev(error_bar_pivot_longer_tibble_summarised_ordered[, x_lab])) +
  ggplot2::labs(x = "Relative Abundance", y = NULL) +
  ggplot2::theme(
  axis.ticks.y = ggplot2::element_blank(),
  axis.line.y = ggplot2::element_blank(),
  axis.line.x = ggplot2::element_line(size = 0.5),
  axis.ticks.x = ggplot2::element_line(size = 0.5),
  panel.grid.major.y = ggplot2::element_blank(),
  panel.grid.major.x = ggplot2::element_blank(),
  axis.text = ggplot2::element_text(size = 5, color = "black"), # nolint
  axis.text.x = ggplot2::element_text(margin = ggplot2::margin(r = 0)), # nolint
  axis.text.y = ggplot2::element_text(
          size = 5,
          color = "black",
          margin = ggplot2::margin(b = 6, l = 6)
        ),
        axis.title.x =  ggplot2::element_text(
          size = 5,
          color = "black",
          hjust = 0.5
        ),
```

```
        legend.position = "right",
        legend.key.size = ggplot2::unit(0.1, "cm"),
        legend.direction = "vertical",
        legend.justification = "left",
        legend.text = ggplot2::element_text(size = 5, face = "bold"),
        legend.box.just = "right",
        plot.margin = ggplot2::margin(0, 0.5, 0.5, 0, unit = "cm")
      ) + ggplot2::coord_cartesian(clip = "off")

bar_errorbar

daa_results_filtered_sub_df <-
      cbind(
        daa_results_filtered_sub_df,
        negative_log10_p = -log10(daa_results_filtered_sub_df$p_adjust),
        group_nonsense = "nonsense",
        log_2_fold_change = NA
      )

daa_results_filtered_sub_df$comparation <- paste(sep=" - ",
          daa_results_filtered_sub_df$group1,daa_results_filtered_sub_df$group2)

# Iteration
iterar <- c("M-CoV", "NO-CoV", "S-CoV", "NC")

for (i in daa_results_filtered_sub_df$description){

  for(versus in iterar) {
  valVersus <-
  error_bar_pivot_longer_tibble_summarised_ordered[
    error_bar_pivot_longer_tibble_summarised_ordered$name %in% i &
    error_bar_pivot_longer_tibble_summarised_ordered$group==versus,]$mean

  iterar2 <-
  daa_results_filtered_sub_df[daa_results_filtered_sub_df$description==i &
                              daa_results_filtered_sub_df$group2==versus &
                              daa_results_filtered_sub_df$group1 %in% iterar,]$group1
  for(j in iterar2) {
  valData <-
  error_bar_pivot_longer_tibble_summarised_ordered[
    error_bar_pivot_longer_tibble_summarised_ordered$name %in% i &
    error_bar_pivot_longer_tibble_summarised_ordered$group==j,]$mean

  if(!is.na(valData) && !is.na(valVersus)) {
  daa_results_filtered_sub_df[daa_results_filtered_sub_df$description==i &
                              daa_results_filtered_sub_df$group2==versus &
  daa_results_filtered_sub_df$group1==j,]$log_2_fold_change <- log2(valData/valVersus)
        }
      }
      }

}
```

```r
#End

    daa_results_filtered_sub_df$description <-
    factor(daa_results_filtered_sub_df$description,levels =
             unique(rev(daa_results_filtered_sub_df$description)))

    #Mutating for plotting
    daa_results_filtered_sub_df <- daa_results_filtered_sub_df %>%
  mutate(log_2_fold_change = ifelse(comparation == "NC - S-CoV" | comparation ==
                                     "NC - M-CoV",
                                    -1 * log_2_fold_change,
                                    log_2_fold_change))

    daa_results_filtered_sub_df <- daa_results_filtered_sub_df %>%
  mutate(comparation = case_when(
    comparation == "NC - S-CoV" ~ "S-CoV - NC",
    comparation == "NC - M-CoV" ~ "M-CoV - NC",
    TRUE ~ comparation
  ))

    #Exporting
    export(daa_results_filtered_sub_df, "daa_pathways.xlsx")

    # Define the custom order of levels for the "comparation" column
custom_order <- c("M-CoV - NC", "S-CoV - NC", "NO-CoV - M-CoV", "S-CoV - NO-CoV",
                  "S-CoV - M-CoV")

# Update the "comparation" column to use the custom order
daa_results_filtered_sub_df$comparation <-
  factor(daa_results_filtered_sub_df$comparation, levels = custom_order)

# Plotting code with updated order
p_values_bar <- daa_results_filtered_sub_df %>%
  ggplot2::ggplot(ggplot2::aes(factor(description,
                      level = sort(unique(daa_results_filtered_sub_df$description))),
                      log_2_fold_change, fill = log_2_fold_change > 0)) +
  ggplot2::geom_bar(stat = "identity",
                    position = ggplot2::position_dodge(width = 0.8),
                    width = 0.8) +
  ggplot2::labs(y = "log2 fold change", x = NULL) +
  GGally::geom_stripped_cols() +
  ggplot2::scale_fill_manual(
    values = c("TRUE" = "blue", "FALSE" = "red"),
    breaks = c(TRUE, FALSE),
    labels = c("Positive", "Negative")
  ) +
  ggplot2::scale_color_manual(values = "#87ceeb") +
  ggplot2::scale_x_discrete(labels =
  rev(error_bar_pivot_longer_tibble_summarised_ordered[, x_lab])) +
  ggplot2::geom_hline(ggplot2::aes(yintercept = 0),
                     linetype = 'dashed',
                     color = 'black') +
  ggprism::theme_prism() +
```

```
  ggplot2::scale_y_continuous(expand = c(-2, 0),
                              guide = "prism_offset_minor") +
  ylim(-4, 4) +
  ggplot2::theme(
    axis.ticks.y = ggplot2::element_blank(),
    axis.line.y = ggplot2::element_blank(),
    axis.line.x = ggplot2::element_line(size = 0.5),
    axis.ticks.x = ggplot2::element_line(size = 0.5),
    panel.grid.major.y = ggplot2::element_blank(),
    panel.grid.major.x = ggplot2::element_blank(),
    axis.text = ggplot2::element_text(size = 5, color = "black"),
    axis.text.x = ggplot2::element_text(
      size = 5,
      color = "black",
      margin = ggplot2::margin(b = 6)
    ),
    axis.title.x =  ggplot2::element_text(
      size = 5,
      color = "black",
      hjust = 0.5
    ),
    strip.text = ggplot2::element_text(size = 8),
    legend.position = "right"
  ) +
  ggplot2::coord_flip() +
  ggplot2::facet_grid(. ~ comparation, scales =
                        "free_x", space = "free_x", switch = "y")


    p_values_bar


# Create labels for 'A' and 'B'
label_A <- letters[1]
label_B <- letters[2]

# Combine the two plots with the specified layout
combination_bar_plot <- (
  (bar_errorbar + labs(tag = label_A)) /
  (p_values_bar + labs(tag = label_B))
) +
plot_layout(nrow = 2)

ggsave("Fig_Metabolic_prediction_FULL.pdf", plot = combination_bar_plot,
       width = 180, height = 225,
       units = "mm", dpi = 600)
```

A total of 146 metabolic pathways were differentially abundant. With this number, it not possible to generate a good plot for visualization, so we filtered the data and the plot is presented in the main article. All the calculated data is presented in Supplementary Data 11-12.

```
# Metabolic prediction - MetaCyc Pathways - Filtered for proper plotting
#This chunk filter the TOP metabolic pathways by their relative abundance
#for proper plotting.
```

```r
library(phyloseq)
library(readr)
library(ggpicrust2)
library(tibble)
library(tidyverse)
library(ggprism)
library(patchwork)
library(dplyr)
abundance_file <-
  "/home/metagenomica/LABRESIS/OtavioLovison/Paper/PICRUSt2/picrust2_out_pipeline/
 pathways_out/path_abun_unstrat.tsv"
metadata <- read_delim(
    "/home/metagenomica/LABRESIS/OtavioLovison/Paper/PICRUSt2/sample_metadata.txt",
    delim = "\t",
    escape_double = FALSE,
    trim_ws = TRUE
)

metacyc_abundance <-
  read_delim(abundance_file, delim = "\t", col_names = TRUE, trim_ws = TRUE)

#DAA1
abundance <- metacyc_abundance %>% column_to_rownames("pathway")
group <- "COVID19"
reference <- "Group 1"
formula <- "~Age + Sex + batch + Group_group_nonsense_"
p.adjust <- "BH"

if (!tibble::is_tibble(metadata)) {
        message("Converting metadata to tibble...")
        metadata <- tibble::as_tibble(metadata)
    }
    sample_names <- colnames(abundance)
    message("Sample names extracted.")
    message("Identifying matching columns in metadata...")
    matches <- base::lapply(metadata, function(x) {
        intersect(sample_names, x)
    })
    matching_columns <- names(metadata)[sapply(matches, function(x) {
        length(x) == length(sample_names)
    })][1]
    if (!is.null(matching_columns)) {
        message(paste("Matching columns identified:", matching_columns,
            ". This is important for ensuring data consistency."))
    }

    switch(is.null(select), `TRUE` = {
        message("Using all columns in abundance.")
    }, `FALSE` = {
        message("Filtering the abundance and metadata...")
        abundance <- abundance[, colnames(abundance) %in% select]
        metadata <- metadata[as.matrix(metadata[, matching_columns]) %in%
            select, ]
```

```
})
sample_names <- colnames(abundance)
message("Converting abundance to a matrix...")
abundance_mat <- as.matrix(abundance)
message("Reordering metadata...")
metadata_order <- match(sample_names, as.matrix(metadata[,
    matching_columns]))
metadata <- metadata[metadata_order, ]
message("Converting metadata to a matrix and data frame...")
metadata_mat <- as.matrix(metadata)
metadata_df <- as.data.frame(metadata)
message("Extracting group information...")
Group <- factor(metadata_mat[, group])
Level <- levels(Group)
length_Level <- length(Level)

    LinDA_metadata_df <- metadata_df
    LinDA_colnames <- colnames(LinDA_metadata_df)
    LinDA_colnames[LinDA_colnames == group] <- "Group_group_nonsense_"
    colnames(LinDA_metadata_df) <- LinDA_colnames
    rownames(LinDA_metadata_df) <- LinDA_metadata_df[, matching_columns]
    LinDA_metadata_df <- dplyr::select(LinDA_metadata_df, -matching_columns)
    LinDA_metadata_df$Group_group_nonsense_ <-
      factor(LinDA_metadata_df$Group_group_nonsense_)
    if (length_Level != 2) {
        if (is.null(reference)) {
            stop("Error: A reference group is required when using LinDA or
                 limma voom for comparisons among more than two groups.
                 Please specify a reference group.")
        }
        LinDA_metadata_df$Group_group_nonsense_ <-
          stats::relevel(LinDA_metadata_df$Group_group_nonsense_,
            ref = reference)
    }
    message("Performing LinDA analysis...")
    LinDA_results <- MicrobiomeStat::linda(abundance, LinDA_metadata_df,
        formula = formula, alpha = 0.05)$output
    message("Processing LinDA results...")
    length(LinDA_results)
    if (length_Level != 2) {
        for (i in 1:length(LinDA_results)) {
            LinDA_results[[i]] <- cbind(feature = rownames(LinDA_results[[i]]),
              method = "LinDA", group1 = substr(names(LinDA_results)[i],
                22, stop = nchar(names(LinDA_results)[i])),
              group2 = reference, p_values = LinDA_results[[i]]$pvalue)
        }
    } else {
        for (i in 1:length(LinDA_results)) {
            LinDA_results[[i]] <- cbind(feature = rownames(LinDA_results[[i]]),
              method = "LinDA", group1 = Level[1], group2 = Level[2],
              p_values = LinDA_results[[i]]$pvalue)
        }
    }
```

```
        message("LinDA analysis is complete.")
        LinDA_results <- LinDA_results[7:length(LinDA_results)]
        p_values_matrix <- as.matrix(do.call(rbind, LinDA_results))
        p_values_df <- as.data.frame(p_values_matrix)

        valid_p_adjust <- c("BH", "holm", "bonferroni", "hochberg",
        "fdr", "none")
    if (!p.adjust %in% valid_p_adjust) {
        stop(paste("Invalid p.adjust method. Please choose from:",
            paste(valid_p_adjust, collapse = ", ")))
    }
    if (!exists("p_values_df") || nrow(p_values_df) == 0) {
    stop("Notice: There are no statistical significances detected. This is not an error,
      but it might indicate that your data does not contain any values passing the set
      significance threshold (p<=0.05).
      You may refer to the tutorial's FAQ for further help and suggestions.")
    }
    switch(p.adjust, BH = {
        adjusted_p_values <- p.adjust(p_values_df$p_values, method = "BH")
    }, holm = {
        adjusted_p_values <- p.adjust(p_values_df$p_values, method = "holm")
    }, bonferroni = {
        adjusted_p_values <- p.adjust(p_values_df$p_values, method = "bonferroni")
    }, hochberg = {
        adjusted_p_values <- p.adjust(p_values_df$p_values, method = "hochberg")
    }, fdr = {
        adjusted_p_values <- p.adjust(p_values_df$p_values, method = "fdr")
    }, none = {
        adjusted_p_values <- p.adjust(p_values_df$p_values, method = "none")
    })
    daa_results_df1 <- cbind(p_values_df, adj_method = p.adjust,
        p_adjust = adjusted_p_values)

#################################################################################
#DAA2
abundance <- metacyc_abundance %>% column_to_rownames("pathway")
group <- "COVID19"
reference <- "Group 2"
formula <- "~Age + Sex + batch + Group_group_nonsense_"
p.adjust <- "BH"

if (!tibble::is_tibble(metadata)) {
        message("Converting metadata to tibble...")
        metadata <- tibble::as_tibble(metadata)
    }
    sample_names <- colnames(abundance)
    message("Sample names extracted.")
    message("Identifying matching columns in metadata...")
    matches <- base::lapply(metadata, function(x) {
        intersect(sample_names, x)
    })
    matching_columns <- names(metadata)[sapply(matches, function(x) {
        length(x) == length(sample_names)
```

```r
    })][1]
    if (!is.null(matching_columns)) {
        message(paste("Matching columns identified:", matching_columns,
            ". This is important for ensuring data consistency."))
    }

    switch(is.null(select), `TRUE` = {
        message("Using all columns in abundance.")
    }, `FALSE` = {
        message("Filtering the abundance and metadata...")
        abundance <- abundance[, colnames(abundance) %in% select]
        metadata <- metadata[as.matrix(metadata[, matching_columns]) %in%
            select, ]
    })
    sample_names <- colnames(abundance)
    message("Converting abundance to a matrix...")
    abundance_mat <- as.matrix(abundance)
    message("Reordering metadata...")
    metadata_order <- match(sample_names, as.matrix(metadata[,
        matching_columns]))
    metadata <- metadata[metadata_order, ]
    message("Converting metadata to a matrix and data frame...")
    metadata_mat <- as.matrix(metadata)
    metadata_df <- as.data.frame(metadata)
    message("Extracting group information...")
    Group <- factor(metadata_mat[, group])
    Level <- levels(Group)
    length_Level <- length(Level)

        LinDA_metadata_df <- metadata_df
        LinDA_colnames <- colnames(LinDA_metadata_df)
        LinDA_colnames[LinDA_colnames == group] <- "Group_group_nonsense_"
        colnames(LinDA_metadata_df) <- LinDA_colnames
        rownames(LinDA_metadata_df) <- LinDA_metadata_df[, matching_columns]
        LinDA_metadata_df <- dplyr::select(LinDA_metadata_df,
            -matching_columns)
        LinDA_metadata_df$Group_group_nonsense_ <- factor(LinDA_metadata_df$
                                                          Group_group_nonsense_)
        if (length_Level != 2) {
            if (is.null(reference)) {
                stop("Error: A reference group is required when using LinDA or
                     limma voom for comparisons among more than two groups.
                     Please specify a reference group.")
            }
            LinDA_metadata_df$Group_group_nonsense_ <-
              stats::relevel(LinDA_metadata_df$Group_group_nonsense_,
                ref = reference)
        }
        message("Performing LinDA analysis...")
        LinDA_results <- MicrobiomeStat::linda(abundance, LinDA_metadata_df,
            formula = formula, alpha = 0.05)$output
        message("Processing LinDA results...")
        length(LinDA_results)
```

```r
        if (length_Level != 2) {
            for (i in 1:length(LinDA_results)) {
                LinDA_results[[i]] <- cbind(feature = rownames(LinDA_results[[i]]),
                  method = "LinDA", group1 = substr(names(LinDA_results)[i],
                    22, stop = nchar(names(LinDA_results)[i])),
                  group2 = reference, p_values = LinDA_results[[i]]$pvalue)
            }
        } else {
            for (i in 1:length(LinDA_results)) {
                LinDA_results[[i]] <- cbind(feature = rownames(LinDA_results[[i]]),
                  method = "LinDA", group1 = Level[1], group2 = Level[2],
                  p_values = LinDA_results[[i]]$pvalue)
            }
        }
        message("LinDA analysis is complete.")
        LinDA_results <- LinDA_results[7:length(LinDA_results)]
        p_values_matrix <- as.matrix(do.call(rbind, LinDA_results))
        p_values_df <- as.data.frame(p_values_matrix)

        valid_p_adjust <- c("BH", "holm", "bonferroni", "hochberg",
        "fdr", "none")
    if (!p.adjust %in% valid_p_adjust) {
        stop(paste("Invalid p.adjust method. Please choose from:",
            paste(valid_p_adjust, collapse = ", ")))
    }
    if (!exists("p_values_df") || nrow(p_values_df) == 0) {
    stop("Notice: There are no statistical significances detected.
      This is not an error, but it might indicate that your data does not contain
      any values passing the set significance threshold (p<=0.05).
      You may refer to the tutorial's FAQ for further help and suggestions.")
    }
    switch(p.adjust, BH = {
        adjusted_p_values <- p.adjust(p_values_df$p_values, method = "BH")
    }, holm = {
        adjusted_p_values <- p.adjust(p_values_df$p_values, method = "holm")
    }, bonferroni = {
        adjusted_p_values <- p.adjust(p_values_df$p_values, method = "bonferroni")
    }, hochberg = {
        adjusted_p_values <- p.adjust(p_values_df$p_values, method = "hochberg")
    }, fdr = {
        adjusted_p_values <- p.adjust(p_values_df$p_values, method = "fdr")
    }, none = {
        adjusted_p_values <- p.adjust(p_values_df$p_values, method = "none")
    })
    daa_results_df2 <- cbind(p_values_df, adj_method = p.adjust,
        p_adjust = adjusted_p_values)

################################################################################
#DAA3
abundance <- metacyc_abundance %>% column_to_rownames("pathway")
group <- "COVID19"
reference <- "Group 3"
formula <- "~Age + Sex + batch + Group_group_nonsense_"
```

```
p.adjust <- "BH"

if (!tibble::is_tibble(metadata)) {
        message("Converting metadata to tibble...")
        metadata <- tibble::as_tibble(metadata)
    }
    sample_names <- colnames(abundance)
    message("Sample names extracted.")
    message("Identifying matching columns in metadata...")
    matches <- base::lapply(metadata, function(x) {
        intersect(sample_names, x)
    })
    matching_columns <- names(metadata)[sapply(matches, function(x) {
        length(x) == length(sample_names)
    })][1]
    if (!is.null(matching_columns)) {
        message(paste("Matching columns identified:", matching_columns,
            ". This is important for ensuring data consistency."))
    }

    switch(is.null(select), `TRUE` = {
        message("Using all columns in abundance.")
    }, `FALSE` = {
        message("Filtering the abundance and metadata...")
        abundance <- abundance[, colnames(abundance) %in% select]
        metadata <- metadata[as.matrix(metadata[, matching_columns]) %in%
            select, ]
    })
    sample_names <- colnames(abundance)
    message("Converting abundance to a matrix...")
    abundance_mat <- as.matrix(abundance)
    message("Reordering metadata...")
    metadata_order <- match(sample_names, as.matrix(metadata[,
        matching_columns]))
    metadata <- metadata[metadata_order, ]
    message("Converting metadata to a matrix and data frame...")
    metadata_mat <- as.matrix(metadata)
    metadata_df <- as.data.frame(metadata)
    message("Extracting group information...")
    Group <- factor(metadata_mat[, group])
    Level <- levels(Group)
    length_Level <- length(Level)

        LinDA_metadata_df <- metadata_df
        LinDA_colnames <- colnames(LinDA_metadata_df)
        LinDA_colnames[LinDA_colnames == group] <- "Group_group_nonsense_"
        colnames(LinDA_metadata_df) <- LinDA_colnames
        rownames(LinDA_metadata_df) <- LinDA_metadata_df[, matching_columns]
        LinDA_metadata_df <- dplyr::select(LinDA_metadata_df,
            -matching_columns)
        LinDA_metadata_df$Group_group_nonsense_ <- factor(LinDA_metadata_df$
                                                          Group_group_nonsense_)
        if (length_Level != 2) {
```

```
        if (is.null(reference)) {
            stop("Error: A reference group is required when using LinDA or
                 limma voom for comparisons among more than two groups.
                 Please specify a reference group.")
        }
        LinDA_metadata_df$Group_group_nonsense_ <-
          stats::relevel(LinDA_metadata_df$Group_group_nonsense_,
            ref = reference)
    }
    message("Performing LinDA analysis...")
    LinDA_results <- MicrobiomeStat::linda(abundance, LinDA_metadata_df,
        formula = formula, alpha = 0.05)$output
    message("Processing LinDA results...")
    length(LinDA_results)
    if (length_Level != 2) {
        for (i in 1:length(LinDA_results)) {
            LinDA_results[[i]] <- cbind(feature = rownames(LinDA_results[[i]]),
              method = "LinDA", group1 = substr(names(LinDA_results)[i],
                22, stop = nchar(names(LinDA_results)[i])),
              group2 = reference, p_values = LinDA_results[[i]]$pvalue)
        }
    } else {
        for (i in 1:length(LinDA_results)) {
            LinDA_results[[i]] <- cbind(feature = rownames(LinDA_results[[i]]),
              method = "LinDA", group1 = Level[1], group2 = Level[2],
              p_values = LinDA_results[[i]]$pvalue)
        }
    }
    message("LinDA analysis is complete.")
    LinDA_results <- LinDA_results[7:length(LinDA_results)]
    p_values_matrix <- as.matrix(do.call(rbind, LinDA_results))
    p_values_df <- as.data.frame(p_values_matrix)

    valid_p_adjust <- c("BH", "holm", "bonferroni", "hochberg",
    "fdr", "none")
if (!p.adjust %in% valid_p_adjust) {
    stop(paste("Invalid p.adjust method. Please choose from:",
        paste(valid_p_adjust, collapse = ", ")))
}
if (!exists("p_values_df") || nrow(p_values_df) == 0) {
stop("Notice: There are no statistical significances detected. This is not an error,
but it might indicate that your data does not contain any values passing the set
significance threshold (p<=0.05).
You may refer to the tutorial's FAQ for further help and suggestions.")
}
switch(p.adjust, BH = {
    adjusted_p_values <- p.adjust(p_values_df$p_values, method = "BH")
}, holm = {
    adjusted_p_values <- p.adjust(p_values_df$p_values, method = "holm")
}, bonferroni = {
    adjusted_p_values <- p.adjust(p_values_df$p_values, method = "bonferroni")
}, hochberg = {
    adjusted_p_values <- p.adjust(p_values_df$p_values, method = "hochberg")
```

```
    }, fdr = {
        adjusted_p_values <- p.adjust(p_values_df$p_values, method = "fdr")
    }, none = {
        adjusted_p_values <- p.adjust(p_values_df$p_values, method = "none")
    })
    daa_results_df3 <- cbind(p_values_df, adj_method = p.adjust,
        p_adjust = adjusted_p_values)

##################################################################################

# Annotation
metacyc_daa_annotated_results_df1 <- pathway_annotation(pathway = "MetaCyc",
                                daa_results_df = daa_results_df1, ko_to_kegg = FALSE)
metacyc_daa_annotated_results_df2 <- pathway_annotation(pathway = "MetaCyc",
                                daa_results_df = daa_results_df2, ko_to_kegg = FALSE)
metacyc_daa_annotated_results_df3 <- pathway_annotation(pathway = "MetaCyc",
                                daa_results_df = daa_results_df3, ko_to_kegg = FALSE)

#Filtering duplicates
filtered_df2 <- metacyc_daa_annotated_results_df2 %>%
  filter(group1 != "Group 1")

filtered_df3 <- metacyc_daa_annotated_results_df3 %>%
  filter(group1 != "Group 1" & group1 != "Group 2")

#Concatenating
metacyc_daa_all_results_df <-
  rbind(metacyc_daa_annotated_results_df1, filtered_df2, filtered_df3)

#Adjusting variable names for plotting
metacyc_daa_all_results_df$group1[metacyc_daa_all_results_df$group1 == "Group 2"] <-
  "NO-CoV"
metacyc_daa_all_results_df$group1[metacyc_daa_all_results_df$group1 == "Group 3"] <-
  "S-CoV"
metacyc_daa_all_results_df$group1[metacyc_daa_all_results_df$group1 == "Group 4"] <-
  "NC"
metacyc_daa_all_results_df$group2[metacyc_daa_all_results_df$group2 == "Group 1"] <-
  "M-CoV"
metacyc_daa_all_results_df$group2[metacyc_daa_all_results_df$group2 == "Group 2"] <-
  "NO-CoV"
metacyc_daa_all_results_df$group2[metacyc_daa_all_results_df$group2 == "Group 3"] <-
  "S-CoV"
metadata$COVID19[metadata$COVID19 == "Group 1"] <- "M-CoV"
metadata$COVID19[metadata$COVID19 == "Group 2"] <- "NO-CoV"
metadata$COVID19[metadata$COVID19 == "Group 3"] <- "S-CoV"
metadata$COVID19[metadata$COVID19 == "Group 4"] <- "NC"

# Making the plot
abundance = metacyc_abundance %>% column_to_rownames("pathway")
daa_results_df = metacyc_daa_all_results_df
Group = metadata$COVID19
p_values_threshold = 0.05
order = "group"
```

```r
select = NULL
ko_to_kegg = FALSE
p_value_bar = TRUE
colors = c("M-CoV"="#68228B","NO-CoV"="#7FFF00","S-CoV"="#00bfff","NC"="#FF521C")
x_lab = "description"

# Identifying missing pathways
missing_pathways <- daa_results_df[is.na(daa_results_df$pathway_name), "feature"]

# Extracting colnames
column_names <- colnames(daa_results_df)

# Excluding missing annotations
daa_results_df <- daa_results_df[!is.na(daa_results_df[,x_lab]),]

#Assigning abundance matrix
errorbar_abundance_mat <- as.matrix(abundance)

#Filtering statistically significant results
daa_results_filtered_df <-
      daa_results_df[daa_results_df$p_adjust < p_values_threshold,]

if (!is.null(select)) {
  daa_results_filtered_sub_df <-
        daa_results_filtered_df[daa_results_filtered_df$feature %in% select, ]
    } else {
      daa_results_filtered_sub_df <- daa_results_filtered_df
    }

    if (nrow(daa_results_filtered_sub_df) > 30) {
      message(
        paste0(
    "The number of features with statistical significance exceeds 30,
    leading to suboptimal visualization. ",
    "Please use 'select' to reduce the number of features.\n",
    "Currently, you have these features: ",
    paste(paste0('"', daa_results_filtered_sub_df$feature, '"'), collapse = ", "), ".\n",
    "You can find the statistically significant features with the following command:\n",
    "daa_results_df %>% filter(p_adjust < 0.05) %>% select(c(\"feature\",\"p_adjust\"))"
        )
      )
      # stop()
    }

    if (nrow(daa_results_filtered_sub_df) == 0){
    stop(
    "Visualization with 'pathway_errorbar' cannot be performed because there
    are no features with statistical significance. ",
    "For possible solutions, please check the FAQ section of the tutorial."
      )
    }

# Convert to relative abundance
```

```r
relative_abundance_mat <- apply(t(errorbar_abundance_mat), 1, function(x)
  x / sum(x))

# Subset to only include the features present in daa_results_filtered_sub_df$feature
sub_relative_abundance_mat <-
  relative_abundance_mat[rownames(relative_abundance_mat) %in%
                                                   daa_results_filtered_sub_df$feature,]

# Create a matrix for the error bars
error_bar_matrix <- cbind(
      sample = colnames(sub_relative_abundance_mat),
      group = Group,
      t(sub_relative_abundance_mat)
    )

error_bar_df <- as.data.frame(error_bar_matrix)
error_bar_df$group <- factor(Group, levels = levels(as.factor(Group)))
error_bar_pivot_longer_df <- tidyr::pivot_longer(error_bar_df,-c(sample, group))
error_bar_pivot_longer_tibble <-
  mutate(error_bar_pivot_longer_df, group = as.factor(group))
error_bar_pivot_longer_tibble$sample <- factor(error_bar_pivot_longer_tibble$sample)
error_bar_pivot_longer_tibble$name <- factor(error_bar_pivot_longer_tibble$name)
error_bar_pivot_longer_tibble$value <- as.numeric(error_bar_pivot_longer_tibble$value)

error_bar_pivot_longer_tibble_summarised <-
  error_bar_pivot_longer_tibble %>% group_by(name, group) %>%
  summarise(mean = mean(value), sd = stats::sd(value))

  switch(
  order,
  "p_values" = {
  #order <- order(daa_results_filtered_sub_df$p_adjust)
  order <- order(daa_results_filtered_sub_df$feature)
  },
  "name" = {
  order <- order(daa_results_filtered_sub_df$feature)
  },
  "group" = {
  daa_results_filtered_sub_df$pro <- 1
  for (i in levels(error_bar_pivot_longer_tibble_summarised$name)) {
  error_bar_pivot_longer_tibble_summarised_sub <-
  error_bar_pivot_longer_tibble_summarised
  [error_bar_pivot_longer_tibble_summarised$name ==
                                                    i,]
  pro_group <-
  error_bar_pivot_longer_tibble_summarised_sub
  [error_bar_pivot_longer_tibble_summarised_sub$mean ==
                         max(error_bar_pivot_longer_tibble_summarised_sub$mean),]$group
          pro_group <- as.vector(pro_group)
          daa_results_filtered_sub_df[daa_results_filtered_sub_df$feature ==
                                      i,]$pro <- pro_group
      }
  order <-
```

```r
  order(daa_results_filtered_sub_df$pro,
  daa_results_filtered_sub_df$p_adjust)
      },
    "pathway_class" = {
    if (!"pathway_class" %in% colnames(daa_results_filtered_sub_df)) {
    stop(
    "The 'pathway_class' column is missing in the
    'daa_results_filtered_sub_df' data frame. ",
    "Please use the 'pathway_annotation' function to annotate the 'pathway_daa'
    results."
          )
        }
        order <- order(
          daa_results_filtered_sub_df$pathway_class,
          daa_results_filtered_sub_df$p_adjust
        )
      },
      {
        order <- order
      }
    )

daa_results_filtered_sub_df <- daa_results_filtered_sub_df[order,]
error_bar_pivot_longer_tibble_summarised_ordered <- data.frame(name = NULL,
        group = NULL,
        mean = NULL,
        sd = NULL
      )
 for (i in daa_results_filtered_sub_df$feature) {
 error_bar_pivot_longer_tibble_summarised_ordered <-
 rbind(
 error_bar_pivot_longer_tibble_summarised_ordered,
 error_bar_pivot_longer_tibble_summarised[error_bar_pivot_longer_tibble_summarised$name ==
                                                  i,]
        )
  }
 if (ko_to_kegg == FALSE){
 error_bar_pivot_longer_tibble_summarised_ordered[, x_lab] <-
 rep(daa_results_filtered_sub_df[, x_lab], each = length(levels(
 factor(error_bar_pivot_longer_tibble_summarised_ordered$group)
        )))
    }

    error_bar_pivot_longer_tibble_summarised_ordered$name <-
      factor(error_bar_pivot_longer_tibble_summarised_ordered$description,
             levels = unique(rev(daa_results_filtered_sub_df$description)))

ordem <- unique(rev(daa_results_filtered_sub_df$description))
ordem

error_bar_pivot_longer_tibble_summarised_ordered <-
  error_bar_pivot_longer_tibble_summarised_ordered[
  !duplicated(error_bar_pivot_longer_tibble_summarised_ordered[c(1,2)]),]
```

```r
#Filtering for plotting
# Group by "name" and calculate the sum of "mean" values
error_bar_pivot_longer_tibble_summarised_ordered_filtered <-
  error_bar_pivot_longer_tibble_summarised_ordered %>%
  group_by(name) %>%
  summarize(total_mean = sum(mean)) %>%
  ungroup() %>%
  arrange(desc(total_mean)) %>%
  head(30)

# Filter the original dataframe to keep only the top 30 "names"
top_30_names <- error_bar_pivot_longer_tibble_summarised_ordered %>%
  filter(name %in% error_bar_pivot_longer_tibble_summarised_ordered_filtered$name)

bar_errorbar <- ggplot2::ggplot(top_30_names, # nolint: object_usage_linter.
             ggplot2::aes(mean, name, fill = group)) + # nolint
        ggplot2::geom_errorbar(
        ggplot2::aes(xmax = mean + sd, xmin = 0),
        position = ggplot2::position_dodge2(width = 0.8, reverse = TRUE),
        width = 0.8,
        size = 0.5,
        color = "black"
      ) +
      ggplot2::geom_bar(stat = "identity", position =
                          ggplot2::position_dodge2(width = 0.8, reverse = TRUE),
               width = 0.8) +
      GGally::geom_stripped_cols(width = 10) +
      ggplot2::scale_fill_manual(values = colors) +
      ggplot2::scale_color_manual(values = colors) +
      ggprism::theme_prism() +
      ggplot2::scale_x_continuous(expand = c(0, 0),
                         guide = "prism_offset_minor",) +
      ggplot2::scale_y_discrete(labels = rev(top_30_names[, x_lab])) +
      ggplot2::labs(x = "Relative Abundance", y = NULL) +
      ggplot2::theme(
        axis.ticks.y = ggplot2::element_blank(),
        axis.line.y = ggplot2::element_blank(),
        axis.line.x = ggplot2::element_line(size = 0.5),
        axis.ticks.x = ggplot2::element_line(size = 0.5),
        panel.grid.major.y = ggplot2::element_blank(),
        panel.grid.major.x = ggplot2::element_blank(),
        axis.text = ggplot2::element_text(size = 5, color = "black"), # nolint
        axis.text.x = ggplot2::element_text(margin = ggplot2::margin(r = 0)), # nolint
        axis.text.y = ggplot2::element_text(
          size = 5,
          color = "black",
          margin = ggplot2::margin(b = 6, l = 6)
        ),
        axis.title.x =  ggplot2::element_text(
          size = 5,
          color = "black",
          hjust = 0.5
        ),
```

```
        legend.position = "right",
        legend.key.size = ggplot2::unit(0.1, "cm"),
        legend.direction = "vertical",
        legend.justification = "left",
        legend.text = ggplot2::element_text(size = 5, face = "bold"),
        legend.box.just = "right",
        plot.margin = ggplot2::margin(0, 0.5, 0.5, 0, unit = "cm")
      ) + ggplot2::coord_cartesian(clip = "off")

ggsave("bar_errorbar.pdf", plot = bar_errorbar, width = 180, height = 225,
       units = "mm", dpi = 600)
ggsave("bar_errorbar.svg", plot = bar_errorbar, width = 180, height = 185,
       units = "mm", dpi = 600)

daa_results_filtered_sub_df <-
      cbind(
        daa_results_filtered_sub_df,
        negative_log10_p = -log10(daa_results_filtered_sub_df$p_adjust),
        group_nonsense = "nonsense",
        log_2_fold_change = NA
      )

daa_results_filtered_sub_df$comparation <- paste(sep=" - ",
             daa_results_filtered_sub_df$group1,daa_results_filtered_sub_df$group2)

# Extract the unique descriptions from the "top_30_names"
top_30_descriptions <- unique(top_30_names$description)

# Filter "daa_results_filtered_sub_df" to keep rows with descriptions in
# "top_30_descriptions"
filtered_daa_results_filtered_sub_df <- daa_results_filtered_sub_df %>%
  filter(description %in% top_30_descriptions)

# remendissimo
iterar <- c("M-CoV", "NO-CoV", "S-CoV", "NC")

for (i in filtered_daa_results_filtered_sub_df$description){
    #mean <- error_bar_pivot_longer_tibble_summarised_ordered
  #[error_bar_pivot_longer_tibble_summarised_ordered$name %in% i,]
  for(versus in iterar) {
  valVersus <- top_30_names[top_30_names$name %in% i &
                                  top_30_names$group==versus,]$mean

  iterar2 <- filtered_daa_results_filtered_sub_df[
  filtered_daa_results_filtered_sub_df$description==i &
  filtered_daa_results_filtered_sub_df$group2==versus &
  filtered_daa_results_filtered_sub_df$group1 %in% iterar,]$group1
    for(j in iterar2) {
  valData <- top_30_names[top_30_names$name %in% i & top_30_names$group==j,]$mean

  if(!is.na(valData) && !is.na(valVersus)) {
  filtered_daa_results_filtered_sub_df[
  filtered_daa_results_filtered_sub_df$description==i &
```

```
  filtered_daa_results_filtered_sub_df$group2==versus &
  filtered_daa_results_filtered_sub_df$group1==j,]$log_2_fold_change <-
    log2(valData/valVersus)
        }
      }
      }

}

#End

    filtered_daa_results_filtered_sub_df$description <-
      factor(filtered_daa_results_filtered_sub_df$description,levels =
               unique(rev(filtered_daa_results_filtered_sub_df$description)))

    #Mutating for plotting
    filtered_daa_results_filtered_sub_df <- filtered_daa_results_filtered_sub_df %>%
  mutate(log_2_fold_change = ifelse(comparation == "NC - S-CoV" |
                                     comparation == "NC - M-CoV",
                                    -1 * log_2_fold_change,
                                    log_2_fold_change))

    filtered_daa_results_filtered_sub_df <- filtered_daa_results_filtered_sub_df %>%
  mutate(comparation = case_when(
    comparation == "NC - S-CoV" ~ "S-CoV - NC",
    comparation == "NC - M-CoV" ~ "M-CoV - NC",
    TRUE ~ comparation
  ))

# Define the custom order of levels for the "comparation" column
custom_order <- c("M-CoV - NC", "S-CoV - NC", "NO-CoV - M-CoV",
                  "S-CoV - NO-CoV", "S-CoV - M-CoV")

# Update the "comparation" column to use the custom order
filtered_daa_results_filtered_sub_df$comparation <-
  factor(filtered_daa_results_filtered_sub_df$comparation, levels = custom_order)

# Plotting code with updated order
p_values_bar <- filtered_daa_results_filtered_sub_df %>%
  ggplot2::ggplot(ggplot2::aes(factor(description, level = sort(unique(description))),
                               log_2_fold_change, fill = log_2_fold_change > 0)) +
  ggplot2::geom_bar(stat = "identity",
           position = ggplot2::position_dodge(width = 0.8),
           width = 0.8) +
  ggplot2::labs(y = "log2 fold change", x = NULL) +
  GGally::geom_stripped_cols() +
  ggplot2::scale_fill_manual(
    values = c("TRUE" = "blue", "FALSE" = "red"),
    breaks = c(TRUE, FALSE),  # Specify breaks with the new labels
    labels = c("Positive", "Negative")  # Specify the new legend labels
  ) +
  ggplot2::scale_color_manual(values = "#87ceeb") +
      ggplot2::scale_x_discrete(labels = rev(top_30_names[, x_lab])) +
```

```r
  ggplot2::geom_hline(ggplot2::aes(yintercept = 0),
             linetype = 'dashed',
             color = 'black') +
  ggprism::theme_prism() +
  ggplot2::scale_y_continuous(expand = c(-2, 0),
                     guide = "prism_offset_minor") +
      ylim(-4, 4) +
    ggplot2::theme(
    axis.ticks.y = ggplot2::element_blank(),
    axis.line.y = ggplot2::element_blank(),
    axis.line.x = ggplot2::element_line(size = 0.5),
    axis.ticks.x = ggplot2::element_line(size = 0.5),
    panel.grid.major.y = ggplot2::element_blank(),
    panel.grid.major.x = ggplot2::element_blank(),
    axis.text = ggplot2::element_text(size = 5, color = "black"),
    #axis.text.y = ggplot2::element_blank(),
    axis.text.x = ggplot2::element_text(
      size = 5,
      color = "black",
      margin = ggplot2::margin(b = 6)
    ),
    axis.title.x =  ggplot2::element_text(
      size = 5,
      color = "black",
      hjust = 0.5
    ), strip.text = ggplot2::element_text(size = 5),
    legend.position = "none",  # This suppresses the legend
  ) +
  ggplot2::coord_flip() +
  ggplot2::facet_grid(. ~ comparation, scales = "free_x", space = "free_x",
                     switch = "y")

ggsave("p_values_bar.pdf", plot = p_values_bar, width = 180, height = 185,
       units = "mm", dpi = 600)
ggsave("p_values_bar.svg", plot = p_values_bar, width = 180, height = 185,
       units = "mm", dpi = 600)

# Create labels for 'A' and 'B'
label_A <- letters[1]
label_B <- letters[2]

# Combine the two plots with the specified layout
combination_bar_plot <- (
  (bar_errorbar + labs(tag = label_A)) /
  (p_values_bar + labs(tag = label_B))
) +
plot_layout(nrow = 2)

ggsave("combinated_figures.pdf", plot = combination_bar_plot,
       width = 180, height = 225,
       units = "mm", dpi = 600)
ggsave("combinated_figures.svg", plot = combination_bar_plot,
       width = 180, height = 225,
```

```
       units = "mm", dpi = 600)
```

The TOP30 (by relative abundance) metabolic pathways and their respective differential abundance analysis are presented in the article.

5.0 Coda4Microbiome

5.1 Measure of Association

5.1.1 Log-ratio exploratory analysis for a continuous variable

The variables composing the log-ratio are indicated and ranked according to their aggregated association with the outcome. The x-axis represents the numerators and the y-axis the denominators.

```
library(coda4microbiome)
library(microbiomeutilities)
set.seed(123)

ps <- readRDS("ps1.dna.genus_n0.rds")
ps <- format_to_besthit(ps)
abundance <- as.data.frame(otu_table(ps))
metadata <- sample_data(ps)

abundance <- as.matrix(abundance)

var_logratios<-explore_logratios(x=abundance, y=metadata$Age, measure = "glm")
```

5.1.1.1 Age

Results

Name of the most important features

```
var_logratios$`name of most important variables`
```

```
##  [1] "ASV77:g__Alloprevotella"         "ASV2:g__Prevotella"
##  [3] "ASV33:g__Peptostreptococcus"     "ASV12:g__Dolosigranulum"
##  [5] "ASV238:g__Selenomonas"           "ASV43:g__Granulicatella"
##  [7] "ASV53:g__Stomatobaculum"         "ASV62:g__Lachnoanaerobaculum"
##  [9] "ASV3:g__Veillonella"             "ASV320:g__Lautropia"
## [11] "ASV1300:g__Bacteroidaceae_[G-1]" "ASV316:g__Bifidobacterium"
## [13] "ASV86:g__Roseomonas"             "ASV99:g__Schaalia"
## [15] "ASV2492:f__Veillonellaceae"
```

The pair of taxa whose log-ratio is more associated with the variable

```
var_logratios$`max log-ratio`
```

```
## [1] "26" "9"
```

```
var_logratios$`names max log-ratio`
```

```
## [1] "ASV62:g__Lachnoanaerobaculum" "ASV12:g__Dolosigranulum"
```

The correlation value between the log-ratios and the variable

```
var_logratios$`association log-ratio with y`[1:15,1:15]
```

```
##              32           2          14          9            58            17
```

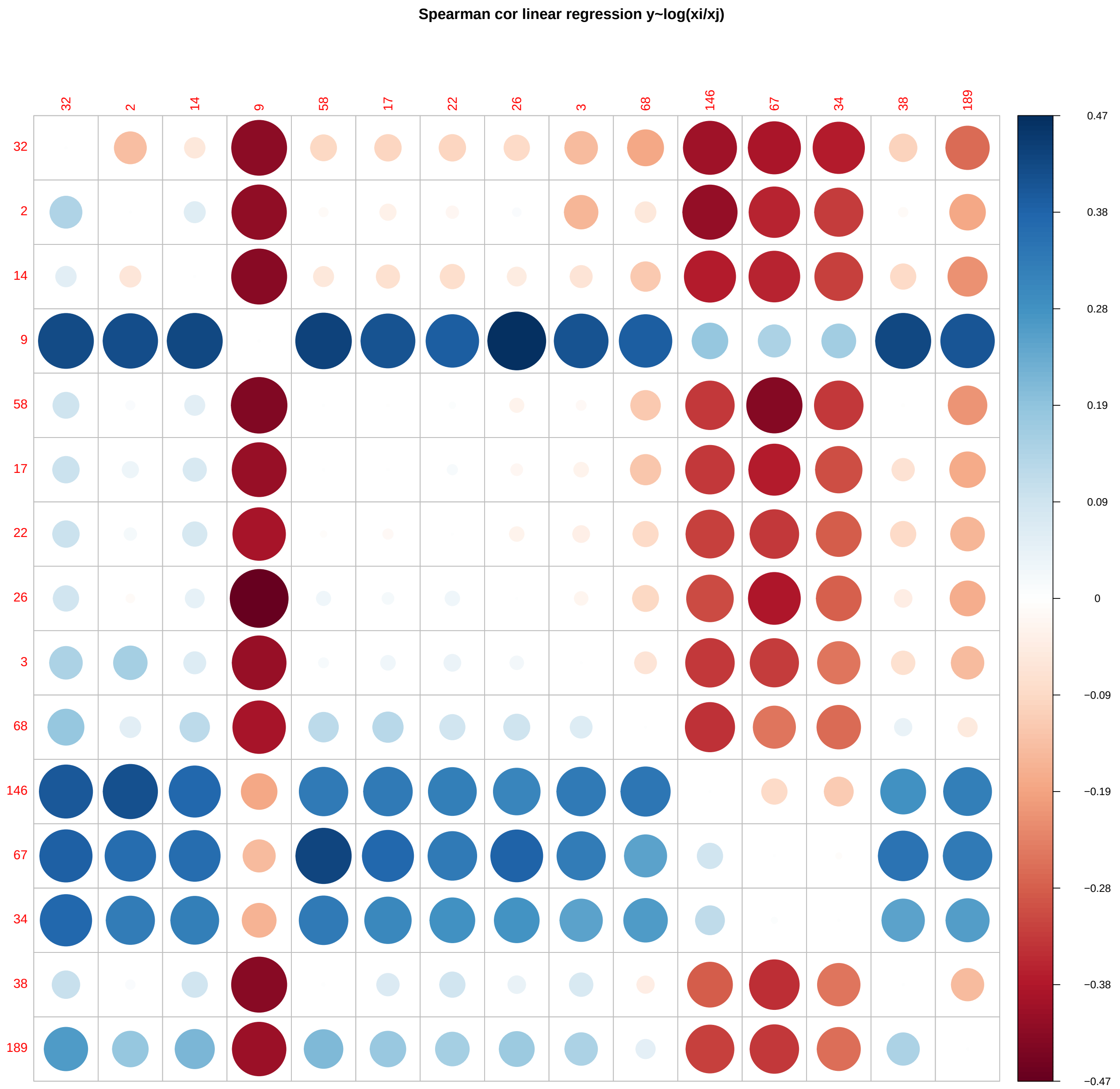

Figure 3: Correlation-like plot of the association of pairwise log-ratios with age.

```
## 32  0.00000000 -0.14444419 -0.05960207 -0.4250458 -0.0953977865 -0.0993192871
## 2   0.14444419  0.00000000  0.06306148 -0.4178835 -0.0119568813 -0.0375914224
## 14  0.05960207 -0.06306148  0.00000000 -0.4272184 -0.0573732617 -0.0767215937
## 9   0.42504577  0.41788351  0.42721836  0.0000000  0.4365333656  0.4098188075
## 58  0.09539779  0.01195688  0.05737326 -0.4365334  0.0000000000 -0.0008730587
## 17  0.09931929  0.03759142  0.07672159 -0.4098188  0.0008730587  0.0000000000
## 22  0.09944582  0.02224359  0.08402803 -0.3893907 -0.0058372093 -0.0150067716
## 26  0.09162055 -0.01078017  0.05086988 -0.4724790  0.0287054385  0.0196691264
## 3   0.15075793  0.15909611  0.06903359 -0.4076980  0.0142600487  0.0307589189
## 68  0.18285987  0.06115091  0.12293049 -0.3892411  0.1238540271  0.1301698930
## 146 0.39789052  0.41507459  0.36940752 -0.1797818  0.3317728322  0.3336391774
## 67  0.38416090  0.36003498  0.36340566 -0.1466237  0.4311909324  0.3689139123
## 34  0.37221240  0.32851689  0.32342990 -0.1624382  0.3334282794  0.3046595217
## 38  0.10723303  0.01276666  0.09099998 -0.4280798  0.0013412375  0.0713119947
## 189 0.26558843  0.17956832  0.21711906 -0.4043709  0.2106676732  0.1788152599
##               22          26           3          68         146           67
## 32  -0.099445817 -0.09162055 -0.15075793 -0.18285987 -0.39789052 -0.384160903
## 2   -0.022243595  0.01078017 -0.15909611 -0.06115091 -0.41507459 -0.360034980
## 14  -0.084028028 -0.05086988 -0.06903359 -0.12293049 -0.36940752 -0.363405657
## 9    0.389390669  0.47247905  0.40769802  0.38924114  0.17978179  0.146623731
## 58   0.005837209 -0.02870544 -0.01426005 -0.12385403 -0.33177283 -0.431190932
## 17   0.015006772 -0.01966913 -0.03075892 -0.13016989 -0.33363918 -0.368913912
## 22   0.000000000 -0.03010470 -0.04028647 -0.09024586 -0.32352601 -0.333732939
## 26   0.030104695  0.00000000 -0.02634310 -0.09615969 -0.30741145 -0.379596430
## 3    0.040286466  0.02634310  0.00000000 -0.06783833 -0.33305924 -0.329174839
## 68   0.090245859  0.09615969  0.06783833  0.00000000 -0.34211919 -0.251060325
## 146  0.323526009  0.30741145  0.33305924  0.34211919  0.00000000 -0.091235939
## 67   0.333732939  0.37959643  0.32917484  0.25106032  0.09123594  0.000000000
## 34   0.283501768  0.28325060  0.25372156  0.26834100  0.11855061  0.005324161
## 38   0.090853311  0.04428122  0.07825747 -0.04269265 -0.28461800 -0.347680342
## 189  0.160613088  0.17236578  0.14977828  0.05279389 -0.32343223 -0.332444076
##               34           38          189
## 32  -0.372212400 -0.107233026 -0.26558843
## 2   -0.328516894 -0.012766659 -0.17956832
## 14  -0.323429901 -0.090999976 -0.21711906
## 9    0.162438224  0.428079829  0.40437093
## 58  -0.333428279 -0.001341238 -0.21066767
## 17  -0.304659522 -0.071311995 -0.17881526
## 22  -0.283501768 -0.090853311 -0.16061309
## 26  -0.283250604 -0.044281219 -0.17236578
## 3   -0.253721563 -0.078257472 -0.14977828
## 68  -0.268341004  0.042692654 -0.05279389
## 146 -0.118550606  0.284618003  0.32343223
## 67  -0.005324161  0.347680342  0.33244408
## 34   0.000000000  0.254700550  0.26279849
## 38  -0.254700550  0.000000000 -0.14809029
## 189 -0.262798488  0.148090290  0.00000000
```

```r
library(coda4microbiome)
library(microbiomeutilities)
set.seed(123)

ps <- readRDS("ps1.dna.genus_n0.rds")
```

```
ps <- format_to_besthit(ps)
abundance <- as.data.frame(otu_table(ps))
metadata <- sample_data(ps)

#Removing instances with missing values
abundance <- abundance[-c(1,16,63:66,76,78),]
metadata <- metadata[-c(1,16,63:66,76,78),]

abundance <- as.matrix(abundance)

var_logratios<-explore_logratios(x=abundance, y=metadata$ALT, measure = "glm")
```

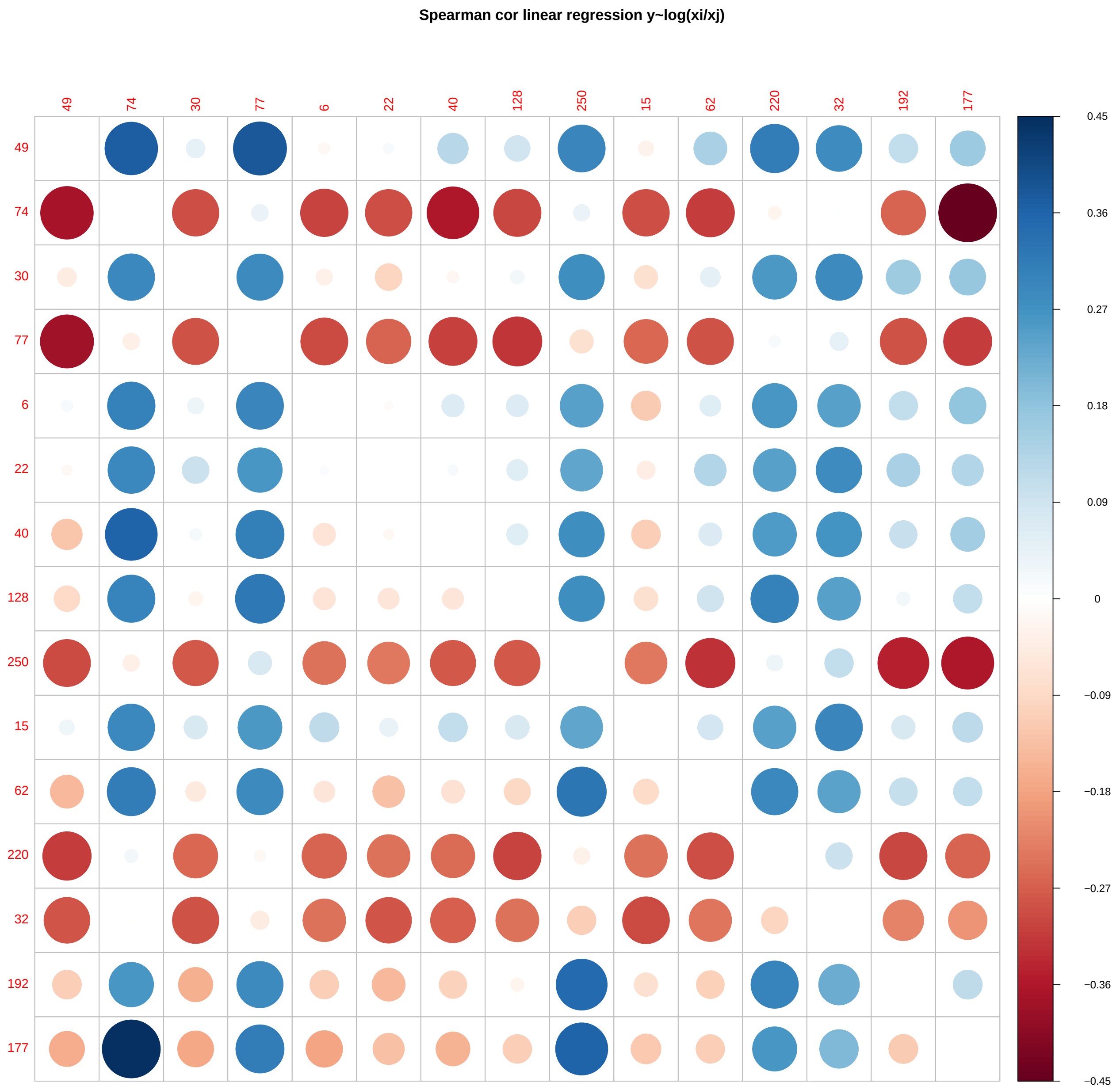

Figure 4: Correlation-like plot of the association of pairwise log-ratios with ALT.

5.1.1.2 Alanine aminotransferase (ALT) Results

Name of the most important variables

```
var_logratios$`name of most important variables`
```

```
##  [1] "ASV169:c__Alphaproteobacteria"  "ASV368:g__Sneathia"
##  [3] "ASV73:g__Oribacterium"          "ASV393:g__Scardovia"
##  [5] "ASV7:g__Agrobacterium"          "ASV53:g__Stomatobaculum"
##  [7] "ASV110:g__Achromobacter"        "ASV901:g__Anaerococcus"
##  [9] "ASV8901:c__Coriobacteriia"      "ASV38:g__Novosphingobium"
## [11] "ASV272:g__Peptidiphaga"         "ASV3813:c__Gammaproteobacteria"
## [13] "ASV77:g__Alloprevotella"        "ASV2538:f__Selenomonadaceae"
## [15] "ASV2111:g__Dietzia"
```

The pair of taxa whose log-ratio is more associated with the variable

```
var_logratios$`max log-ratio`
```

```
## [1] NA NA
```

```
var_logratios$`names max log-ratio`
```

```
## [1] NA NA
```

The correlation value between the log-ratios and the variable

```
var_logratios$`association log-ratio with y`[1:15,1:15]
```

```
##              49          74          30          77            6            22
## 49   0.00000000  0.36795964  0.04731191  0.37526766 -0.017628319  0.014476635
## 74  -0.36795964  0.00000000 -0.28797760  0.03740955 -0.299619356 -0.288279034
## 30  -0.04731191  0.28797760  0.00000000  0.28443772 -0.035697097 -0.095432744
## 77  -0.37526766 -0.03740955 -0.28443772  0.00000000 -0.294013374 -0.263165290
## 6    0.01762832  0.29961936  0.03569710  0.29401337  0.000000000 -0.009572606
## 22  -0.01447664  0.28827903  0.09543274  0.26316529  0.009572606  0.000000000
## 40  -0.12395716  0.35769869  0.01866198  0.30787207 -0.066690263 -0.014003570
## 128 -0.08779350  0.29714279 -0.02590583  0.32002324 -0.064694736 -0.058836943
## 250 -0.29421187 -0.03636233 -0.27395354  0.07289639 -0.244561645 -0.233893457
## 15   0.03065800  0.28841228  0.07268017  0.25616333  0.114216044  0.044286565
## 62  -0.14507411  0.30824673 -0.05286328  0.28494524 -0.058624273 -0.133671617
## 220 -0.31218284  0.02324253 -0.25897047 -0.01760248 -0.263400960 -0.242438995
## 32  -0.27860920 -0.00299320 -0.28582807 -0.04491236 -0.242130149 -0.276919189
## 192 -0.11100842  0.26284351 -0.15649099  0.28508481 -0.109311268 -0.144841419
## 177 -0.16410529  0.44661967 -0.17199220  0.30854524 -0.177217605 -0.130524288
##              40         128         250          15          62         220
## 49   0.12395716  0.08779350  0.29421187 -0.03065800  0.14507411  0.31218284
## 74  -0.35769869 -0.29714279  0.03636233 -0.28841228 -0.30824673 -0.02324253
## 30  -0.01866198  0.02590583  0.27395354 -0.07268017  0.05286328  0.25897047
## 77  -0.30787207 -0.32002324 -0.07289639 -0.25616333 -0.28494524  0.01760248
## 6    0.06669026  0.06469474  0.24456164 -0.11421604  0.05862427  0.26340096
## 22   0.01400357  0.05883694  0.23389346 -0.04428657  0.13367162  0.24243899
## 40   0.00000000  0.06002572  0.27270682 -0.11010926  0.07010317  0.25124513
## 128 -0.06002572  0.00000000  0.27509610 -0.07577244  0.09181791  0.30095109
## 250 -0.27270682 -0.27509610  0.00000000 -0.23344516 -0.32281759  0.03539114
## 15   0.11010926  0.07577244  0.23344516  0.00000000  0.08459491  0.24180454
## 62  -0.07010317 -0.09181791  0.32281759 -0.08459491  0.00000000  0.28647263
## 220 -0.25124513 -0.30095109 -0.03539114 -0.24180454 -0.28647263  0.00000000
```

```
## 32  -0.26719295 -0.24476633 -0.10851356 -0.29176441 -0.23938634 -0.09435168
## 192 -0.10113037 -0.02399632  0.34561364 -0.07371622 -0.10302429  0.29770015
## 177 -0.15315715 -0.10951830  0.36136122 -0.11893467 -0.10848474  0.26016518
##              32         192        177
## 49  0.27860920  0.11100842  0.1641053
## 74  0.00299320 -0.26284351 -0.4466197
## 30  0.28582807  0.15649099  0.1719922
## 77  0.04491236 -0.28508481 -0.3085452
## 6   0.24213015  0.10931127  0.1772176
## 22  0.27691919  0.14484142  0.1305243
## 40  0.26719295  0.10113037  0.1531572
## 128 0.24476633  0.02399632  0.1095183
## 250 0.10851356 -0.34561364 -0.3613612
## 15  0.29176441  0.07371622  0.1189347
## 62  0.23938634  0.10302429  0.1084847
## 220 0.09435168 -0.29770015 -0.2601652
## 32  0.00000000 -0.21994277 -0.1978261
## 192 0.21994277  0.00000000  0.1122540
## 177 0.19782606 -0.11225397  0.0000000
```

```r
library(coda4microbiome)
library(microbiomeutilities)
set.seed(123)

ps <- readRDS("ps1.dna.genus_n0.rds")
ps <- format_to_besthit(ps)
abundance <- as.data.frame(otu_table(ps))
metadata <- sample_data(ps)

#Removing instances with missing values
abundance <- abundance[-c(1,16,37,63:66,76,78),]
metadata <- metadata[-c(1,16,37,63:66,76,78),]

abundance <- as.matrix(abundance)

var_logratios<-explore_logratios(x=abundance, y=metadata$AST, measure = "glm")
```

5.1.1.3 Aspartate aminotransferase (AST) Results

Name of the most important variables

```r
var_logratios$`name of most important variables`
```

```
##  [1] "ASV77:g__Alloprevotella"            "ASV94:g__Campylobacter"
##  [3] "ASV82:g__Atopobium"                 "ASV667:c__Firmicutes"
##  [5] "ASV64:g__Porphyromonas"             "ASV823:g__Peptococcus"
##  [7] "ASV4909:o__Bacteroidales"           "ASV8:g__Rothia"
##  [9] "ASV272:g__Peptidiphaga"             "ASV876:c__Bacteroidetes"
## [11] "ASV660:f__Streptococcaceae"         "ASV2743:g__Simonsiella"
## [13] "ASV7:g__Agrobacterium"              "ASV2229:g__Peptoniphilaceae_[G-1]"
## [15] "ASV10036:o__Campylobacterales"
```

The pair of taxa whose log-ratio is more associated with the variable

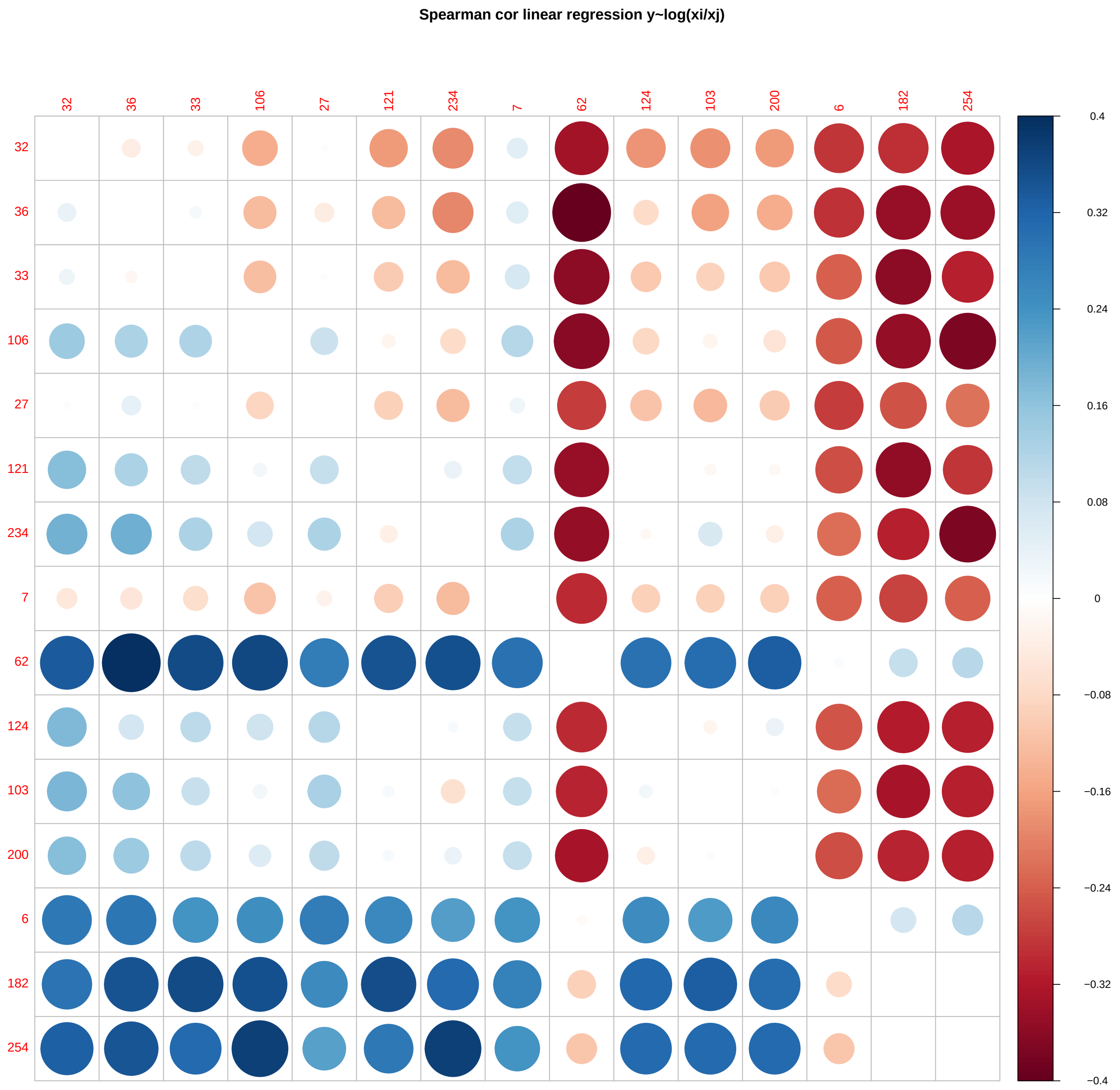

Figure 5: Correlation-like plot of the association of pairwise log-ratios with AST.

```
var_logratios$`max log-ratio`
```

```
## [1] NA NA
```

```
var_logratios$`names max log-ratio`
```

```
## [1] NA NA
```

The correlation value between the log-ratios and the variable

```
var_logratios$`association log-ratio with y`[1:15,1:15]
```

```
##              32          36           33          106            27          121
## 32   0.00000000 -0.03875218 -0.028006215 -0.14468203  0.005665360 -0.168113538
## 36   0.03875218  0.00000000  0.016922670 -0.12409540 -0.043320939 -0.124527027
## 33   0.02800622 -0.01692267  0.000000000 -0.12125809  0.005847954 -0.099887606
## 106  0.14468203  0.12409540  0.121258093  0.00000000  0.086760666 -0.021826558
## 27  -0.00566536  0.04332094 -0.005847954 -0.08676067  0.000000000 -0.093601192
## 121  0.16811354  0.12452703  0.099887606  0.02182656  0.093601192  0.000000000
## 234  0.19130078  0.19257906  0.127638582  0.07355414  0.124681260 -0.035206194
## 7   -0.04871163 -0.05537230 -0.071271283 -0.11499452 -0.027631328 -0.096009581
## 62   0.33497205  0.39913724  0.358513516  0.36009395  0.278060642  0.346923513
## 124  0.17821539  0.07274306  0.105651310  0.08036955  0.112995492  0.001710512
## 103  0.18285073  0.16040029  0.090565180  0.02374164  0.128771959  0.015723037
## 200  0.16876234  0.14544458  0.106032225  0.05719743  0.103022769  0.013743991
## 6    0.28564120  0.28989402  0.239475886  0.24714226  0.277546773  0.258487596
## 182  0.29195681  0.34445288  0.357884362  0.34784437  0.252545472  0.353169409
## 254  0.32513960  0.34193354  0.309465079  0.37244651  0.218537240  0.283907591
##             234          7          62          124         103         200
## 32  -0.19130078 0.04871163 -0.33497205 -0.178215387 -0.18285073 -0.16876234
## 36  -0.19257906 0.05537230 -0.39913724 -0.072743060 -0.16040029 -0.14544458
## 33  -0.12763858 0.07127128 -0.35851352 -0.105651310 -0.09056518 -0.10603223
## 106 -0.07355414 0.11499452 -0.36009395 -0.080369548 -0.02374164 -0.05719743
## 27  -0.12468126 0.02763133 -0.27806064 -0.112995492 -0.12877196 -0.10302277
## 121  0.03520619 0.09600958 -0.34692351 -0.001710512 -0.01572304 -0.01374399
## 234  0.00000000 0.12436152 -0.34958687 -0.011997043  0.06705469 -0.03389924
## 7   -0.12436152 0.00000000 -0.29758034 -0.091803734 -0.09195991 -0.09384224
## 62   0.34958687 0.29758034  0.00000000  0.296694759  0.30663934  0.32857174
## 124  0.01199704 0.09180373 -0.29669476  0.000000000 -0.02060772  0.03587282
## 103 -0.06705469 0.09195991 -0.30663934  0.020607723  0.00000000 -0.00785702
## 200  0.03389924 0.09384224 -0.32857174 -0.035872822  0.00785702  0.00000000
## 6    0.22023207 0.23686039 -0.01126977  0.250498127  0.22417481  0.25635067
## 182  0.31125025 0.26858867 -0.09302108  0.314827495  0.33045535  0.30599062
## 254  0.37201476 0.23744039 -0.10797134  0.307562208  0.30981557  0.30930591
##               6         182         254
## 32  -0.28564120 -0.29195681 -0.32513960
## 36  -0.28989402 -0.34445288 -0.34193354
## 33  -0.23947589 -0.35788436 -0.30946508
## 106 -0.24714226 -0.34784437 -0.37244651
## 27  -0.27754677 -0.25254547 -0.21853724
## 121 -0.25848760 -0.35316941 -0.28390759
## 234 -0.22023207 -0.31125025 -0.37201476
## 7   -0.23686039 -0.26858867 -0.23744039
## 62   0.01126977  0.09302108  0.10797134
## 124 -0.25049813 -0.31482750 -0.30756221
## 103 -0.22417481 -0.33045535 -0.30981557
```

```
## 200 -0.25635067 -0.30599062 -0.30930591
## 6    0.00000000  0.07472748  0.10917462
## 182 -0.07472748  0.00000000  0.00369461
## 254 -0.10917462 -0.00369461  0.00000000
```

```r
library(coda4microbiome)
library(microbiomeutilities)
set.seed(123)

ps <- readRDS("ps1.dna.genus_n0.rds")
ps <- format_to_besthit(ps)
abundance <- as.data.frame(otu_table(ps))
metadata <- sample_data(ps)

#Removing instances with missing values
abundance <- abundance[-c(4,65,73,76,78),]
metadata <- metadata[-c(4,65,73,76,78),]

abundance <- as.matrix(abundance)

var_logratios<-
  explore_logratios(x=abundance, y=metadata$C.reactive.protein, measure = "glm")
```

5.1.1.4 C-reactive protein (CRP)   Results

Name of the most important variables

```r
var_logratios$`name of most important variables`
```

```
##  [1] "ASV27:g__Haemophilus"
##  [2] "ASV44:g__Gemella"
##  [3] "ASV215:g__Bergeyella"
##  [4] "ASV11:g__Neisseria"
##  [5] "ASV43:g__Granulicatella"
##  [6] "ASV38:g__Novosphingobium"
##  [7] "ASV203:g__Mycobacterium"
##  [8] "ASV130:g__Absconditabacteria_(SR1)_[G-1]"
##  [9] "ASV1207:f__Neisseriaceae"
## [10] "ASV212:g__Saccharibacteria_(TM7)_[G-3]"
## [11] "ASV136:g__Mesorhizobium"
## [12] "ASV1181:g__Cryptobacterium"
## [13] "ASV7:g__Agrobacterium"
## [14] "ASV52:g__Lactobacillus"
## [15] "ASV260:g__Sphingomonas"
```

The pair of taxa whose log-ratio is more associated with the variable

```r
var_logratios$`max log-ratio`
```

```
## [1] "241" "139"
```

```r
var_logratios$`names max log-ratio`
```

```
## [1] "ASV6205:f__Erysipelotrichaceae" "ASV1181:g__Cryptobacterium"
```

The correlation value between the log-ratios and the variable

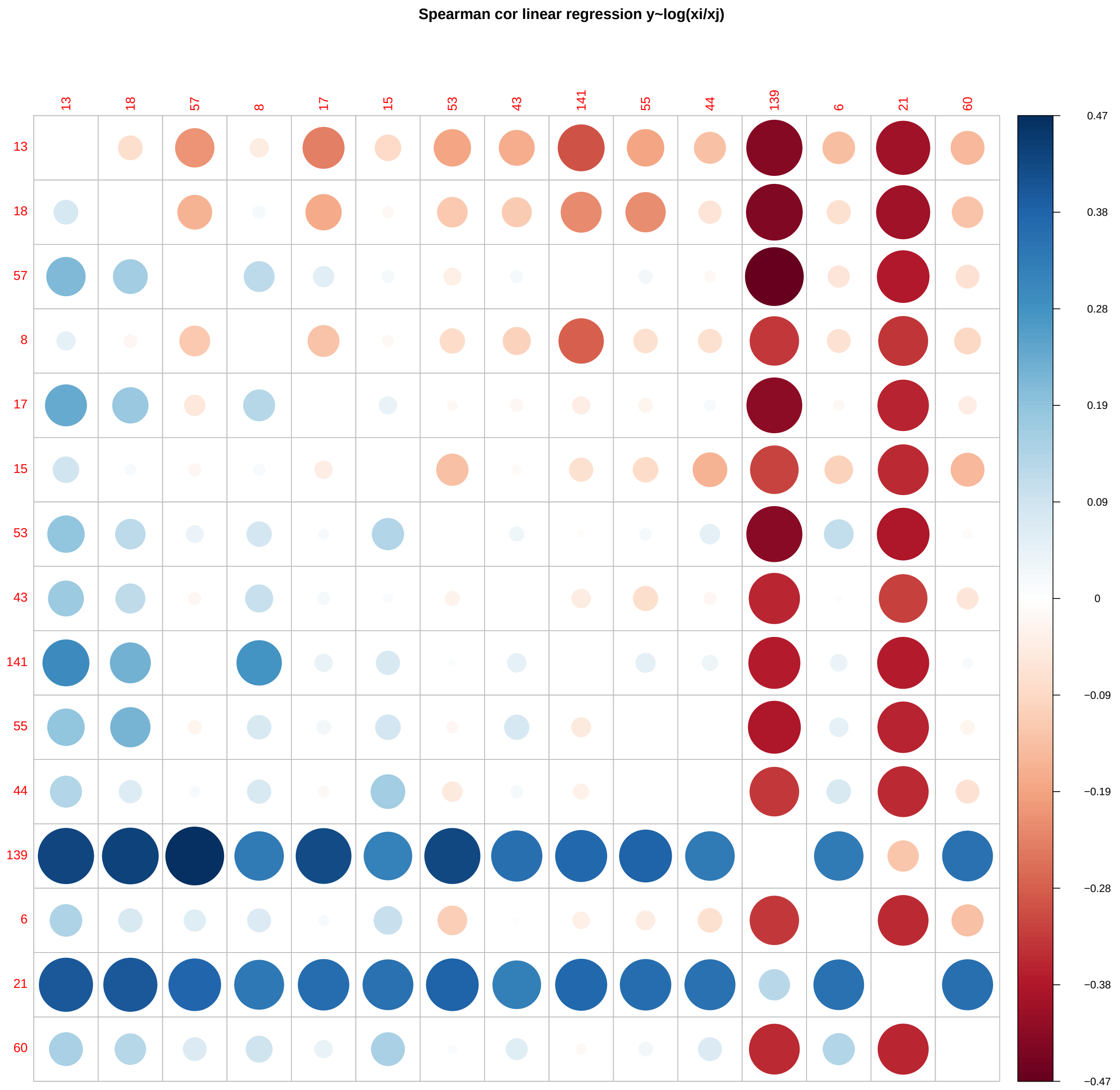

Figure 6: Correlation-like plot of the association of pairwise log-ratios with CRP.

```r
var_logratios$`association log-ratio with y`[1:15,1:15]
```

```
##             13          18           57          8          17          15
## 13  0.00000000 -0.08067266 -0.208398902 -0.04847232 -0.23785135 -0.09305878
## 18  0.08067266  0.00000000 -0.162451980  0.02289399 -0.17707498 -0.01705668
## 57  0.20839890  0.16245198  0.000000000  0.12693613  0.05939296  0.02075285
## 8   0.04847232 -0.02289399 -0.126936131  0.00000000 -0.13615328 -0.01868279
## 17  0.23785135  0.17707498 -0.059392958  0.13615328  0.00000000  0.04330454
## 15  0.09305878  0.01705668 -0.020752853  0.01868279 -0.04330454  0.00000000
## 53  0.18783508  0.12447916  0.041759036  0.08507618  0.01477893  0.13960660
## 43  0.17292277  0.12054262 -0.021184497  0.10519738  0.02224674  0.01314832
## 141 0.29845612  0.22612454 -0.002470624  0.27906368  0.04282563  0.07700941
## 55  0.18853048  0.21869025 -0.025472045  0.07955939  0.02770750  0.08620461
## 44  0.13694742  0.07068790  0.017040458  0.07740671 -0.01752478  0.16120024
## 139 0.43026120  0.43853090  0.471687215  0.33195514  0.42402784  0.32006131
## 6   0.14232158  0.07745012  0.064765979  0.07537681  0.01531864  0.10836213
## 21  0.39928421  0.39847583  0.377009474  0.33845489  0.36011632  0.35059861
## 60  0.15366815  0.13249917  0.074963605  0.09595582  0.04320964  0.15317185
##               53           43          141           55           44        139
## 13  -0.187835076 -0.172922767 -0.298456117 -0.188530478 -0.136947417 -0.4302612
## 18  -0.124479161 -0.120542622 -0.226124537 -0.218690245 -0.070687902 -0.4385309
## 57  -0.041759036  0.021184497  0.002470624  0.025472045 -0.017040458 -0.4716872
## 8   -0.085076183 -0.105197382 -0.279063678 -0.079559392 -0.077406714 -0.3319551
## 17  -0.014778933 -0.022246735 -0.042825628 -0.027707498  0.017524779 -0.4240278
## 15  -0.139606597 -0.013148319 -0.077009405 -0.086204614 -0.161200237 -0.3200613
## 53   0.000000000  0.029382061 -0.007120191  0.019407977  0.054275598 -0.4283235
## 43  -0.029382061  0.000000000 -0.049282801 -0.083928902 -0.020911605 -0.3565987
## 141  0.007120191  0.049282801  0.000000000  0.052240642  0.033599831 -0.3679730
## 55  -0.019407977  0.083928902 -0.052240642  0.000000000  0.003700748 -0.3806021
## 44  -0.054275598  0.020911605 -0.033599831 -0.003700748  0.000000000 -0.3340227
## 139  0.428323507  0.356598729  0.367973011  0.380602098  0.334022655  0.0000000
## 6   -0.117189751  0.006322657 -0.039196021 -0.049541247 -0.079802077 -0.3315400
## 21   0.377697728  0.321150720  0.368585888  0.360398477  0.353354113  0.1312228
## 60   0.011958368  0.063113514 -0.014536140  0.027182768  0.074997055 -0.3505505
##                6         21          60
## 13  -0.142321578 -0.3992842 -0.15366815
## 18  -0.077450122 -0.3984758 -0.13249917
## 57  -0.064765979 -0.3770095 -0.07496361
## 8   -0.075376807 -0.3384549 -0.09595582
## 17  -0.015318637 -0.3601163 -0.04320964
## 15  -0.108362133 -0.3505986 -0.15317185
## 53   0.117189751 -0.3776977 -0.01195837
## 43  -0.006322657 -0.3211507 -0.06311351
## 141  0.039196021 -0.3685859  0.01453614
## 55   0.049541247 -0.3603985 -0.02718277
## 44   0.079802077 -0.3533541 -0.07499706
## 139  0.331539955 -0.1312228  0.35055046
## 6    0.000000000 -0.3492335 -0.13766927
## 21   0.349233542  0.0000000  0.35424172
## 60   0.137669273 -0.3542417  0.00000000
```

```r
library(coda4microbiome)
```

```r
library(microbiomeutilities)
set.seed(123)

ps <- readRDS("ps1.dna.genus_n0.rds")
ps <- format_to_besthit(ps)
abundance <- as.data.frame(otu_table(ps))
metadata <- sample_data(ps)

#Removing instances with missing values
abundance <- abundance[-c(4,16,29,49,64:71,73,76:78),]
metadata <- metadata[-c(4,16,29,49,64:71,73,76:78),]

abundance <- as.matrix(abundance)

var_logratios<-explore_logratios(x=abundance, y=metadata$CPK, measure = "glm")
```

5.1.1.5 Creatine phosphokinase (CPK) Results

Name of the most important variables

```r
var_logratios$`name of most important variables`
```

```
##  [1] "ASV516:g__Tannerella"
##  [2] "ASV12:g__Dolosigranulum"
##  [3] "ASV212:g__Saccharibacteria_(TM7)_[G-3]"
##  [4] "ASV92:g__Saccharibacteria_(TM7)_[G-1]"
##  [5] "ASV53:g__Stomatobaculum"
##  [6] "ASV73:g__Oribacterium"
##  [7] "ASV741:g__Bulleidia"
##  [8] "ASV275:g__Catonella"
##  [9] "ASV238:g__Selenomonas"
## [10] "ASV389:g__Lactococcus"
## [11] "ASV169:c__Alphaproteobacteria"
## [12] "ASV11251:f__Carnobacteriaceae"
## [13] "ASV2092:g__Centipeda"
## [14] "ASV22:g__Leptotrichia"
## [15] "ASV2538:f__Selenomonadaceae"
```

The pair of taxa whose log-ratio is more associated with the variable

```r
var_logratios$`max log-ratio`
```

```
## [1] NA NA
```

```r
var_logratios$`names max log-ratio`
```

```
## [1] NA NA
```

The correlation value between the log-ratios and the variable

```r
var_logratios$`association log-ratio with y`[1:15,1:15]
```

```
##               91         9          55          35          22           30
## 91   0.00000000 0.4513140 -0.08435362 -0.07682842 -0.01999274  0.106262362
## 9   -0.45131403 0.0000000 -0.44026704 -0.50874139 -0.40629883 -0.376328792
## 55   0.08435362 0.4402670  0.00000000 -0.04519451 -0.02587848  0.118169451
## 35   0.07682842 0.5087414  0.04519451  0.00000000 -0.03834411  0.059706005
## 22   0.01999274 0.4062988  0.02587848  0.03834411  0.00000000  0.202698447
```

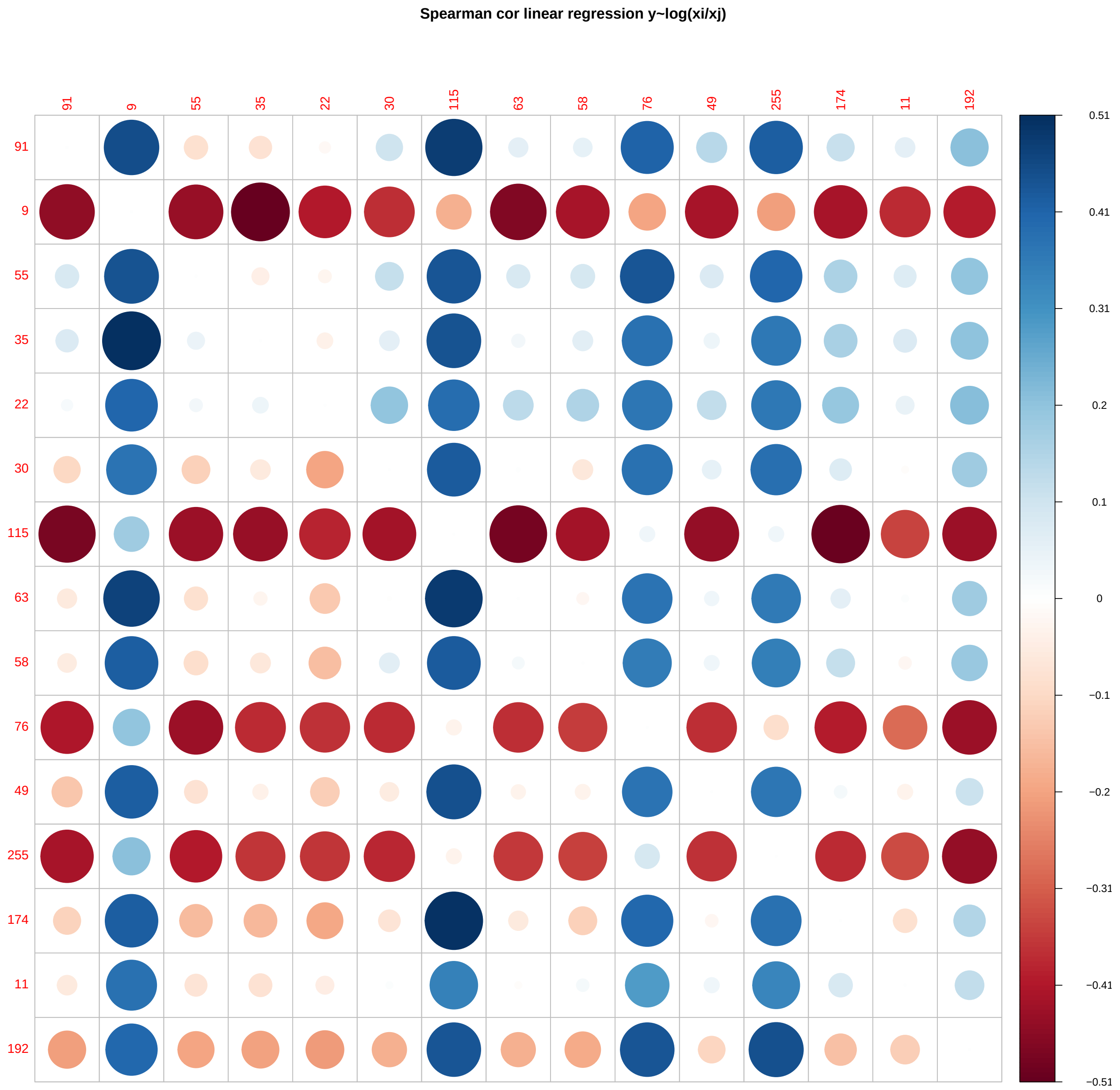

Figure 7: Correlation-like plot of the association of pairwise log-ratios with CPK.

```
## 30  -0.10626236 0.3763288 -0.11816945 -0.05970601 -0.20269845  0.000000000
## 115 -0.48065468 0.1823068 -0.43358972 -0.43880950 -0.38777895 -0.423876349
## 63  -0.05704581 0.4687034 -0.08286907 -0.02725686 -0.13509599 -0.002632772
## 58  -0.05363341 0.4219937 -0.08718624 -0.06113625 -0.15425856  0.061195440
## 76  -0.41188504 0.2032956 -0.43505120 -0.37872649 -0.37102751 -0.381246302
## 49  -0.13827661 0.4193005 -0.08082771 -0.03637880 -0.12549963 -0.053782403
## 255 -0.41740192 0.2110488 -0.40367156 -0.36490537 -0.36530327 -0.385624127
## 174 -0.11285529 0.4198173 -0.16211709 -0.16402572 -0.19687109 -0.071959485
## 11  -0.05943774 0.3804948 -0.07449261 -0.08029473 -0.04996599  0.007633203
## 192 -0.21128495 0.4004313 -0.19900039 -0.20758430 -0.21820150 -0.179607982
##             115          63          58          76          49         255
## 91   0.48065468  0.057045808  0.05363341  0.41188504  0.13827661  0.41740192
## 9   -0.18230683 -0.468703402 -0.42199374 -0.20329556 -0.41930048 -0.21104879
## 55   0.43358972  0.082869066  0.08718624  0.43505120  0.08082771  0.40367156
## 35   0.43880950  0.027256858  0.06113625  0.37872649  0.03637880  0.36490537
## 22   0.38777895  0.135095992  0.15425856  0.37102751  0.12549963  0.36530327
## 30   0.42387635  0.002632772 -0.06119544  0.38124630  0.05378240  0.38562413
## 115  0.00000000 -0.488212777 -0.42366792  0.03532602 -0.44277175  0.03549790
## 63   0.48821278  0.000000000 -0.02230821  0.37344764  0.03200344  0.35894938
## 58   0.42366792  0.022308208  0.00000000  0.35209174  0.03497404  0.34842923
## 76  -0.03532602 -0.373447639 -0.35209174  0.00000000 -0.37329022 -0.09116277
## 49   0.44277175 -0.032003444 -0.03497404  0.37329022  0.00000000  0.37117034
## 255 -0.03549790 -0.358949378 -0.34842923  0.09116277 -0.37117034  0.00000000
## 174  0.50215869 -0.057399542 -0.11950742  0.39893291 -0.02491676  0.37714468
## 11   0.34339376 -0.007909619  0.02434693  0.28865071  0.03560723  0.33421520
## 192  0.43583874 -0.178633011 -0.19231385  0.43667645 -0.10906905  0.44497703
##             174           11         192
## 91   0.11285529  0.059437742  0.2112849
## 9   -0.41981730 -0.380494755 -0.4004313
## 55   0.16211709  0.074492611  0.1990004
## 35   0.16402572  0.080294731  0.2075843
## 22   0.19687109  0.049965993  0.2182015
## 30   0.07195948 -0.007633203  0.1796080
## 115 -0.50215869 -0.343393757 -0.4358387
## 63   0.05739954  0.007909619  0.1786330
## 58   0.11950742 -0.024346928  0.1923139
## 76  -0.39893291 -0.288650713 -0.4366764
## 49   0.02491676 -0.035607225  0.1090690
## 255 -0.37714468 -0.334215201 -0.4449770
## 174  0.00000000 -0.085080926  0.1510995
## 11   0.08508093  0.000000000  0.1260866
## 192 -0.15109954 -0.126086628  0.0000000
```

```r
library(coda4microbiome)
library(microbiomeutilities)
set.seed(123)

ps <- readRDS("ps1.dna.genus_n0.rds")
ps <- format_to_besthit(ps)
abundance <- as.data.frame(otu_table(ps))
metadata <- sample_data(ps)

#Removing instances with missing values
```

```r
abundance <- abundance[-c(1,16,29,62:71,73,74,76:78),]
metadata <- metadata[-c(1,16,29,62:71,73,74,76:78),]

abundance <- as.matrix(abundance)

var_logratios<-explore_logratios(x=abundance, y=metadata$D.dimer, measure = "glm")
```

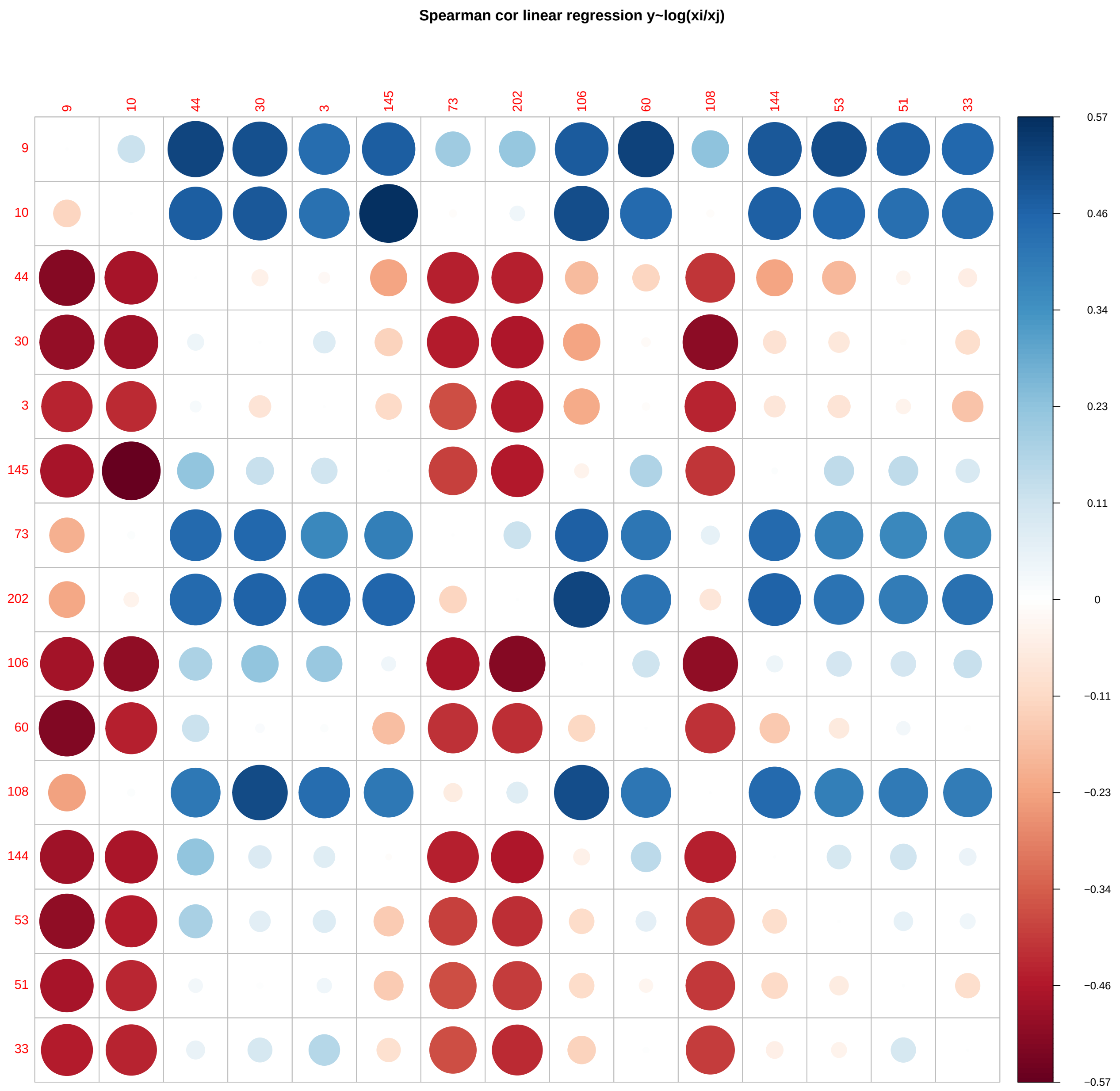

Figure 8: Correlation-like plot of the association of pairwise log-ratios with D-dimer.

5.1.1.6 D-dimer Results

Name of the most important variables

```r
var_logratios$`name of most important variables`
```

```
##  [1] "ASV12:g__Dolosigranulum"      "ASV13:g__Enterococcus"
##  [3] "ASV136:g__Mesorhizobium"      "ASV73:g__Oribacterium"
##  [5] "ASV3:g__Veillonella"          "ASV1294:f__Sphingomonadaceae"
##  [7] "ASV359:g__Aggregatibacter"    "ASV2953:g__Turicella"
##  [9] "ASV667:c__Firmicutes"         "ASV260:g__Sphingomonas"
## [11] "ASV676:g__Eikenella"          "ASV1291:o__Rhizobiales"
## [13] "ASV203:g__Mycobacterium"      "ASV176:g__Megasphaera"
## [15] "ASV82:g__Atopobium"
```

The pair of taxa whose log-ratio is more associated with the variable

```
var_logratios$`max log-ratio`
```

```
## [1] NA NA
```

```
var_logratios$`names max log-ratio`
```

```
## [1] NA NA
```

The correlation value between the log-ratios and the variable

```
var_logratios$`association log-ratio with y`[1:15,1:15]
```

```
##                9           10          44           30            3          145
## 9    0.0000000  0.122600220 0.52115709  0.499749910  0.433700123  0.470202849
## 10  -0.1226002  0.000000000 0.47178873  0.481653352  0.422779816  0.569676012
## 44  -0.5211571 -0.471788732 0.00000000 -0.045071832 -0.021007002 -0.223812176
## 30  -0.4997499 -0.481653352 0.04507183  0.000000000  0.080471268 -0.126658793
## 3   -0.4337001 -0.422779816 0.02100700 -0.080471268  0.000000000 -0.112287429
## 145 -0.4702028 -0.569676012 0.22381218  0.126658793  0.112287429  0.000000000
## 73  -0.2025647  0.008965100 0.44011078  0.447050324  0.365677449  0.388508994
## 202 -0.2184059 -0.037338897 0.44120340  0.456442604  0.448760699  0.454669129
## 106 -0.4751358 -0.504765875 0.18126364  0.227313779  0.213598978  0.034338999
## 60  -0.5251299 -0.443100418 0.12044340  0.013338521  0.009503168 -0.171346042
## 108 -0.2297860  0.009109552 0.40761083  0.508844086  0.435867512  0.407113937
## 144 -0.4820428 -0.463852326 0.22263277  0.087227155  0.075358453 -0.005801538
## 53  -0.5040714 -0.446754916 0.18682100  0.072585800  0.085139491 -0.147350380
## 51  -0.4696955 -0.430425666 0.03240291  0.005460457  0.036150939 -0.143558493
## 33  -0.4446668 -0.433045267 0.05635603  0.099039891  0.160664666 -0.094109964
##              73         202         106           60          108          144
## 9    0.20256472  0.21840586  0.47513582  0.525129943  0.229786039  0.482042820
## 10  -0.00896510  0.03733890  0.50476587  0.443100418 -0.009109552  0.463852326
## 44  -0.44011078 -0.44120340 -0.18126364 -0.120443398 -0.407610832 -0.222632773
## 30  -0.44705032 -0.45644260 -0.22731378 -0.013338521 -0.508844086 -0.087227155
## 3   -0.36567745 -0.44876070 -0.21359898 -0.009503168 -0.435867512 -0.075358453
## 145 -0.38850899 -0.45466913 -0.03433900  0.171346042 -0.407113937  0.005801538
## 73   0.00000000  0.12272958  0.46576239  0.415020575  0.057249648  0.439838251
## 202 -0.12272958  0.00000000  0.52383004  0.417180207 -0.075144186  0.457891345
## 106 -0.46576239 -0.52383004  0.00000000  0.119293078 -0.505286999  0.044574128
## 60  -0.41502057 -0.41718021 -0.11929308  0.000000000 -0.415240294 -0.148927261
## 108 -0.05724965  0.07514419  0.50528700  0.415240294  0.000000000  0.442993034
## 144 -0.43983825 -0.45789135 -0.04457413  0.148927261 -0.442993034  0.000000000
## 53  -0.38756950 -0.41864289 -0.10409893  0.067909649 -0.392206351 -0.097018943
## 51  -0.36626444 -0.39727075 -0.10407259 -0.031210483 -0.403241773 -0.112993810
## 33  -0.36781993 -0.42641787 -0.12991344  0.005433197 -0.394986939 -0.048589690
##              53          51          33
## 9    0.50407142  0.469695512  0.444666750
```

```
## 10   0.44675492  0.430425666  0.433045267
## 44  -0.18682100 -0.032402906 -0.056356032
## 30  -0.07258580 -0.005460457 -0.099039891
## 3   -0.08513949 -0.036150939 -0.160664666
## 145  0.14735038  0.143558493  0.094109964
## 73   0.38756950  0.366264439  0.367819925
## 202  0.41864289  0.397270749  0.426417873
## 106  0.10409893  0.104072587  0.129913437
## 60  -0.06790965  0.031210483 -0.005433197
## 108  0.39220635  0.403241773  0.394986939
## 144  0.09701894  0.112993810  0.048589690
## 53   0.00000000  0.059777026  0.039098538
## 51  -0.05977703  0.000000000 -0.101954900
## 33  -0.03909854  0.101954900  0.000000000
```

```
library(coda4microbiome)
library(microbiomeutilities)
set.seed(123)

ps <- readRDS("ps1.dna.genus_n0.rds")
ps <- format_to_besthit(ps)
abundance <- as.data.frame(otu_table(ps))
metadata <- sample_data(ps)

abundance <- abundance[-c(65,78),]
metadata <- metadata[-c(65,78),]

abundance <- as.matrix(abundance)

var_logratios<-
  explore_logratios(x=abundance, y=metadata$Eosinophils...., measure = "glm")
```

5.1.1.7 Eosinophils (%) Results

Name of the most important variables

```
var_logratios$`name of most important variables`
```

```
##  [1] "ASV24:g__Fusobacterium"       "ASV215:g__Bergeyella"
##  [3] "ASV27:g__Haemophilus"         "ASV44:g__Gemella"
##  [5] "ASV116:g__Acidovorax"         "ASV298:g__Burkholderia"
##  [7] "ASV68:f__Comamonadaceae"      "ASV659:g__Rhodocyclus"
##  [9] "ASV831:c__Proteobacteria"     "ASV162:c__Betaproteobacteria"
## [11] "ASV900:g__Parascardovia"      "ASV876:c__Bacteroidetes"
## [13] "ASV275:g__Catonella"          "ASV346:g__Stenotrophomonas"
## [15] "ASV61:g__Abiotrophia"
```

The pair of taxa whose log-ratio is more associated with the variable

```
var_logratios$`max log-ratio`
```

```
## [1] NA NA
```

```
var_logratios$`names max log-ratio`
```

```
## [1] NA NA
```

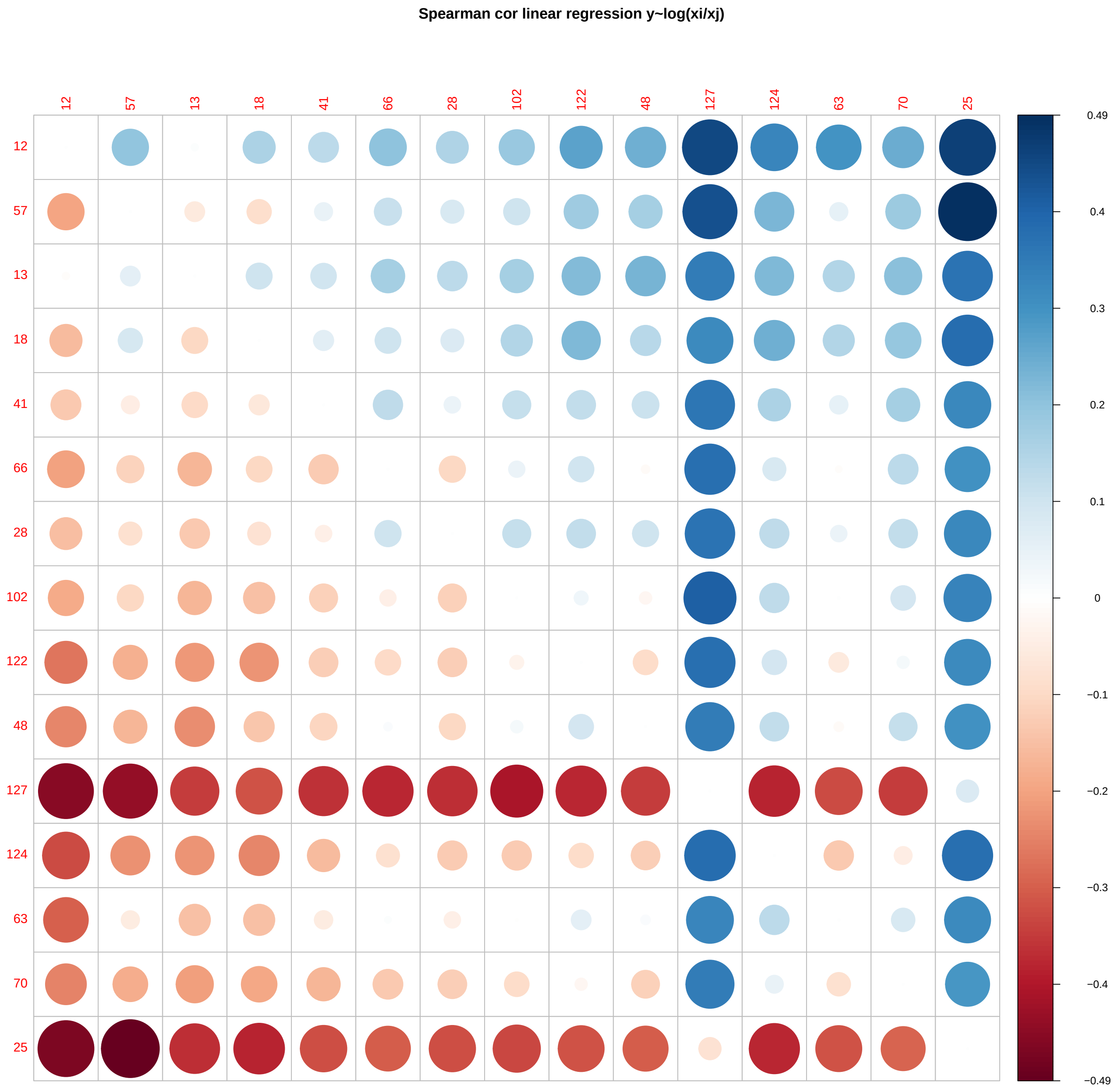

Figure 9: Correlation-like plot of the association of pairwise log-ratios with eosinophils (%).

The correlation value between the log-ratios and the variable

```
var_logratios$`association log-ratio with y`[1:15,1:15]
```

```
##                12          57           13          18          41           66
## 12   0.000000000  0.19576030  0.008291946  0.15368036  0.13350235  0.200720913
## 57  -0.195760302  0.00000000 -0.059173100 -0.08837189  0.04932296  0.110390959
## 13  -0.008291946  0.05917310  0.000000000  0.10092383  0.09782155  0.167007285
## 18  -0.153680360  0.08837189 -0.100923827  0.00000000  0.06127677  0.099112584
## 41  -0.133502347 -0.04932296 -0.097821554 -0.06127677  0.00000000  0.128543081
## 66  -0.200720913 -0.11039096 -0.167007285 -0.09911258 -0.12854308  0.000000000
## 28  -0.151794061 -0.08003744 -0.130655354 -0.07873891 -0.04145639  0.102948655
## 102 -0.184648908 -0.10328290 -0.165043881 -0.14579541 -0.11778164 -0.040381782
## 122 -0.263023420 -0.17317737 -0.216084999 -0.21904316 -0.12338993 -0.096786943
## 48  -0.241028917 -0.16368004 -0.231862449 -0.13571218 -0.10740812  0.011272498
## 127 -0.446265754 -0.43300820 -0.345171015 -0.31276642 -0.35805090 -0.374602455
## 124 -0.323610649 -0.22509219 -0.220662298 -0.23873989 -0.15537624 -0.079794092
## 63  -0.295172217 -0.05011396 -0.145269157 -0.14453654 -0.05125601  0.007181865
## 70  -0.247106326 -0.17910221 -0.205537146 -0.18803173 -0.16462919 -0.132425586
## 25  -0.462496031 -0.49469066 -0.365590803 -0.38021614 -0.31956642 -0.298011290
##               28          102         122         48         127         124
## 12   0.15179406  0.184648908  0.26302342  0.24102892  0.44626575  0.32361065
## 57   0.08003744  0.103282899  0.17317737  0.16368004  0.43300820  0.22509219
## 13   0.13065535  0.165043881  0.21608500  0.23186245  0.34517102  0.22066230
## 18   0.07873891  0.145795408  0.21904316  0.13571218  0.31276642  0.23873989
## 41   0.04145639  0.117781639  0.12338993  0.10740812  0.35805090  0.15537624
## 66  -0.10294865  0.040381782  0.09678694 -0.01127250  0.37460245  0.07979409
## 28   0.00000000  0.117391754  0.12263106  0.10206441  0.36322048  0.12696650
## 102 -0.11739175  0.000000000  0.03004812 -0.02356455  0.40136075  0.12817471
## 122 -0.12263106 -0.030048119  0.00000000 -0.09128308  0.37303903  0.08967796
## 48  -0.10206441  0.023564546  0.09128308  0.00000000  0.34485931  0.12282511
## 127 -0.36322048 -0.401360750 -0.37303903 -0.34485931  0.00000000 -0.37825594
## 124 -0.12696650 -0.128174708 -0.08967796 -0.12282511  0.37825594  0.00000000
## 63  -0.04096785 -0.001250245  0.05888756  0.01439520  0.32534690  0.12914582
## 70  -0.12267010 -0.090724793 -0.02309545 -0.11557878  0.34234630  0.04835368
## 25  -0.31786282 -0.330688316 -0.31233282 -0.30121330 -0.07433286 -0.37345528
##                63          70         25
## 12   0.295172217  0.24710633 0.46249603
## 57   0.050113963  0.17910221 0.49469066
## 13   0.145269157  0.20553715 0.36559080
## 18   0.144536544  0.18803173 0.38021614
## 41   0.051256009  0.16462919 0.31956642
## 66  -0.007181865  0.13242559 0.29801129
## 28   0.040967847  0.12267010 0.31786282
## 102  0.001250245  0.09072479 0.33068832
## 122 -0.058887565  0.02309545 0.31233282
## 48  -0.014395196  0.11557878 0.30121330
## 127 -0.325346905 -0.34234630 0.07433286
## 124 -0.129145821 -0.04835368 0.37345528
## 63   0.000000000  0.08252473 0.31335956
## 70  -0.082524729  0.00000000 0.28757633
## 25  -0.313359559 -0.28757633 0.00000000
```

```r
library(coda4microbiome)
library(microbiomeutilities)
set.seed(123)

ps <- readRDS("ps1.dna.genus_n0.rds")
ps <- format_to_besthit(ps)
abundance <- as.data.frame(otu_table(ps))
metadata <- sample_data(ps)

abundance <- abundance[-c(65,78),]
metadata <- metadata[-c(65,78),]

abundance <- as.matrix(abundance)

var_logratios<-explore_logratios(x=abundance, y=metadata$Erythrocytes, measure = "glm")
```

5.1.1.8 Erytrocytes Results

Name of the most important variables

```r
var_logratios$`name of most important variables`
```

```
##  [1] "ASV465:g__Lawsonella"
##  [2] "ASV38:g__Novosphingobium"
##  [3] "ASV27:g__Haemophilus"
##  [4] "ASV212:g__Saccharibacteria_(TM7)_[G-3]"
##  [5] "ASV64:g__Porphyromonas"
##  [6] "ASV110:g__Achromobacter"
##  [7] "ASV208:g__Cutibacterium"
##  [8] "ASV44:g__Gemella"
##  [9] "ASV33:g__Peptostreptococcus"
## [10] "ASV516:g__Tannerella"
## [11] "ASV92:g__Saccharibacteria_(TM7)_[G-1]"
## [12] "ASV260:g__Sphingomonas"
## [13] "ASV1876:g__Micrococcus"
## [14] "ASV368:g__Sneathia"
## [15] "ASV2684:g__Kluyvera"
```

The pair of taxa whose log-ratio is more associated with the variable

```r
var_logratios$`max log-ratio`
```

```
## [1] "199" "84"
```

```r
var_logratios$`names max log-ratio`
```

```
## [1] "ASV2684:g__Kluyvera"  "ASV465:g__Lawsonella"
```

The correlation value between the log-ratios and the variable

```r
var_logratios$`association log-ratio with y`[1:15,1:15]
```

```
##               84          15          13           55          27          40
## 84  0.0000000000 -0.04217734 -0.08480034 -0.004491549 -0.04647187 -0.01871702
## 15  0.0421773448  0.00000000  0.07043178  0.123831674  0.05999795  0.25560389
## 13  0.0848003391 -0.07043178  0.00000000  0.092428875  0.06811501  0.03658528
## 55  0.0044915487 -0.12383167 -0.09242887  0.000000000 -0.11320484  0.03614488
```

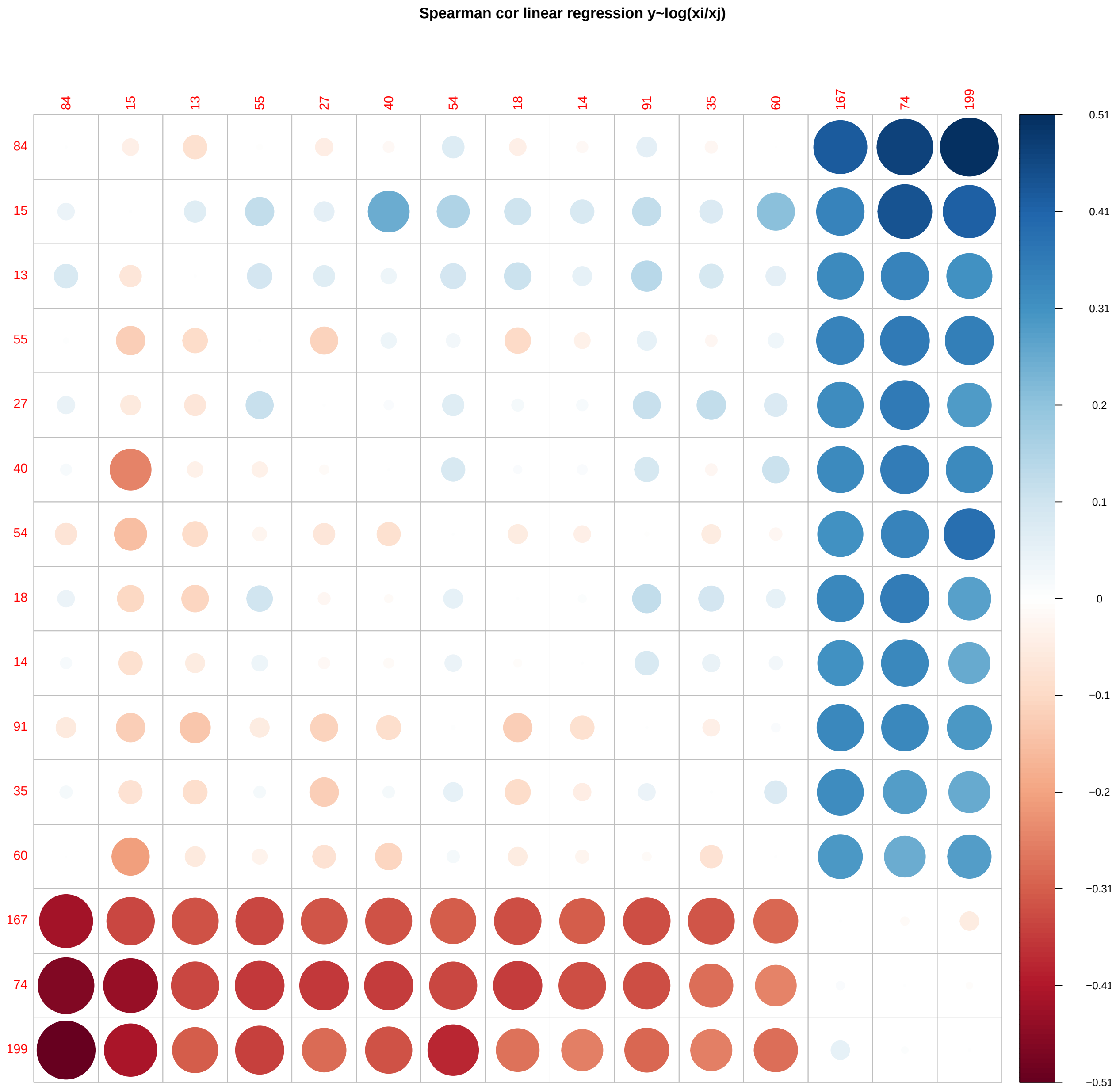

Figure 10: Correlation-like plot of the association of pairwise log-ratios with erytrocytes.

```
## 27   0.0464718730 -0.05999795 -0.06811501  0.113204839  0.00000000  0.01359805
## 40   0.0187170151 -0.25560389 -0.03658528 -0.036144884 -0.01359805  0.00000000
## 54  -0.0719278112 -0.15783649 -0.09465021 -0.029244508 -0.06937238 -0.08272718
## 18   0.0427923783 -0.10565698 -0.10866444  0.099976078 -0.02213137 -0.01045692
## 14   0.0195607446 -0.08379328 -0.05529084  0.038508939 -0.01957901 -0.01521579
## 91  -0.0606651504 -0.12409450 -0.14034008 -0.056042243 -0.11309467 -0.08939685
## 35   0.0233134824 -0.08082220 -0.08761784  0.020967768 -0.12460090  0.02089625
## 60   0.0000685375 -0.21179123 -0.06010227 -0.034667539 -0.07929422 -0.10768149
## 167 -0.4278859737 -0.34269294 -0.32496223 -0.342703007 -0.31737350 -0.32368413
## 74  -0.4724911365 -0.44221196 -0.34109451 -0.362918828 -0.36246155 -0.35409013
## 199 -0.5116327930 -0.41696398 -0.30986427 -0.352459465 -0.29023238 -0.32696184
##               54           18           14           91          35
## 84   0.071927811 -0.042792378 -0.019560745  0.060665150 -0.02331348
## 15   0.157836488  0.105656982  0.083793275  0.124094502  0.08082220
## 13   0.094650206  0.108664443  0.055290844  0.140340083  0.08761784
## 55   0.029244508 -0.099976078 -0.038508939  0.056042243 -0.02096777
## 27   0.069372381  0.022131366  0.019579015  0.113094669  0.12460090
## 40   0.082727184  0.010456918  0.015215790  0.089396853 -0.02089625
## 54   0.000000000 -0.055554417 -0.042555955 -0.002455406 -0.05501733
## 18   0.055554417  0.000000000  0.009987763  0.123556403  0.09666546
## 14   0.042555955 -0.009987763  0.000000000  0.085495102  0.04646758
## 91   0.002455406 -0.123556403 -0.085495102  0.000000000 -0.04379032
## 35   0.055017332 -0.096665460 -0.046467578  0.043790319  0.00000000
## 60   0.023575134 -0.053639794 -0.026947766 -0.012209067 -0.07737559
## 167 -0.310833440 -0.329986958 -0.309647265 -0.330444517 -0.32127382
## 74  -0.339138855 -0.355451206 -0.331936649 -0.327563568 -0.28315459
## 199 -0.388831873 -0.278312396 -0.257509870 -0.294808356 -0.25787364
##                60         167          74         199
## 84  -0.0000685375 0.42788597  0.47249114  0.51163279
## 15   0.2117912296 0.34269294  0.44221196  0.41696398
## 13   0.0601022669 0.32496223  0.34109451  0.30986427
## 55   0.0346675393 0.34270301  0.36291883  0.35245946
## 27   0.0792942206 0.31737350  0.36246155  0.29023238
## 40   0.1076814893 0.32368413  0.35409013  0.32696184
## 54  -0.0235751341 0.31083344  0.33913885  0.38883187
## 18   0.0536397944 0.32998696  0.35545121  0.27831240
## 14   0.0269477656 0.30964727  0.33193665  0.25750987
## 91   0.0122090667 0.33044452  0.32756357  0.29480836
## 35   0.0773755902 0.32127382  0.28315459  0.25787364
## 60   0.0000000000 0.29437777  0.25352289  0.28623838
## 167 -0.2943777704 0.00000000 -0.01080639 -0.05265457
## 74  -0.2535228933 0.01080639  0.00000000 -0.00657650
## 199 -0.2862383768 0.05265457  0.00657650  0.00000000
```

```r
library(coda4microbiome)
library(microbiomeutilities)
set.seed(123)

ps <- readRDS("ps1.dna.genus_n0.rds")
ps <- format_to_besthit(ps)
abundance <- as.data.frame(otu_table(ps))
metadata <- sample_data(ps)
```

```r
abundance <- abundance[-c(65,78),]
metadata <- metadata[-c(65,78),]

abundance <- as.matrix(abundance)

var_logratios<-explore_logratios(x=abundance, y=metadata$Hematocrit, measure = "glm")
```

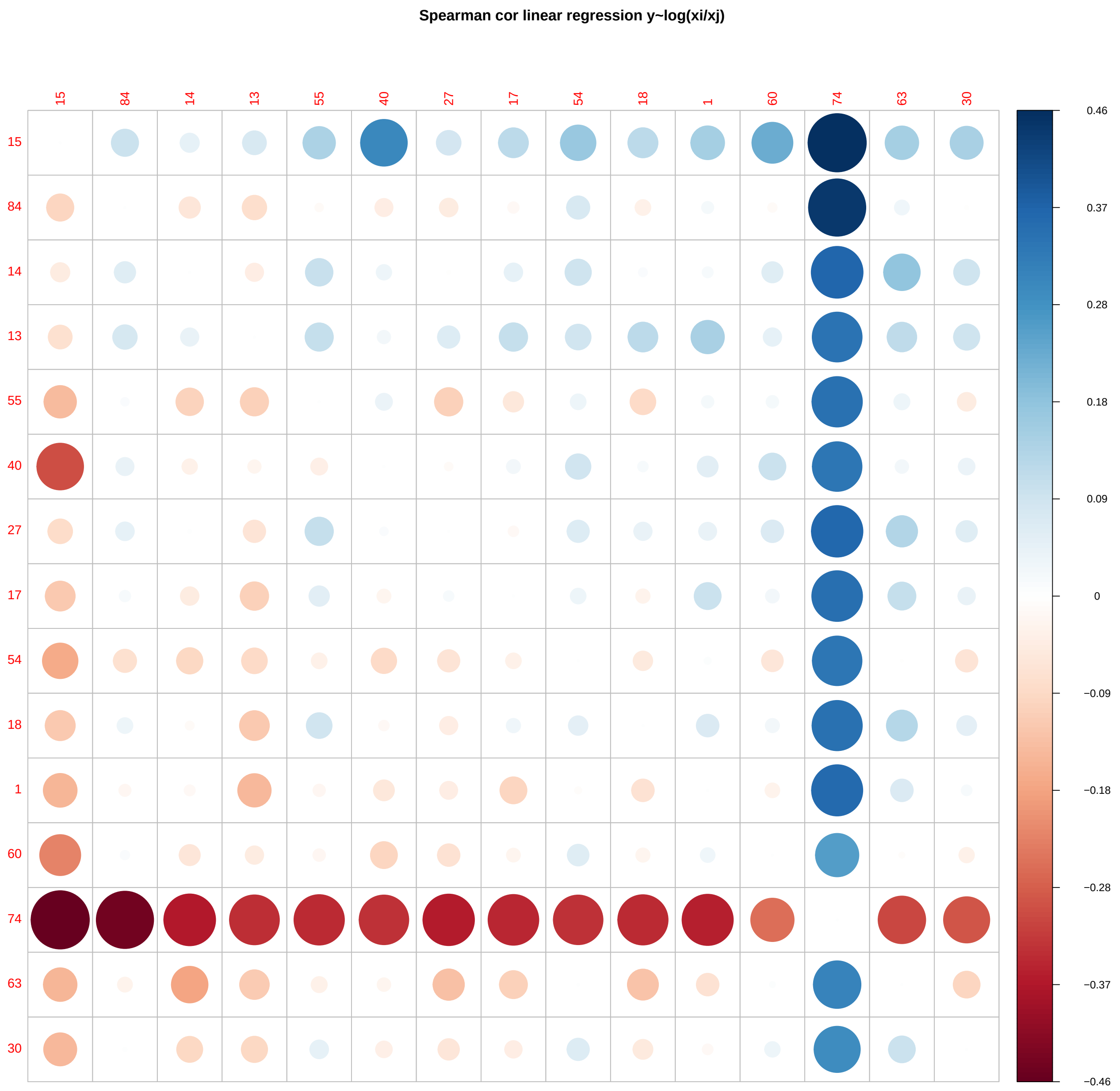

Figure 11: Correlation-like plot of the association of pairwise log-ratios with hematocrit.

5.1.1.9 Hematocrit Results

Name of the most important variables

```r
var_logratios$`name of most important variables`
```

```
##  [1] "ASV38:g__Novosphingobium"
##  [2] "ASV465:g__Lawsonella"
##  [3] "ASV33:g__Peptostreptococcus"
##  [4] "ASV27:g__Haemophilus"
##  [5] "ASV212:g__Saccharibacteria_(TM7)_[G-3]"
##  [6] "ASV110:g__Achromobacter"
##  [7] "ASV64:g__Porphyromonas"
##  [8] "ASV43:g__Granulicatella"
##  [9] "ASV208:g__Cutibacterium"
## [10] "ASV44:g__Gemella"
## [11] "ASV1:g__Streptococcus"
## [12] "ASV260:g__Sphingomonas"
## [13] "ASV368:g__Sneathia"
## [14] "ASV275:g__Catonella"
## [15] "ASV73:g__Oribacterium"
```

The pair of taxa whose log-ratio is more associated with the variable

```
var_logratios$`max log-ratio`
```

```
## [1] "199" "84"
```

```
var_logratios$`names max log-ratio`
```

```
## [1] "ASV2684:g__Kluyvera"  "ASV465:g__Lawsonella"
```

The correlation value between the log-ratios and the variable

```
var_logratios$`association log-ratio with y`[1:15,1:15]
```

```
##              15           84           14          13          55          40
## 15  0.00000000  0.101216538  0.050835675  0.07807757  0.14396031  0.29728619
## 84 -0.10121654  0.000000000 -0.062524461 -0.08206057 -0.01021141 -0.04511213
## 14 -0.05083568  0.062524461  0.000000000 -0.04577585  0.10373058  0.03281314
## 13 -0.07807757  0.082060573  0.045775851  0.00000000  0.10920342  0.02448413
## 55 -0.14396031  0.010211411 -0.103730578 -0.10920342  0.00000000  0.03968863
## 40 -0.29728619  0.045112127 -0.032813140 -0.02448413 -0.03968863  0.00000000
## 27 -0.08354758  0.047888346  0.002031117 -0.06863596  0.10995255  0.01036454
## 17 -0.12249279  0.018027195 -0.047241403 -0.11069031  0.05713817 -0.02686683
## 54 -0.17208301 -0.074176220 -0.094910272 -0.09070090 -0.03416606 -0.08838146
## 18 -0.12371587  0.034293413 -0.011225355 -0.12163557  0.09057888 -0.01542923
## 1  -0.15571658 -0.020241310 -0.016795981 -0.15222236 -0.02120504 -0.05988270
## 60 -0.22881780  0.012172510 -0.061019962 -0.04642435 -0.02174215 -0.09932507
## 74 -0.46214325 -0.446955472 -0.365123418 -0.33892696 -0.34626601 -0.33562955
## 63 -0.15399507 -0.030640964 -0.182585521 -0.12002380 -0.03545036 -0.02539279
## 30 -0.14918536  0.002372779 -0.092515845 -0.09430351  0.04810545 -0.03839737
##               27          17           54          18           1           60
## 15  0.083547576  0.12249279  0.1720830136  0.12371587  0.155716581  0.228817804
## 84 -0.047888346 -0.01802719  0.0741762203 -0.03429341  0.020241310 -0.012172510
## 14 -0.002031117  0.04724140  0.0949102718  0.01122535  0.016795981  0.061019962
## 13  0.068635963  0.11069031  0.0907009019  0.12163557  0.152222360  0.046424348
## 55 -0.109952549 -0.05713817  0.0341660603 -0.09057888  0.021205037  0.021742151
## 40 -0.010364536  0.02686683  0.0883814619  0.01542923  0.059882696  0.099325067
## 27  0.000000000 -0.01552460  0.0681432869  0.04602079  0.044385959  0.069540250
## 17  0.015524596  0.00000000  0.0339134835 -0.02823391  0.099531056  0.026462043
## 54 -0.068143287 -0.03391348  0.0000000000 -0.05218462  0.007013658 -0.064521663
## 18 -0.046020785  0.02823391  0.0521846160  0.00000000  0.070259629  0.027606670
```

```
## 1  -0.044385959 -0.09953106 -0.0070136582 -0.07025963  0.000000000 -0.029818301
## 60 -0.069540250 -0.02646204  0.0645216627 -0.02760667  0.029818301  0.000000000
## 74 -0.361484343 -0.35043103 -0.3352984155 -0.34410178 -0.357076940 -0.256095449
## 63 -0.134320093 -0.10726762  0.0009984477 -0.13131459 -0.070007178  0.004978493
## 30 -0.063000739 -0.04185010  0.0686008437 -0.05448530 -0.016098711  0.032916100
##           74            63           30
## 15 0.4621433  0.1539950659  0.149185361
## 84 0.4469555  0.0306409640 -0.002372779
## 14 0.3651234  0.1825855215  0.092515845
## 13 0.3389270  0.1200237974  0.094303508
## 55 0.3462660  0.0354503566 -0.048105455
## 40 0.3356295  0.0253927922  0.038397375
## 27 0.3614843  0.1343200930  0.063000739
## 17 0.3504310  0.1072676189  0.041850100
## 54 0.3352984 -0.0009984477 -0.068600844
## 18 0.3441018  0.1313145876  0.054485295
## 1  0.3570769  0.0700071780  0.016098711
## 60 0.2560954 -0.0049784929 -0.032916100
## 74 0.0000000 -0.3083837560 -0.290788875
## 63 0.3083838  0.0000000000 -0.098451231
## 30 0.2907889  0.0984512308  0.000000000
```

```
library(coda4microbiome)
library(microbiomeutilities)
set.seed(123)

ps <- readRDS("ps1.dna.genus_n0.rds")
ps <- format_to_besthit(ps)
abundance <- as.data.frame(otu_table(ps))
metadata <- sample_data(ps)

abundance <- abundance[-c(78),]
metadata <- metadata[-c(78),]

abundance <- as.matrix(abundance)

var_logratios<-explore_logratios(x=abundance, y=metadata$Hemoglobin, measure = "glm")
```

5.1.1.10 Hemoglobin   Results

Name of the most important variables

```
var_logratios$`name of most important variables`
```

```
##  [1] "ASV38:g__Novosphingobium"
##  [2] "ASV465:g__Lawsonella"
##  [3] "ASV33:g__Peptostreptococcus"
##  [4] "ASV27:g__Haemophilus"
##  [5] "ASV110:g__Achromobacter"
##  [6] "ASV212:g__Saccharibacteria_(TM7)_[G-3]"
##  [7] "ASV275:g__Catonella"
##  [8] "ASV64:g__Porphyromonas"
##  [9] "ASV260:g__Sphingomonas"
## [10] "ASV208:g__Cutibacterium"
```

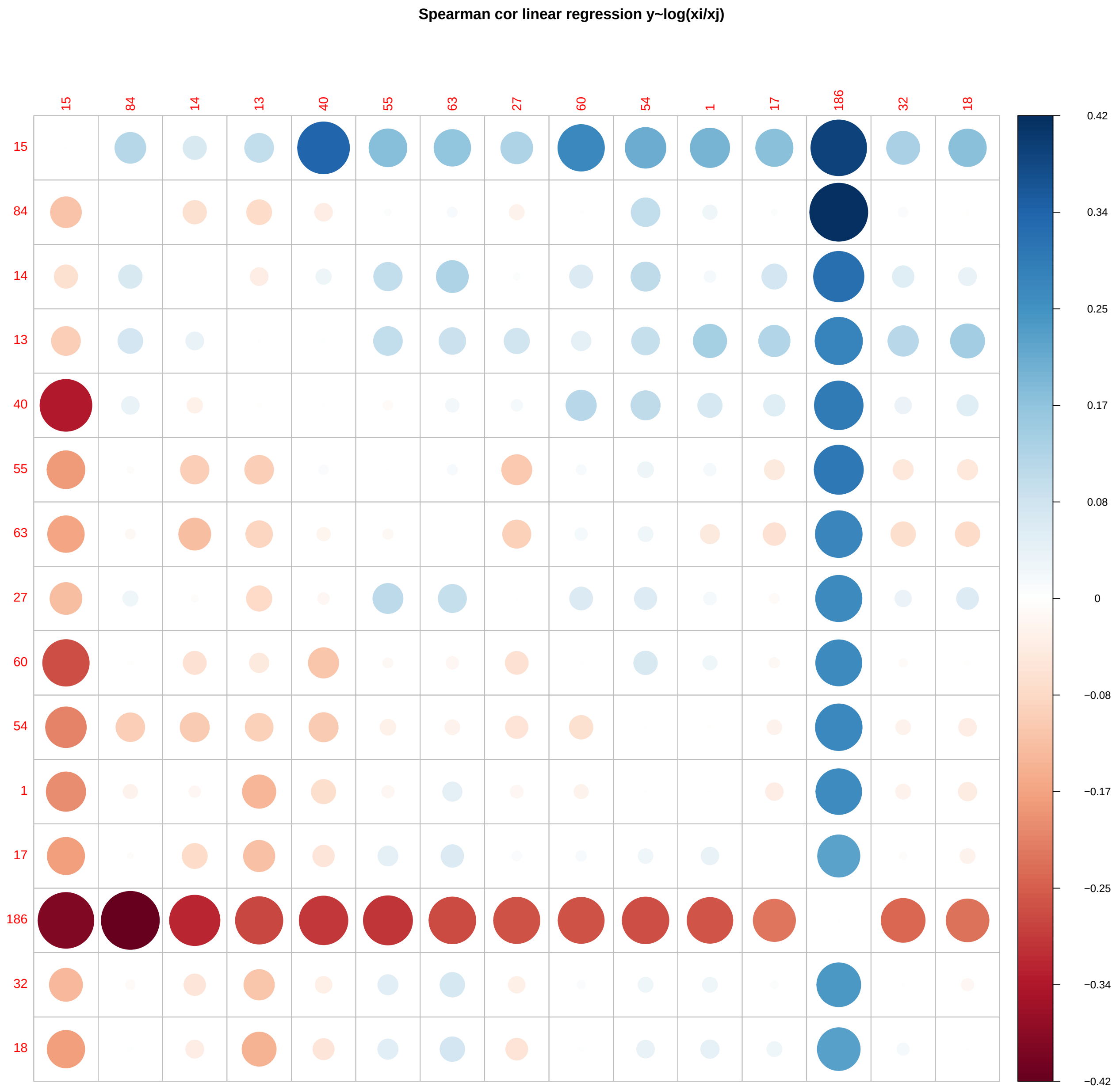

Figure 12: Correlation-like plot of the association of pairwise log-ratios with hemoglobin.

```
## [11] "ASV1:g__Streptococcus"
## [12] "ASV43:g__Granulicatella"
## [13] "ASV2409:g__Pyramidobacter"
## [14] "ASV77:g__Alloprevotella"
## [15] "ASV44:g__Gemella"
```

The pair of taxa whose log-ratio is more associated with the variable

```
var_logratios$`max log-ratio`
```

```
## [1] "199" "84"
```

```
var_logratios$`names max log-ratio`
```

```
## [1] "ASV2684:g__Kluyvera"  "ASV465:g__Lawsonella"
```

The correlation value between the log-ratios and the variable

```
var_logratios$`association log-ratio with y`[1:15,1:15]
```

```
##              15           84           14           13           40          55
## 15   0.00000000  0.119422963  0.068238427  0.104720379  0.335589958  0.17919647
## 84  -0.11942296  0.000000000 -0.069102943 -0.077593866 -0.039619854  0.00544691
## 14  -0.06823843  0.069102943  0.000000000 -0.040126525  0.029812048  0.10125076
## 13  -0.10472038  0.077593866  0.040126525  0.000000000  0.002762268  0.10374176
## 40  -0.33558996  0.039619854 -0.029812048 -0.002762268  0.000000000 -0.01132644
## 55  -0.17919647 -0.005446910 -0.101250759 -0.103741757  0.011326439  0.00000000
## 63  -0.16729566 -0.012659130 -0.128152325 -0.089090158 -0.023039090 -0.01307140
## 27  -0.12749683  0.029119828 -0.006473003 -0.080469529 -0.016967099  0.11324279
## 60  -0.27024622 -0.002017223 -0.067027908 -0.047477516 -0.115220164 -0.01272938
## 54  -0.20689297 -0.103124628 -0.106572156 -0.097090901 -0.106714778 -0.03161929
## 1   -0.19367950 -0.026263187 -0.017017809 -0.138982243 -0.073686365 -0.01908244
## 17  -0.17343444 -0.004557112 -0.078869670 -0.122901515 -0.057954342  0.05002368
## 186 -0.38823162 -0.420130634 -0.317195470 -0.280387547 -0.295957160 -0.30172698
## 32  -0.13691342 -0.011685409 -0.058359752 -0.116081657 -0.035077569  0.05104598
## 18  -0.17577321  0.003117826 -0.040896451 -0.145353284 -0.056319775  0.05156179
##              63           27           60           54            1
## 15   0.16729566  0.127496831  0.270246222  0.206892971  0.193679499
## 84   0.01265913 -0.029119828  0.002017223  0.103124628  0.026263187
## 14   0.12815233  0.006473003  0.067027908  0.106572156  0.017017809
## 13   0.08909016  0.080469529  0.047477516  0.097090901  0.138982243
## 40   0.02303909  0.016967099  0.115220164  0.106714778  0.073686365
## 55   0.01307140 -0.113242794  0.012729383  0.031619285  0.019082441
## 63   0.00000000 -0.099354872  0.019576372  0.027944059 -0.046450464
## 27   0.09935487  0.000000000  0.065826686  0.062779892  0.020871146
## 60  -0.01957637 -0.065826686  0.000000000  0.069597723  0.025789570
## 54  -0.02794406 -0.062779892 -0.069597723  0.000000000 -0.001341426
## 1    0.04645046 -0.020871146 -0.025789570  0.001341426  0.000000000
## 17   0.06394424  0.012362323  0.014124518  0.027144504  0.038835595
## 186 -0.27329473 -0.267641954 -0.264984321 -0.271986789 -0.261579767
## 32   0.07555881 -0.034424487  0.008535375  0.027986378  0.028630237
## 18   0.07570714 -0.060085106  0.004136525  0.039890557  0.042675755
##               17       186           32           18
## 15   0.173434445 0.3882316  0.136913419  0.175773206
## 84   0.004557112 0.4201306  0.011685409 -0.003117826
## 14   0.078869670 0.3171955  0.058359752  0.040896451
## 13   0.122901515 0.2803875  0.116081657  0.145353284
```

```
## 40   0.057954342 0.2959572  0.035077569  0.056319775
## 55  -0.050023678 0.3017270 -0.051045979 -0.051561792
## 63  -0.063944243 0.2732947 -0.075558807 -0.075707144
## 27  -0.012362323 0.2676420  0.034424487  0.060085106
## 60  -0.014124518 0.2649843 -0.008535375 -0.004136525
## 54  -0.027144504 0.2719868 -0.027986378 -0.039890557
## 1   -0.038835595 0.2615798 -0.028630237 -0.042675755
## 17   0.000000000 0.2229741 -0.007285880 -0.028368704
## 186 -0.222974104 0.0000000 -0.240777052 -0.229113758
## 32   0.007285880 0.2407771  0.000000000 -0.018768168
## 18   0.028368704 0.2291138  0.018768168  0.000000000
```

```r
library(coda4microbiome)
library(microbiomeutilities)
set.seed(123)

ps <- readRDS("ps1.dna.genus_n0.rds")
ps <- format_to_besthit(ps)
abundance <- as.data.frame(otu_table(ps))
metadata <- sample_data(ps)

abundance <- abundance[-c(1,4,16,19,25,29,37,63:69,71,73,74,76,78),]
metadata <- metadata[-c(1,4,16,19,25,29,37,63:69,71,73,74,76,78),]

abundance <- as.matrix(abundance)

var_logratios<-explore_logratios(x=abundance, y=metadata$LDH, measure = "glm")
```

5.1.1.11 Lactate dehydrogenase (LDH)   Results

Name of the most important variables

```r
var_logratios$`name of most important variables`
```

```
##  [1] "ASV260:g__Sphingomonas"
##  [2] "ASV297:g__Ruminococcaceae_[G-2]"
##  [3] "ASV27:g__Haemophilus"
##  [4] "ASV44:g__Gemella"
##  [5] "ASV1198:f__Staphylococcaceae"
##  [6] "ASV61:g__Abiotrophia"
##  [7] "ASV24:g__Fusobacterium"
##  [8] "ASV1557:g__Slackia"
##  [9] "ASV212:g__Saccharibacteria_(TM7)_[G-3]"
## [10] "ASV203:g__Mycobacterium"
## [11] "ASV136:g__Mesorhizobium"
## [12] "ASV823:g__Peptococcus"
## [13] "ASV3813:c__Gammaproteobacteria"
## [14] "ASV876:c__Bacteroidetes"
## [15] "ASV375:g__Anoxybacillus"
```

The pair of taxa whose log-ratio is more associated with the variable

```r
var_logratios$`max log-ratio`
```

```
## [1] NA NA
```

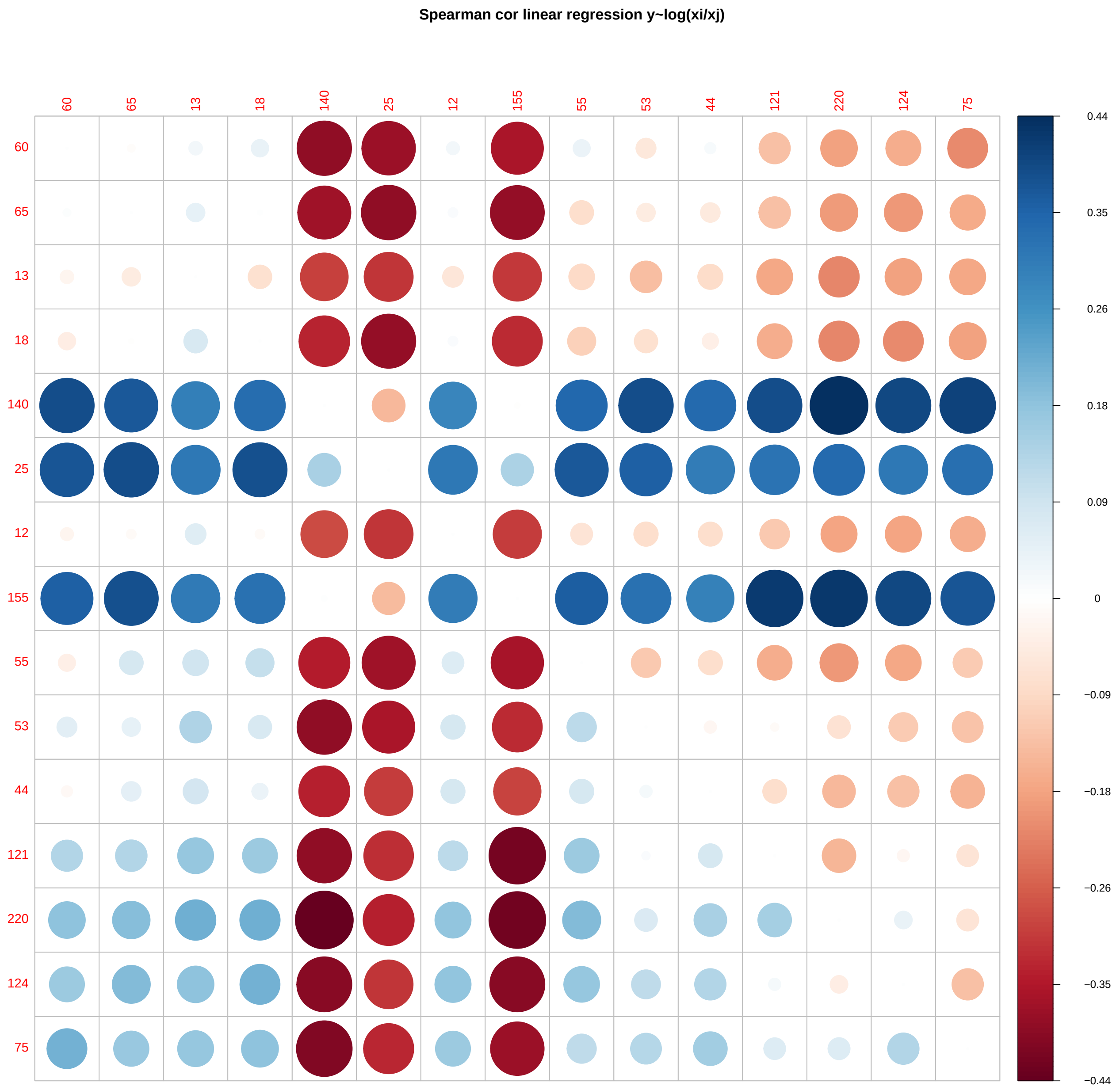

Figure 13: Correlation-like plot of the association of pairwise log-ratios with LDH.

```r
var_logratios$`names max log-ratio`
```

```
## [1] NA NA
```

The correlation value between the log-ratios and the variable

```r
var_logratios$`association log-ratio with y`[1:15,1:15]
```

```
##                60           65          13          18          140         25
## 60   0.000000000 -0.008316511 0.02485607  0.04056819 -0.388935539 -0.3767737
## 65   0.008316511  0.000000000 0.04568439  0.00349405 -0.369716714 -0.3902647
## 13  -0.024856066 -0.045684389 0.00000000 -0.07357068 -0.299367246 -0.3158372
## 18  -0.040568188 -0.003494050 0.07357068  0.00000000 -0.336709493 -0.3852168
## 140  0.388935539  0.369716714 0.29936725  0.33670949  0.000000000 -0.1423558
## 25   0.376773684  0.390264749 0.31583716  0.38521679  0.142355811  0.0000000
## 12  -0.022884700 -0.012743880 0.05744976 -0.01294811 -0.288326346 -0.3136937
## 155  0.356574070  0.382052257 0.30932234  0.32859733  0.004027502 -0.1377318
## 55  -0.039253365  0.075252789 0.08744519  0.10483531 -0.342621541 -0.3709273
## 53   0.053209419  0.045917229 0.13200544  0.07231716 -0.389563804 -0.3568432
## 44  -0.016967092  0.051020981 0.08322495  0.03527314 -0.341230486 -0.3064813
## 121  0.128739871  0.131532068 0.16966074  0.16036786 -0.389444367 -0.3242068
## 220  0.176890308  0.185366743 0.21480464  0.21306708 -0.439027194 -0.3418152
## 124  0.161331816  0.189919774 0.17642847  0.21054247 -0.396605443 -0.3127640
## 75   0.209598079  0.163489996 0.17004268  0.17958200 -0.406595268 -0.3300278
##              12          155          55           53          44          121
## 60   0.02288470 -0.356574070  0.03925337 -0.053209419  0.01696709 -0.128739871
## 65   0.01274388 -0.382052257 -0.07525279 -0.045917229 -0.05102098 -0.131532068
## 13  -0.05744976 -0.309322344 -0.08744519 -0.132005436 -0.08322495 -0.169660742
## 18   0.01294811 -0.328597333 -0.10483531 -0.072317165 -0.03527314 -0.160367858
## 140  0.28832635 -0.004027502  0.34262154  0.389563804  0.34123049  0.389444367
## 25   0.31369369  0.137731847  0.37092727  0.356843178  0.30648133  0.324206786
## 12   0.00000000 -0.306320539 -0.06340715 -0.078213597 -0.07675109 -0.116394308
## 155  0.30632054  0.000000000  0.36027455  0.326986310  0.29431624  0.421003740
## 55   0.06340715 -0.360274551  0.00000000 -0.114305892 -0.07768513 -0.159207164
## 53   0.07821360 -0.326986310  0.11430589  0.000000000 -0.02049247 -0.009657529
## 44   0.07675109 -0.294316235  0.07768513  0.020492473  0.00000000 -0.075127719
## 121  0.11639431 -0.421003740  0.15920716  0.009657529  0.07512772  0.000000000
## 220  0.17142899 -0.422457606  0.19081495  0.068597377  0.14071408  0.148659869
## 124  0.17190788 -0.396688802  0.16772688  0.110578211  0.12992297  0.020144086
## 75   0.16133275 -0.376265897  0.11300973  0.125815304  0.15025051  0.062790916
##             220         124          75
## 60  -0.17689031 -0.16133182 -0.20959808
## 65  -0.18536674 -0.18991977 -0.16349000
## 13  -0.21480464 -0.17642847 -0.17004268
## 18  -0.21306708 -0.21054247 -0.17958200
## 140  0.43902719  0.39660544  0.40659527
## 25   0.34181518  0.31276400  0.33002779
## 12  -0.17142899 -0.17190788 -0.16133275
## 155  0.42245761  0.39668880  0.37626590
## 55  -0.19081495 -0.16772688 -0.11300973
## 53  -0.06859738 -0.11057821 -0.12581530
## 44  -0.14071408 -0.12992297 -0.15025051
## 121 -0.14865987 -0.02014409 -0.06279092
## 220  0.00000000  0.04153317 -0.06412379
## 124 -0.04153317  0.00000000 -0.12999840
```

```
## 75   0.06412379  0.12999840  0.00000000
```

```r
library(coda4microbiome)
library(microbiomeutilities)
set.seed(123)

ps <- readRDS("ps1.dna.genus_n0.rds")
ps <- format_to_besthit(ps)
abundance <- as.data.frame(otu_table(ps))
metadata <- sample_data(ps)

abundance <- abundance[-c(65,78),]
metadata <- metadata[-c(65,78),]

abundance <- as.matrix(abundance)

var_logratios<-explore_logratios(x=abundance, y=metadata$Leukocytes, measure = "glm")
```

5.1.1.12 Leukocytes Results

Name of the most important variables

```r
var_logratios$`name of most important variables`
```

```
##  [1] "ASV12:g__Dolosigranulum"
##  [2] "ASV176:g__Megasphaera"
##  [3] "ASV53:g__Stomatobaculum"
##  [4] "ASV4:g__Corynebacterium"
##  [5] "ASV73:g__Oribacterium"
##  [6] "ASV1213:g__Olsenella"
##  [7] "ASV349:g__Dialister"
##  [8] "ASV153:g__Mycoplasma"
##  [9] "ASV901:g__Anaerococcus"
## [10] "ASV1733:g__Lachnospiraceae_[G-7]"
## [11] "ASV150:g__Parvimonas"
## [12] "ASV810:g__Peptostreptococcaceae_[XI][G-7]"
## [13] "ASV1359:o__Corynebacteriales"
## [14] "ASV297:g__Ruminococcaceae_[G-2]"
## [15] "ASV69:g__Actinomyces"
```

The pair of taxa whose log-ratio is more associated with the variable

```r
var_logratios$`max log-ratio`
```

```
## [1] "241" "139"
```

```r
var_logratios$`names max log-ratio`
```

```
## [1] "ASV6205:f__Erysipelotrichaceae" "ASV1181:g__Cryptobacterium"
```

The correlation value between the log-ratios and the variable

```r
var_logratios$`association log-ratio with y`[1:15,1:15]
```

```
##               9          51          22           4          30          142
## 9    0.00000000  0.346327394 0.345323130  0.09586859  0.37924670  0.155340590
## 51  -0.34632739  0.000000000 0.004772421 -0.31349925 -0.11049739 -0.322519131
```

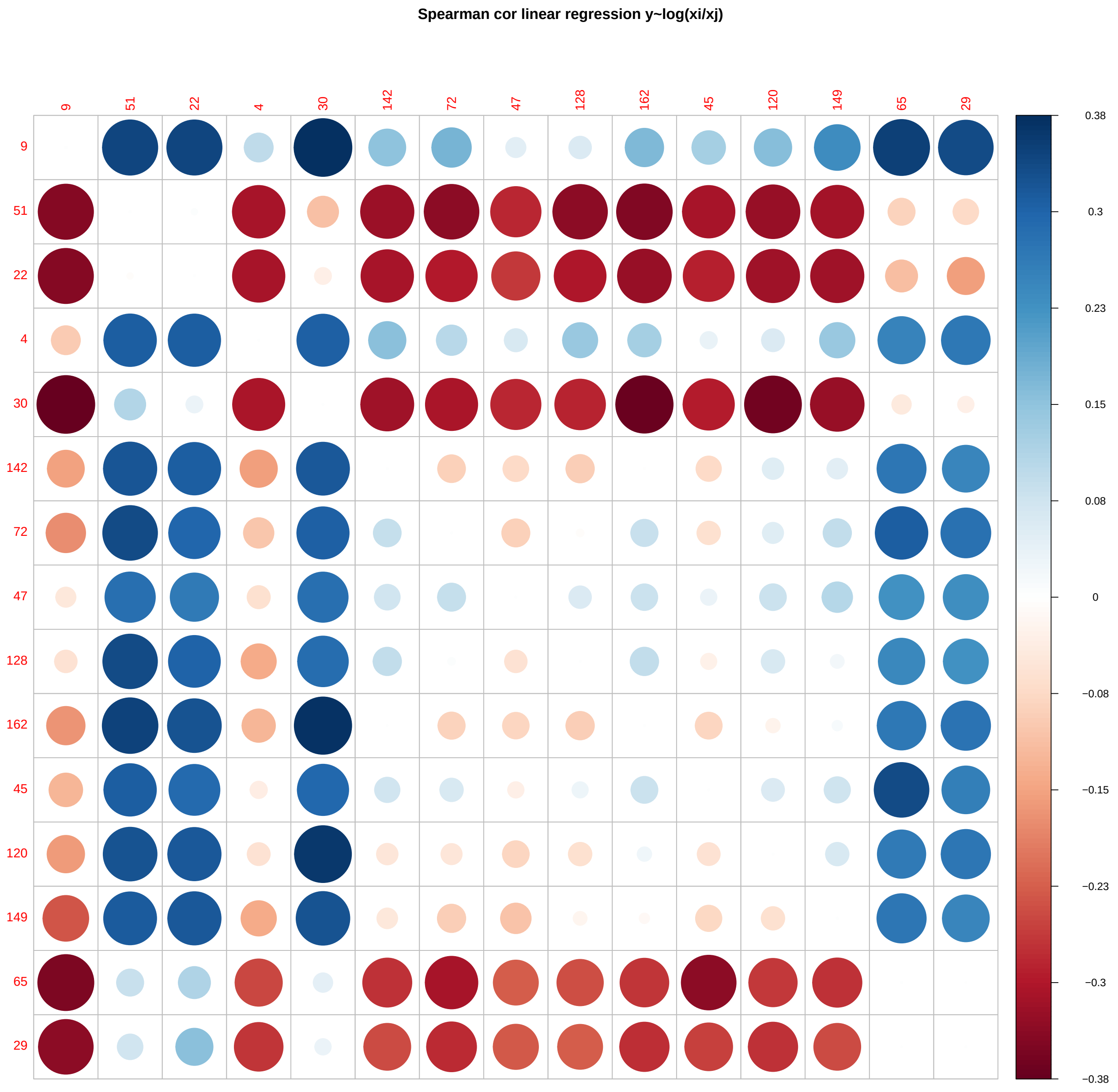

Figure 14: Correlation-like plot of the association of pairwise log-ratios with leukocytes.

```
## 22  -0.34532313 -0.004772421 0.000000000 -0.31162635 -0.03302203 -0.313218064
## 4   -0.09586859  0.313499252 0.311626349  0.00000000  0.30865978  0.158386537
## 30  -0.37924670  0.110497389 0.033022029 -0.30865978  0.00000000 -0.318619042
## 142 -0.15534059  0.322519131 0.313218064 -0.15838654  0.31861904  0.000000000
## 72  -0.17729011  0.339923165 0.300271861 -0.10539663  0.30966119  0.088183912
## 47  -0.04664112  0.288116708 0.265586869 -0.06112349  0.28669378  0.075840301
## 128 -0.05829130  0.338156580 0.305483706 -0.14066099  0.29151912  0.091889813
## 162 -0.16798123  0.351408038 0.328201638 -0.12688149  0.37288299  0.000392965
## 45  -0.12872344  0.312464113 0.295125790 -0.03436915  0.29907373  0.073352860
## 120 -0.15954200  0.328178149 0.322331579 -0.06023446  0.36738574 -0.052902647
## 149 -0.23764712  0.317980541 0.322313685 -0.14215113  0.32675834 -0.049063036
## 65  -0.35631269  0.083729725 0.117300121 -0.25364503  0.04334835 -0.273364872
## 29  -0.33961620  0.075093654 0.157173238 -0.26954756  0.03092424 -0.250076956
##              72           47          128          162          45          120
## 9    0.177290114  0.04664112  0.058291302  0.167981228  0.12872344  0.15954200
## 51  -0.339923165 -0.28811671 -0.338156580 -0.351408038 -0.31246411 -0.32817815
## 22  -0.300271861 -0.26558687 -0.305483706 -0.328201638 -0.29512579 -0.32233158
## 4    0.105396630  0.06112349  0.140660993  0.126881485  0.03436915  0.06023446
## 30  -0.309661191 -0.28669378 -0.291519122 -0.372882992 -0.29907373 -0.36738574
## 142 -0.088183912 -0.07584030 -0.091889813 -0.000392965 -0.07335286  0.05290265
## 72   0.000000000 -0.08993855 -0.007102453  0.084519519 -0.06293258  0.05113183
## 47   0.089938547  0.00000000  0.058096572  0.080797360  0.03072147  0.08160376
## 128  0.007102453 -0.05809657  0.000000000  0.092215585 -0.03017954  0.06308700
## 162 -0.084519519 -0.08079736 -0.092215585  0.000000000 -0.08267420 -0.02395539
## 45   0.062932582 -0.03072147  0.030179544  0.082674201  0.00000000  0.06004613
## 120 -0.051131835 -0.08160376 -0.063087000  0.023955393 -0.06004613  0.00000000
## 149 -0.091595199 -0.10644077 -0.022066103 -0.012579140 -0.07875869 -0.06275646
## 65  -0.313969206 -0.23026076 -0.244609127 -0.269755661 -0.33985790 -0.26584979
## 29  -0.282600416 -0.23221952 -0.229834689 -0.276895643 -0.25975154 -0.27664567
##             149            65            29
## 9    0.23764712  0.3563126933  0.3396161961
## 51  -0.31798054 -0.0837297254 -0.0750936536
## 22  -0.32231368 -0.1173001208 -0.1571732383
## 4    0.14215113  0.2536450323  0.2695475643
## 30  -0.32675834 -0.0433483539 -0.0309242411
## 142  0.04906304  0.2733648716  0.2500769563
## 72   0.09159520  0.3139692057  0.2826004160
## 47   0.10644077  0.2302607581  0.2322195187
## 128  0.02206610  0.2446091271  0.2298346890
## 162  0.01257914  0.2697556610  0.2768956428
## 45   0.07875869  0.3398578998  0.2597515441
## 120  0.06275646  0.2658497903  0.2766456734
## 149  0.00000000  0.2756838029  0.2498376089
## 65  -0.27568380  0.0000000000 -0.0002803726
## 29  -0.24983761  0.0002803726  0.0000000000
```

```r
library(coda4microbiome)
library(microbiomeutilities)
set.seed(123)

ps <- readRDS("ps1.dna.genus_n0.rds")
ps <- format_to_besthit(ps)
abundance <- as.data.frame(otu_table(ps))
```

```r
metadata <- sample_data(ps)

abundance <- abundance[-c(65,78),]
metadata <- metadata[-c(65,78),]

abundance <- as.matrix(abundance)

var_logratios<-
  explore_logratios(x=abundance, y=metadata$Lymphocytes...., measure = "glm")
```

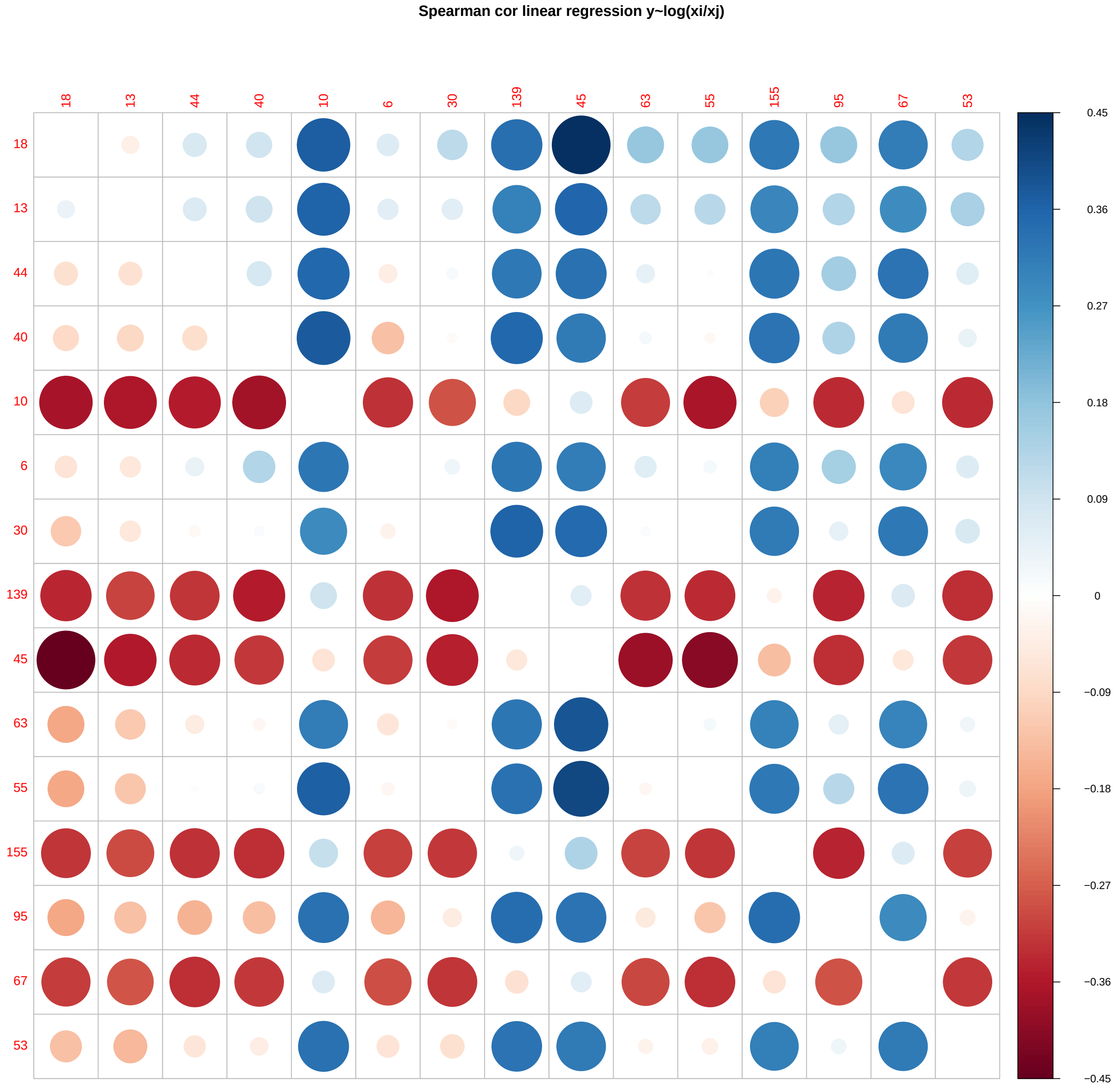

Figure 15: Correlation-like plot of the association of pairwise log-ratios with lymphocytes (%).

5.1.1.13 Lymphocytes (%) Results

Name of the most important variables

```r
var_logratios$`name of most important variables`
```

```
##  [1] "ASV44:g__Gemella"
##  [2] "ASV27:g__Haemophilus"
##  [3] "ASV136:g__Mesorhizobium"
##  [4] "ASV110:g__Achromobacter"
##  [5] "ASV13:g__Enterococcus"
##  [6] "ASV7:g__Agrobacterium"
##  [7] "ASV73:g__Oribacterium"
##  [8] "ASV1181:g__Cryptobacterium"
##  [9] "ASV150:g__Parvimonas"
## [10] "ASV275:g__Catonella"
## [11] "ASV212:g__Saccharibacteria_(TM7)_[G-3]"
## [12] "ASV1557:g__Slackia"
## [13] "ASV543:g__Afipia"
## [14] "ASV316:g__Bifidobacterium"
## [15] "ASV203:g__Mycobacterium"
```

The pair of taxa whose log-ratio is more associated with the variable

```r
var_logratios$`max log-ratio`
```

```
## [1] "45" "18"
```

```r
var_logratios$`names max log-ratio`
```

```
## [1] "ASV150:g__Parvimonas" "ASV44:g__Gemella"
```

The correlation value between the log-ratios and the variable

```r
var_logratios$`association log-ratio with y`[1:15,1:15]
```

```
##              18          13           44          40          10           6
## 18   0.00000000 -0.04008970  0.072991953  0.08574360  0.36863846  0.06311413
## 13   0.04008970  0.00000000  0.071049645  0.09082642  0.36107826  0.05626466
## 44  -0.07299195 -0.07104965  0.000000000  0.07896313  0.34972158 -0.04460796
## 40  -0.08574360 -0.09082642 -0.078963131  0.00000000  0.37325124 -0.13351798
## 10  -0.36863846 -0.36107826 -0.349721576 -0.37325124  0.00000000 -0.32506713
## 6   -0.06311413 -0.05626466  0.044607961  0.13351798  0.32506713  0.00000000
## 30  -0.11733902 -0.05695164 -0.017240908  0.01321628  0.28527677 -0.02937457
## 139 -0.33885812 -0.30347851 -0.317496322 -0.34993411  0.09000829 -0.32429217
## 45  -0.44684227 -0.35443557 -0.334072195 -0.31407566 -0.06454381 -0.31054239
## 63  -0.17376290 -0.11692024 -0.044748152 -0.01906700  0.30861605 -0.06011542
## 55  -0.17193072 -0.12134100 -0.005932775  0.01504052  0.36348406 -0.02112224
## 155 -0.31933694 -0.29403078 -0.322038896 -0.32807034  0.10612353 -0.30510760
## 95  -0.17373913 -0.13067556 -0.153148967 -0.13567345  0.33311056 -0.14812828
## 67  -0.31070909 -0.28021358 -0.329929016 -0.31600425  0.06479916 -0.28662332
## 53  -0.13009376 -0.14643267 -0.061593982 -0.04222731  0.33422877 -0.06510580
##               30         139         45          63           55         155
## 18   0.117339021  0.33885812 0.44684227  0.17376290  0.171930721  0.31933694
## 13   0.056951642  0.30347851 0.35443557  0.11692024  0.121341002  0.29403078
## 44   0.017240908  0.31749632 0.33407219  0.04474815  0.005932775  0.32203890
## 40  -0.013216281  0.34993411 0.31407566  0.01906700 -0.015040518  0.32807034
## 10  -0.285276766 -0.09000829 0.06454381 -0.30861605 -0.363484064 -0.10612353
## 6    0.029374569  0.32429217 0.31054239  0.06011542  0.021122244  0.30510760
## 30   0.000000000  0.35899348 0.34560719  0.01255436  0.000369203  0.31343948
## 139 -0.358993475  0.00000000 0.05457740 -0.32346901 -0.332032615 -0.02735679
```

```
## 45  -0.345607187 -0.05457740 0.00000000 -0.38056515 -0.403426266 -0.13631958
## 63  -0.012554361  0.32346901 0.38056515  0.00000000  0.019008844  0.30288687
## 55  -0.000369203  0.33203261 0.40342627 -0.01900884  0.000000000  0.32128655
## 155 -0.313439485  0.02735679 0.13631958 -0.30288687 -0.321286551  0.00000000
## 95  -0.045482513  0.34197889 0.32737626 -0.04994426 -0.122452927  0.34045142
## 67  -0.319139336 -0.06875510 0.05376987 -0.29601553 -0.329963773 -0.06574977
## 53  -0.075688795  0.32961634 0.31611871 -0.02817435 -0.034443713  0.30557349
##              95          67          53
## 18   0.17373913  0.31070909  0.13009376
## 13   0.13067556  0.28021358  0.14643267
## 44   0.15314897  0.32992902  0.06159398
## 40   0.13567345  0.31600425  0.04222731
## 10  -0.33311056 -0.06479916 -0.33422877
## 6    0.14812828  0.28662332  0.06510580
## 30   0.04548251  0.31913934  0.07568879
## 139 -0.34197889  0.06875510 -0.32961634
## 45  -0.32737626 -0.05376987 -0.31611871
## 63   0.04994426  0.29601553  0.02817435
## 55   0.12245293  0.32996377  0.03444371
## 155 -0.34045142  0.06574977 -0.30557349
## 95   0.00000000  0.28530959 -0.03037555
## 67  -0.28530959  0.00000000 -0.31334750
## 53   0.03037555  0.31334750  0.00000000
```

```r
library(coda4microbiome)
library(microbiomeutilities)
set.seed(123)

ps <- readRDS("ps1.dna.genus_n0.rds")
ps <- format_to_besthit(ps)
abundance <- as.data.frame(otu_table(ps))
metadata <- sample_data(ps)

abundance <- abundance[-c(65,78),]
metadata <- metadata[-c(65,78),]

abundance <- as.matrix(abundance)

var_logratios<-explore_logratios(x=abundance, y=metadata$MCH, measure = "glm")
```

5.1.1.14 Mean Corpuscular Hemoglobin (MCH) Results

Name of the most important variables

```r
var_logratios$`name of most important variables`
```

```
##  [1] "ASV62:g__Lachnoanaerobaculum" "ASV69:g__Actinomyces"
##  [3] "ASV3:g__Veillonella"          "ASV82:g__Atopobium"
##  [5] "ASV8:g__Rothia"               "ASV1:g__Streptococcus"
##  [7] "ASV13:g__Enterococcus"        "ASV99:g__Schaalia"
##  [9] "ASV2:g__Prevotella"           "ASV22:g__Leptotrichia"
## [11] "ASV2326:o__Actinomycetales"   "ASV3182:f__Micrococcaceae"
## [13] "ASV1876:g__Micrococcus"       "ASV660:f__Streptococcaceae"
## [15] "ASV77:g__Alloprevotella"
```

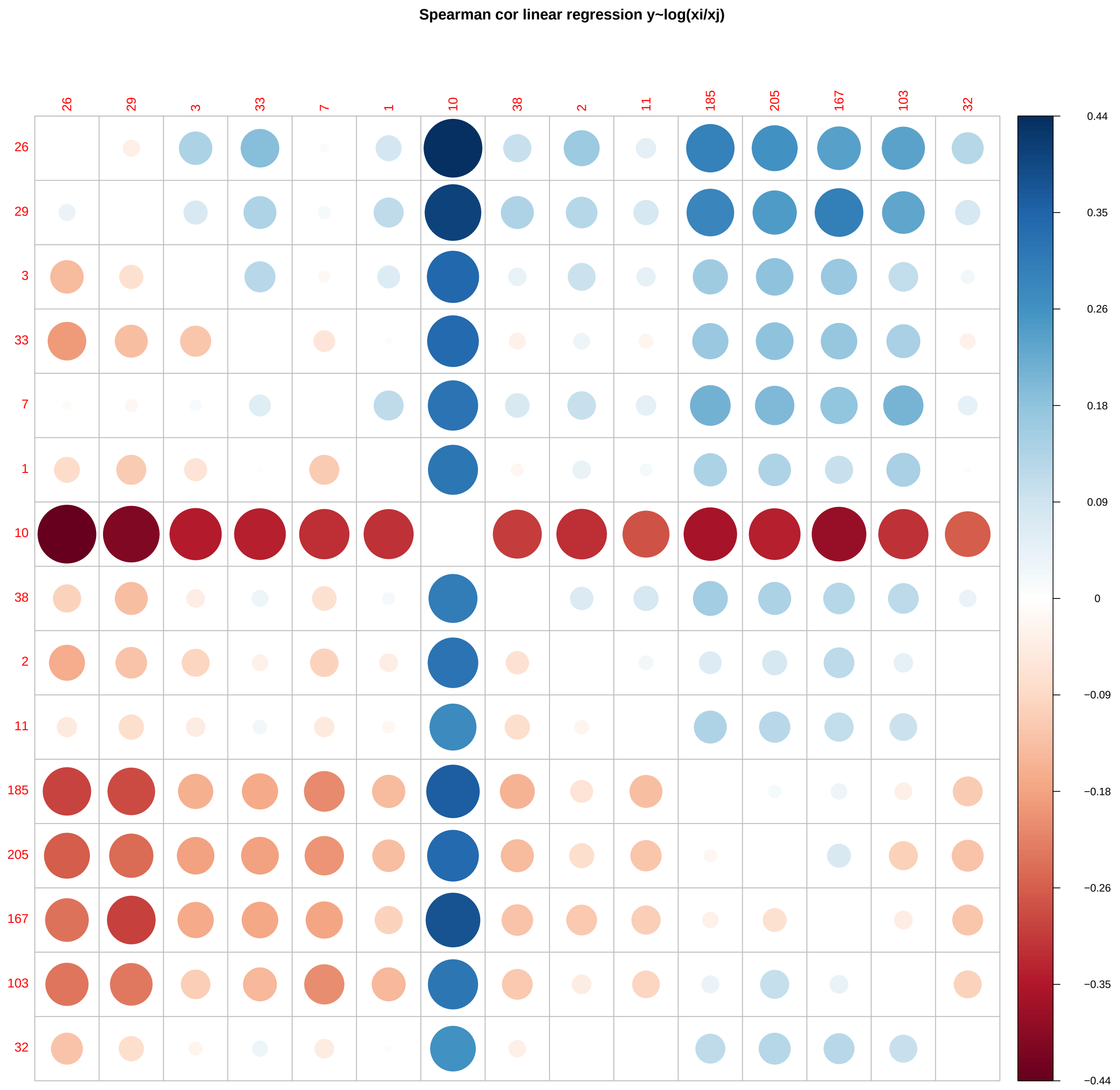

Figure 16: Correlation-like plot of the association of pairwise log-ratios with MCH.

The pair of taxa whose log-ratio is more associated with the variable

```r
var_logratios$`max log-ratio`
```

```
## [1] "10" "26"
```

```r
var_logratios$`names max log-ratio`
```

```
## [1] "ASV13:g__Enterococcus"        "ASV62:g__Lachnoanaerobaculum"
```

The correlation value between the log-ratios and the variable

```r
var_logratios$`association log-ratio with y`[1:15,1:15]
```

```
##                26          29           3           33            7            1
## 26   0.000000000 -0.03534176  0.13982783  0.187295283  0.009532602  0.083234757
## 29   0.035341756  0.00000000  0.07171285  0.135728265  0.020419609  0.112543348
## 3   -0.139827827 -0.07171285  0.00000000  0.120434648 -0.017095538  0.064963045
## 33  -0.187295283 -0.13572826 -0.12043465  0.000000000 -0.058152183 -0.004424356
## 7   -0.009532602 -0.02041961  0.01709554  0.058152183  0.000000000  0.111900555
## 1   -0.083234757 -0.11254335 -0.06496305  0.004424356 -0.111900555  0.000000000
## 10  -0.439538513 -0.40793274 -0.34609575 -0.339451451 -0.320942347 -0.317498337
## 38  -0.098641160 -0.13603541 -0.04122076  0.034471915 -0.074686988  0.019256414
## 2   -0.162229820 -0.12479743 -0.09652825 -0.033808137 -0.100836323 -0.041699437
## 11  -0.050203457 -0.07891516 -0.04567275  0.025657510 -0.050575440 -0.018941856
## 185 -0.297067243 -0.28724547 -0.15530955 -0.164156549 -0.208422250 -0.137989290
## 205 -0.266845412 -0.24726625 -0.17698669 -0.178847180 -0.194953170 -0.132934906
## 167 -0.240615341 -0.30003557 -0.16500043 -0.167621808 -0.174289168 -0.098587224
## 103 -0.235285672 -0.22927681 -0.10885829 -0.143979761 -0.203337112 -0.144376227
## 32  -0.127425917 -0.07839598 -0.02355081  0.030855082 -0.046924154 -0.005757777
##            10          38            2           11         185         205
## 26  0.4395385  0.09864116  0.162229820  0.050203457  0.29706724  0.26684541
## 29  0.4079327  0.13603541  0.124797429  0.078915162  0.28724547  0.24726625
## 3   0.3460958  0.04122076  0.096528247  0.045672752  0.15530955  0.17698669
## 33  0.3394515 -0.03447191  0.033808137 -0.025657510  0.16415655  0.17884718
## 7   0.3209423  0.07468699  0.100836323  0.050575440  0.20842225  0.19495317
## 1   0.3174983 -0.01925641  0.041699437  0.018941856  0.13798929  0.13293491
## 10  0.0000000 -0.30371457 -0.324610081 -0.279077105 -0.36330474 -0.33941427
## 38  0.3037146  0.00000000  0.067260686  0.078368090  0.15253786  0.13642706
## 2   0.3246101 -0.06726069  0.000000000  0.025725366  0.06392364  0.07738124
## 11  0.2790771 -0.07836809 -0.025725366  0.000000000  0.13569778  0.12203947
## 185 0.3633047 -0.15253786 -0.063923637 -0.135697782  0.00000000  0.02086949
## 205 0.3394143 -0.13642706 -0.077381244 -0.122039467 -0.02086949  0.00000000
## 167 0.3797451 -0.12425547 -0.117849803 -0.105960405 -0.03203998 -0.07046446
## 103 0.3194479 -0.11762132 -0.046404129 -0.094848640  0.03878252  0.10452503
## 32  0.2639368 -0.03691294  0.003049844 -0.002345732  0.11148194  0.12723393
##             167         103           32
## 26   0.24061534  0.23528567  0.127425917
## 29   0.30003557  0.22927681  0.078395980
## 3    0.16500043  0.10885829  0.023550814
## 33   0.16762181  0.14397976 -0.030855082
## 7    0.17428917  0.20333711  0.046924154
## 1    0.09858722  0.14437623  0.005757777
## 10  -0.37974506 -0.31944788 -0.263936779
## 38   0.12425547  0.11762132  0.036912939
## 2    0.11784980  0.04640413 -0.003049844
## 11   0.10596040  0.09484864  0.002345732
```

```
## 185  0.03203998 -0.03878252 -0.111481941
## 205  0.07046446 -0.10452503 -0.127233933
## 167  0.00000000 -0.04234024 -0.119138321
## 103  0.04234024  0.00000000 -0.097073111
## 32   0.11913832  0.09707311  0.000000000
```

```r
library(coda4microbiome)
library(microbiomeutilities)
set.seed(123)

ps <- readRDS("ps1.dna.genus_n0.rds")
ps <- format_to_besthit(ps)
abundance <- as.data.frame(otu_table(ps))
metadata <- sample_data(ps)

abundance <- abundance[-c(65,78),]
metadata <- metadata[-c(65,78),]

abundance <- as.matrix(abundance)

var_logratios<-explore_logratios(x=abundance, y=metadata$MCHC, measure = "glm")
```

5.1.1.15 Mean Corpuscular Hemoglobin Concentration (MCHC) Results

Name of the most important variables

```r
var_logratios$`name of most important variables`
```

```
##  [1] "ASV38:g__Novosphingobium"          "ASV465:g__Lawsonella"
##  [3] "ASV464:f__Enterobacteriaceae"      "ASV13:g__Enterococcus"
##  [5] "ASV110:g__Achromobacter"           "ASV238:g__Selenomonas"
##  [7] "ASV275:g__Catonella"               "ASV260:g__Sphingomonas"
##  [9] "ASV2613:f__Caulobacteraceae"       "ASV2409:g__Pyramidobacter"
## [11] "ASV208:g__Cutibacterium"           "ASV3:g__Veillonella"
## [13] "ASV524:g__Caulobacter"             "ASV2229:g__Peptoniphilaceae_[G-1]"
## [15] "ASV5324:f__Bifidobacteriaceae"
```

The pair of taxa whose log-ratio is more associated with the variable

```r
var_logratios$`max log-ratio`
```

```
## [1] "186" "195"
```

```r
var_logratios$`names max log-ratio`
```

```
## [1] "ASV2409:g__Pyramidobacter"   "ASV2613:f__Caulobacteraceae"
```

The correlation value between the log-ratios and the variable

```r
var_logratios$`association log-ratio with y`[1:15,1:15]
```

```
##              15          84          83         10          40           58
## 15   0.00000000  0.094687088  0.38472769 0.36114450  0.22606160  0.121028962
## 84  -0.09468709  0.000000000  0.36096633 0.33155773 -0.02007763 -0.026772267
## 83  -0.38472769 -0.360966326  0.00000000 0.05436946 -0.30507261 -0.250147138
## 10  -0.36114450 -0.331557732 -0.05436946 0.00000000 -0.30498145 -0.234332823
## 40  -0.22606160  0.020077631  0.30507261 0.30498145  0.00000000  0.027857270
```

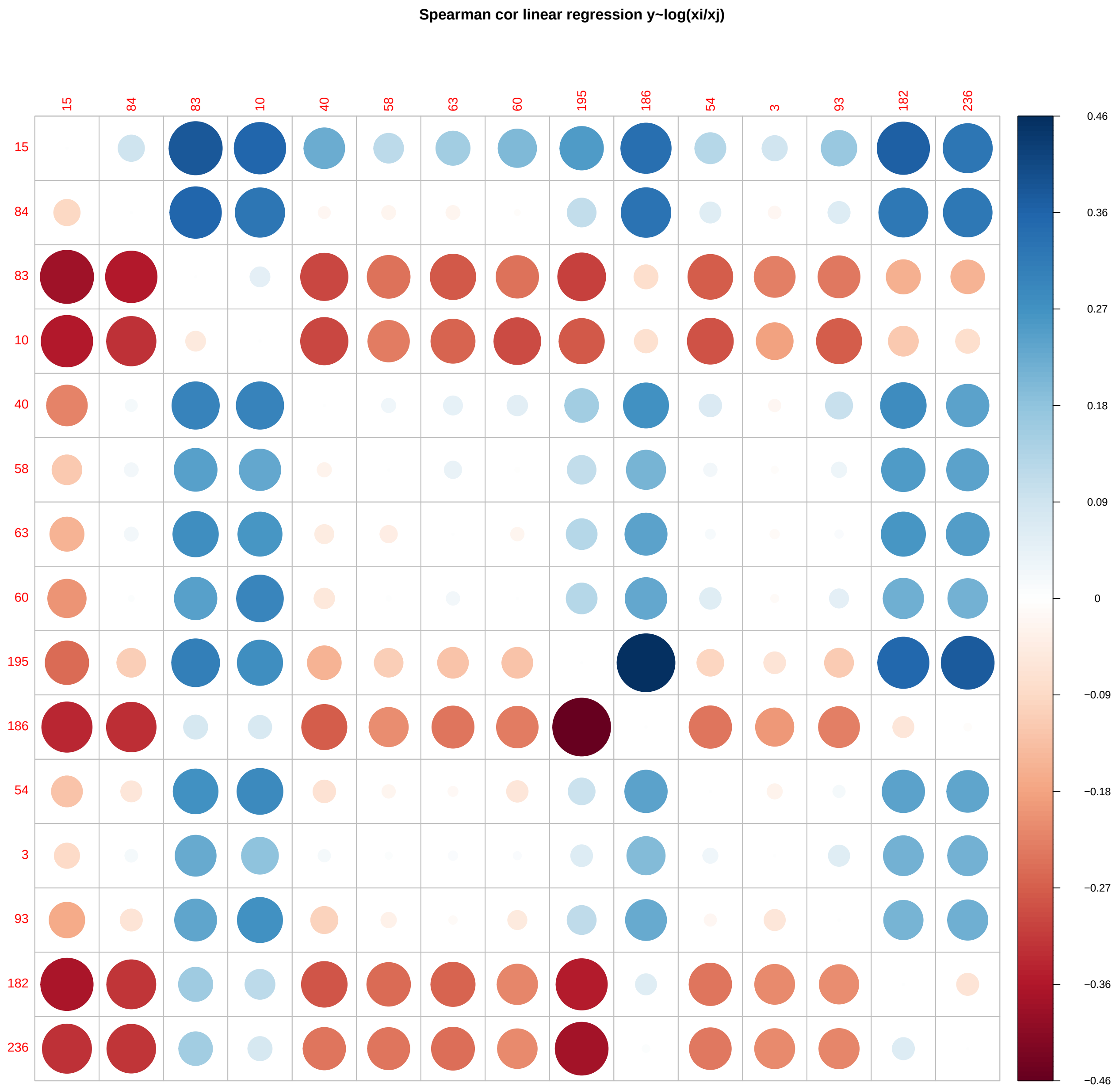

Figure 17: Correlation-like plot of the association of pairwise log-ratios with MCHC.

```
## 58  -0.12102896  0.026772267  0.25014714 0.23433282 -0.02785727  0.000000000
## 63  -0.15897903  0.026993015  0.28078326 0.26486616 -0.04981561 -0.041287293
## 60  -0.20065335  0.004834969  0.24612729 0.29785312 -0.05776730  0.003317964
## 195 -0.25754019 -0.113456434  0.31156118 0.27921815 -0.15612511 -0.112702791
## 186 -0.34402675 -0.334721629  0.07923162 0.07501785 -0.27738680 -0.209621772
## 54  -0.13128195 -0.060649741  0.27368988 0.28712519 -0.06986538 -0.024641532
## 3   -0.08717127  0.022490515  0.22848784 0.18540868  0.02095831  0.007086573
## 93  -0.17233444 -0.066843181  0.24005431 0.27759832 -0.10075739 -0.032690206
## 182 -0.37297460 -0.327256734  0.15960653 0.12240993 -0.28349991 -0.258713198
## 236 -0.32893293 -0.323643933  0.15509907 0.08001034 -0.24512050 -0.241832038
##               63           60          195          186          54            3
## 15   0.158979033  0.200653350  0.25754019  0.344026754  0.13128195  0.087171266
## 84  -0.026993015 -0.004834969  0.11345643  0.334721629  0.06064974 -0.022490515
## 83  -0.280783259 -0.246127292 -0.31156118 -0.079231622 -0.27368988 -0.228487841
## 10  -0.264866163 -0.297853116 -0.27921815 -0.075017846 -0.28712519 -0.185408676
## 40   0.049815611  0.057767302  0.15612511  0.277386804  0.06986538 -0.020958314
## 58   0.041287293 -0.003317964  0.11270279  0.209621772  0.02464153 -0.007086573
## 63   0.000000000 -0.024839367  0.12990842  0.241592218  0.01381580 -0.012230160
## 60   0.024839367  0.000000000  0.12985443  0.235982097  0.06328811 -0.009165780
## 195 -0.129908419 -0.129854426  0.00000000  0.455692984 -0.09814643 -0.065009079
## 186 -0.241592218 -0.235982097 -0.45569298  0.000000000 -0.24487165 -0.199389911
## 54  -0.013815804 -0.063288107  0.09814643  0.244871647  0.00000000 -0.031601420
## 3    0.012230160  0.009165780  0.06500908  0.199389911  0.03160142  0.000000000
## 93  -0.009753527 -0.050234063  0.11465619  0.228805262 -0.02064803 -0.061698010
## 182 -0.266038078 -0.222556772 -0.35734313  0.060908432 -0.24550431 -0.216956858
## 236 -0.251540494 -0.214481176 -0.38044918  0.008113174 -0.23780562 -0.218502739
##               93          182          236
## 15   0.172334439  0.37297460  0.328932933
## 84   0.066843181  0.32725673  0.323643933
## 83  -0.240054314 -0.15960653 -0.155099071
## 10  -0.277598319 -0.12240993 -0.080010342
## 40   0.100757388  0.28349991  0.245120502
## 58   0.032690206  0.25871320  0.241832038
## 63   0.009753527  0.26603808  0.251540494
## 60   0.050234063  0.22255677  0.214481176
## 195 -0.114656192  0.35734313  0.380449182
## 186 -0.228805262 -0.06090843 -0.008113174
## 54   0.020648031  0.24550431  0.237805617
## 3    0.061698010  0.21695686  0.218502739
## 93   0.000000000  0.21124701  0.220584800
## 182 -0.211247012  0.00000000 -0.066861458
## 236 -0.220584800  0.06686146  0.000000000
```

```r
library(coda4microbiome)
library(microbiomeutilities)
set.seed(123)

ps <- readRDS("ps1.dna.genus_n0.rds")
ps <- format_to_besthit(ps)
abundance <- as.data.frame(otu_table(ps))
metadata <- sample_data(ps)

abundance <- abundance[-c(65,78),]
```

```r
metadata <- metadata[-c(65,78),]

abundance <- as.matrix(abundance)

var_logratios<-explore_logratios(x=abundance, y=metadata$MCV, measure = "glm")
```

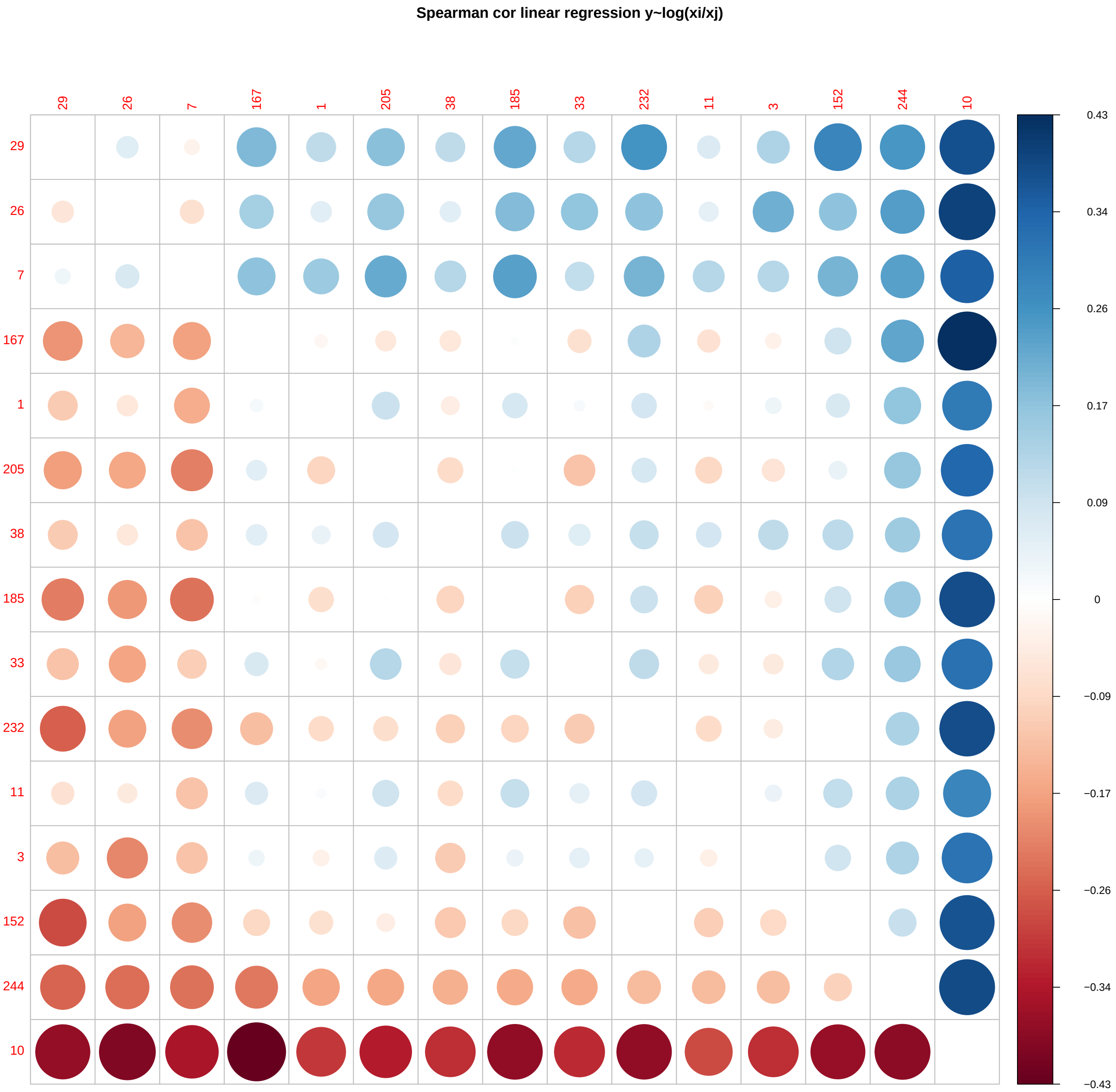

Figure 18: Correlation-like plot of the association of pairwise log-ratios with MCV.

5.1.1.16 Mean Corpuscular Volume (MCV) Results

Name of the most important variables

```r
var_logratios$`name of most important variables`
```

```
##  [1] "ASV69:g__Actinomyces"         "ASV62:g__Lachnoanaerobaculum"
```

```
##  [3] "ASV8:g__Rothia"               "ASV1876:g__Micrococcus"
##  [5] "ASV1:g__Streptococcus"        "ASV3182:f__Micrococcaceae"
##  [7] "ASV99:g__Schaalia"            "ASV2326:o__Actinomycetales"
##  [9] "ASV82:g__Atopobium"           "ASV4831:f__Actinomycetaceae"
## [11] "ASV22:g__Leptotrichia"        "ASV3:g__Veillonella"
## [13] "ASV1409:o__Micrococcales"     "ASV6995:f__Listeriaceae"
## [15] "ASV13:g__Enterococcus"
```

The pair of taxa whose log-ratio is more associated with the variable

```
var_logratios$`max log-ratio`
```

```
## [1] "10"  "167"
```

```
var_logratios$`names max log-ratio`
```

```
## [1] "ASV13:g__Enterococcus"  "ASV1876:g__Micrococcus"
```

The correlation value between the log-ratios and the variable

```
var_logratios$`association log-ratio with y`[1:15,1:15]
```

```
##              29          26           7          167           1         205
## 29   0.00000000  0.05943329 -0.02971718  0.192009738  0.10803642  0.17663426
## 26  -0.05943329  0.00000000 -0.06984455  0.142646146  0.05457876  0.16505651
## 7    0.02971718  0.06984455  0.00000000  0.175087674  0.15602527  0.21507141
## 167 -0.19200974 -0.14264615 -0.17508767  0.000000000 -0.02086367 -0.05192194
## 1   -0.10803642 -0.05457876 -0.15602527  0.020863668  0.00000000  0.09392688
## 205 -0.17663426 -0.16505651 -0.21507141  0.051921936 -0.09392688  0.00000000
## 38  -0.10760112 -0.05419965 -0.12127095  0.054929655  0.04204151  0.08085918
## 185 -0.21941113 -0.18566379 -0.23336911 -0.007235828 -0.07716549 -0.00367925
## 33  -0.12378908 -0.16798381 -0.10359301  0.069068535 -0.01568193  0.12051026
## 232 -0.25639642 -0.17352288 -0.20086136 -0.130955687 -0.07752097 -0.07514461
## 11  -0.06447908 -0.04740483 -0.12277488  0.064329764  0.01203139  0.08693304
## 3   -0.13171729 -0.20650242 -0.12020453  0.031158781 -0.03274452  0.06387575
## 152 -0.27883659 -0.17469136 -0.19928492 -0.086787085 -0.06967317 -0.04114528
## 244 -0.25042233 -0.23782297 -0.23392808 -0.225074541 -0.16893282 -0.16361079
## 10  -0.37303882 -0.39700815 -0.35151283 -0.428748772 -0.30342348 -0.33860694
##              38          185          33          232          11           3
## 29   0.10760112  0.219411132  0.12378908  0.2563964194  0.06447908  0.13171729
## 26   0.05419965  0.185663791  0.16798381  0.1735228754  0.04740483  0.20650242
## 7    0.12127095  0.233369110  0.10359301  0.2008613624  0.12277488  0.12020453
## 167 -0.05492965  0.007235828 -0.06906854  0.1309556871 -0.06432976 -0.03115878
## 1   -0.04204151  0.077165495  0.01568193  0.0775209700 -0.01203139  0.03274452
## 205 -0.08085918  0.003679250 -0.12051026  0.0751446098 -0.08693304 -0.06387575
## 38   0.00000000  0.091965024  0.05854446  0.1015943337  0.07815149  0.11071614
## 185 -0.09196502  0.000000000 -0.10231445  0.0924062656 -0.09943324 -0.03485002
## 33  -0.05854446  0.102314451  0.00000000  0.1082319159 -0.04854356 -0.05003965
## 232 -0.10159433 -0.092406266 -0.10823192  0.0000000000 -0.08126530 -0.04388723
## 11  -0.07815149  0.099433236  0.04854356  0.0812652967  0.00000000  0.03534929
## 3   -0.11071614  0.034850018  0.05003965  0.0438872333 -0.03534929  0.00000000
## 152 -0.11531625 -0.085917973 -0.12696904 -0.0002702423 -0.10339735 -0.08348202
## 244 -0.15042457 -0.160163924 -0.15978504 -0.1370559482 -0.13609917 -0.13240089
## 10  -0.31473240 -0.380774952 -0.31849877 -0.3780952984 -0.28198853 -0.31620683
##               152         244        10
## 29   0.2788365946  0.25042233 0.3730388
## 26   0.1746913587  0.23782297 0.3970081
```

```
## 7    0.1992849237  0.23392808 0.3515128
## 167  0.0867870854  0.22507454 0.4287488
## 1    0.0696731681  0.16893282 0.3034235
## 205  0.0411452820  0.16361079 0.3386069
## 38   0.1153162516  0.15042457 0.3147324
## 185  0.0859179733  0.16016392 0.3807750
## 33   0.1269690423  0.15978504 0.3184988
## 232  0.0002702423  0.13705595 0.3780953
## 11   0.1033973514  0.13609917 0.2819885
## 3    0.0834820182  0.13240089 0.3162068
## 152  0.0000000000  0.09493364 0.3711890
## 244 -0.0949336414  0.00000000 0.3842703
## 10  -0.3711889976 -0.38427030 0.0000000
```

```
library(coda4microbiome)
library(microbiomeutilities)
set.seed(123)

ps <- readRDS("ps1.dna.genus_n0.rds")
ps <- format_to_besthit(ps)
abundance <- as.data.frame(otu_table(ps))
metadata <- sample_data(ps)

abundance <- abundance[-c(65,78),]
metadata <- metadata[-c(65,78),]

abundance <- as.matrix(abundance)

var_logratios<-
  explore_logratios(x=abundance, y=metadata$Monocytes...., measure = "glm")
```

5.1.1.17 Monocytes (%) Results

Name of the most important variables

```
var_logratios$`name of most important variables`
```

```
##  [1] "ASV212:g__Saccharibacteria_(TM7)_[G-3]"
##  [2] "ASV733:g__Filifactor"
##  [3] "ASV27:g__Haemophilus"
##  [4] "ASV13:g__Enterococcus"
##  [5] "ASV823:g__Peptococcus"
##  [6] "ASV1145:g__Jeotgalicoccus"
##  [7] "ASV1198:f__Staphylococcaceae"
##  [8] "ASV24:g__Fusobacterium"
##  [9] "ASV7:g__Agrobacterium"
## [10] "ASV929:g__Bacteroidetes_[G-3]"
## [11] "ASV272:g__Peptidiphaga"
## [12] "ASV130:g__Absconditabacteria_(SR1)_[G-1]"
## [13] "ASV44:g__Gemella"
## [14] "ASV110:g__Achromobacter"
## [15] "ASV136:g__Mesorhizobium"
```

The pair of taxa whose log-ratio is more associated with the variable

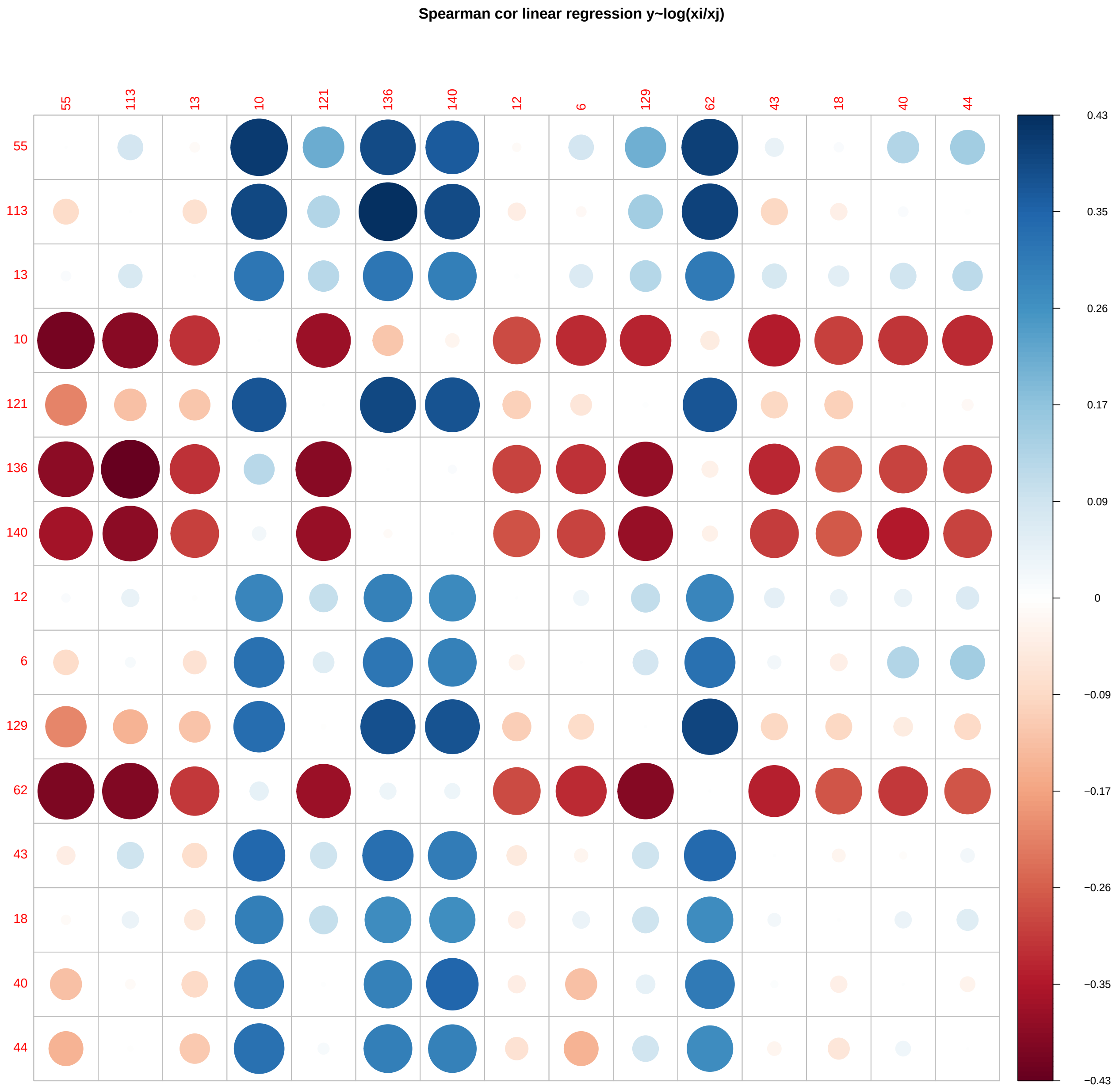

Figure 19: Correlation-like plot of the association of pairwise log-ratios with monocytes (%).

```r
var_logratios$`max log-ratio`
```

```
## [1] "136" "113"
```

```r
var_logratios$`names max log-ratio`
```

```
## [1] "ASV1145:g__Jeotgalicoccus" "ASV733:g__Filifactor"
```

The correlation value between the log-ratios and the variable

```r
var_logratios$`association log-ratio with y`[1:15,1:15]
```

```
##                55          113          13         10          121          136
## 55   0.000000000  0.080740295 -0.012344159 0.41286620  0.214489495  0.387241193
## 113 -0.080740295  0.000000000 -0.072002134 0.39288943  0.129556294  0.432233833
## 13   0.012344159  0.072002134  0.000000000 0.31486458  0.120693547  0.312869297
## 10  -0.412866197 -0.392889433 -0.314864585 0.00000000 -0.371380679 -0.117182638
## 121 -0.214489495 -0.129556294 -0.120693547 0.37138068  0.000000000  0.392748372
## 136 -0.387241193 -0.432233833 -0.312869297 0.11718264 -0.392748372  0.000000000
## 140 -0.360420539 -0.387723367 -0.294165517 0.02429867 -0.375320341 -0.008892381
## 12   0.009459191  0.039113902 -0.002571397 0.28395391  0.099765411  0.293763209
## 6   -0.078869941  0.013155015 -0.068493714 0.32013259  0.056684287  0.313706861
## 129 -0.211283891 -0.148833722 -0.123445985 0.33146933 -0.002922372  0.379045937
## 62  -0.405478535 -0.397931329 -0.303670831 0.04406343 -0.370098699  0.033898464
## 43  -0.043072977  0.088898759 -0.076743152 0.34011582  0.089488785  0.327127532
## 18  -0.011405700  0.035663460 -0.054189517 0.29514241  0.101234912  0.271717497
## 40  -0.126022342 -0.012503570 -0.085638476 0.30781520  0.002260514  0.292242340
## 44  -0.149642262 -0.003324673 -0.112825811 0.31991747  0.016352958  0.296126513
##               140          12           6          129          62           43
## 55   0.360420539 -0.009459191  0.07886994  0.211283891  0.40547854  0.043072977
## 113  0.387723367 -0.039113902 -0.01315502  0.148833722  0.39793133 -0.088898759
## 13   0.294165517  0.002571397  0.06849371  0.123445985  0.30367083  0.076743152
## 10  -0.024298668 -0.283953912 -0.32013259 -0.331469333 -0.04406343 -0.340115819
## 121  0.375320341 -0.099765411 -0.05668429  0.002922372  0.37009870 -0.089488785
## 136  0.008892381 -0.293763209 -0.31370686 -0.379045937 -0.03389846 -0.327127532
## 140  0.000000000 -0.275341907 -0.29365960 -0.375790398 -0.03046028 -0.298557151
## 12   0.275341907  0.000000000  0.02993566  0.103861728  0.28345278  0.050783815
## 6    0.293659598 -0.029935657  0.00000000  0.081515605  0.32414606  0.023119127
## 129  0.375790398 -0.103861728 -0.08151561  0.000000000  0.39724534 -0.089332674
## 62   0.030460280 -0.283452777 -0.32414606 -0.397245338  0.00000000 -0.336793029
## 43   0.298557151 -0.050783815 -0.02311913  0.089332674  0.33679303  0.000000000
## 18   0.264858139 -0.036321120  0.03732124  0.087403961  0.27096329  0.021964941
## 40   0.343100467 -0.038971252 -0.12619226  0.045921543  0.30678166  0.006592621
## 44   0.293530547 -0.065805130 -0.14965602  0.085680710  0.26863471 -0.024360486
##              18           40           44
## 55   0.01140570  0.126022342  0.149642262
## 113 -0.03566346  0.012503570  0.003324673
## 13   0.05418952  0.085638476  0.112825811
## 10  -0.29514241 -0.307815204 -0.319917470
## 121 -0.10123491 -0.002260514 -0.016352958
## 136 -0.27171750 -0.292242340 -0.296126513
## 140 -0.26485814 -0.343100467 -0.293530547
## 12   0.03632112  0.038971252  0.065805130
## 6   -0.03732124  0.126192259  0.149656023
## 129 -0.08740396 -0.045921543 -0.085680710
## 62  -0.27096329 -0.306781660 -0.268634713
```

```
## 43  -0.02196494 -0.006592621  0.024360486
## 18   0.00000000  0.034968432  0.058198779
## 40  -0.03496843  0.000000000 -0.028661619
## 44  -0.05819878  0.028661619  0.000000000
```

```r
library(coda4microbiome)
library(microbiomeutilities)
set.seed(123)

ps <- readRDS("ps1.dna.genus_n0.rds")
ps <- format_to_besthit(ps)
abundance <- as.data.frame(otu_table(ps))
metadata <- sample_data(ps)

abundance <- abundance[-c(65,78),]
metadata <- metadata[-c(65,78),]

abundance <- as.matrix(abundance)

var_logratios<-explore_logratios(x=abundance, y=metadata$Monocytes, measure = "glm")
```

5.1.1.18 Monocytes Results

Name of the most important variables

```r
var_logratios$`name of most important variables`
```

```
##  [1] "ASV929:g__Bacteroidetes_[G-3]"
##  [2] "ASV150:g__Parvimonas"
##  [3] "ASV212:g__Saccharibacteria_(TM7)_[G-3]"
##  [4] "ASV76:g__Bosea"
##  [5] "ASV349:g__Dialister"
##  [6] "ASV1145:g__Jeotgalicoccus"
##  [7] "ASV733:g__Filifactor"
##  [8] "ASV248:g__Treponema"
##  [9] "ASV667:c__Firmicutes"
## [10] "ASV110:g__Achromobacter"
## [11] "ASV1213:g__Olsenella"
## [12] "ASV13:g__Enterococcus"
## [13] "ASV4909:o__Bacteroidales"
## [14] "ASV7:g__Agrobacterium"
## [15] "ASV2210:g__Arcanobacterium"
```

The pair of taxa whose log-ratio is more associated with the variable

```r
var_logratios$`max log-ratio`
```

```
## [1] "136" "129"
```

```r
var_logratios$`names max log-ratio`
```

```
## [1] "ASV1145:g__Jeotgalicoccus"     "ASV929:g__Bacteroidetes_[G-3]"
```

The correlation value between the log-ratios and the variable

```r
var_logratios$`association log-ratio with y`[1:15,1:15]
```

```
##               129          45          55          31          72
```

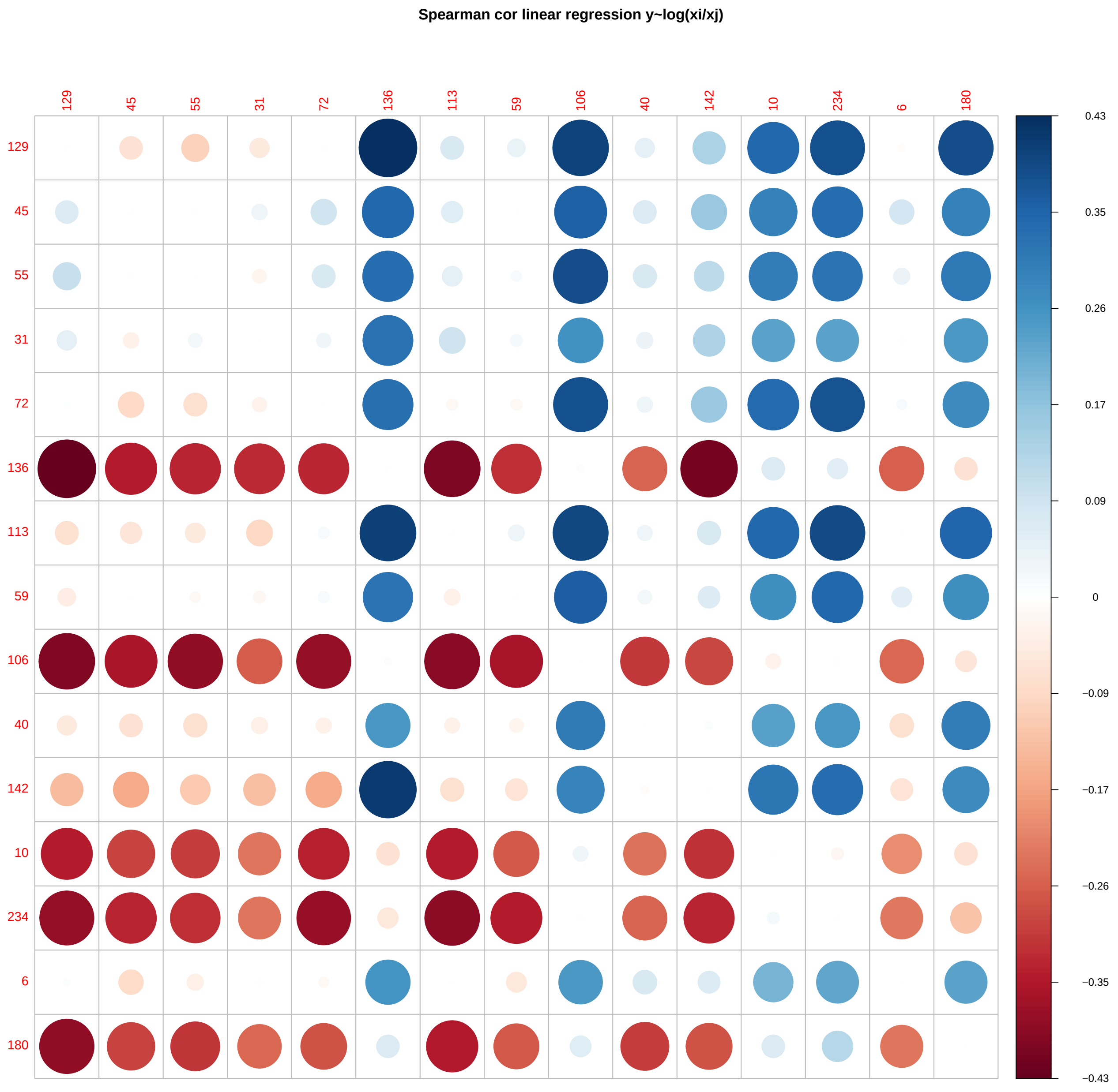

Figure 20: Correlation-like plot of the association of pairwise log-ratios with monocytes.

```
## 129  0.000000000 -0.0670860944 -0.096685565 -0.049653886 -0.002854544
## 45   0.067086094  0.0000000000  0.001930125  0.031385230  0.084906372
## 55   0.096685565 -0.0019301249  0.000000000 -0.025245998  0.069266959
## 31   0.049653886 -0.0313852298  0.025245998  0.000000000  0.027687196
## 72   0.002854544 -0.0849063718 -0.069266959 -0.027687196  0.000000000
## 136 -0.431769031 -0.3406614080 -0.328656279 -0.323052625 -0.325296801
## 113 -0.069188181 -0.0594727736 -0.051560086 -0.087224137  0.017138606
## 59  -0.041594645 -0.0003647554 -0.014620842 -0.019334108  0.017157061
## 106 -0.400538793 -0.3503127680 -0.383919598 -0.261368610 -0.379752374
## 40  -0.048799866 -0.0682619777 -0.071007983 -0.035330013 -0.031070454
## 142 -0.135937294 -0.1606092798 -0.114981512 -0.130509390 -0.162946852
## 10  -0.338509010 -0.2924352432 -0.301909151 -0.232072905 -0.334756534
## 234 -0.378860638 -0.3308023227 -0.319327090 -0.230291180 -0.373514277
## 6    0.006316224 -0.0782194343 -0.034798344  0.003411826 -0.013891482
## 180 -0.383014096 -0.2925885153 -0.310552788 -0.247109501 -0.272085814
##              136           113           59          106           40
## 129  0.431769031  0.0691881809  0.0415946446 0.400538793  0.048799866
## 45   0.340661408  0.0594727736  0.0003647554 0.350312768  0.068261978
## 55   0.328656279  0.0515600864  0.0146208425 0.383919598  0.071007983
## 31   0.323052625  0.0872241371  0.0193341082 0.261368610  0.035330013
## 72   0.325296801 -0.0171386055 -0.0171570614 0.379752374  0.031070454
## 136  0.000000000 -0.4034727220 -0.3167104158 0.006536842 -0.253032268
## 113  0.403472722  0.0000000000  0.0339068597 0.392106722  0.030258243
## 59   0.316710416 -0.0339068597  0.0000000000 0.355079102  0.025686977
## 106 -0.006536842 -0.3921067222 -0.3550791025 0.000000000 -0.303686687
## 40   0.253032268 -0.0302582427 -0.0256869768 0.303686687  0.000000000
## 142  0.413906570 -0.0710601840 -0.0626221938 0.287983100 -0.008203604
## 10  -0.067085902 -0.3392700066 -0.2665281282 0.029656672 -0.233868922
## 234 -0.054346372 -0.3874178669 -0.3369795738 0.003747393 -0.253029343
## 6    0.255181793  0.0002612912 -0.0524206752 0.246535731  0.073337889
## 180  0.066564741 -0.3413316162 -0.2636765321 0.057417741 -0.299267305
##              142         10          234             6         180
## 129  0.135937294  0.33850901  0.378860638 -0.0063162241  0.38301410
## 45   0.160609280  0.29243524  0.330802323  0.0782194343  0.29258852
## 55   0.114981512  0.30190915  0.319327090  0.0347983439  0.31055279
## 31   0.130509390  0.23207291  0.230291180 -0.0034118258  0.24710950
## 72   0.162946852  0.33475653  0.373514277  0.0138914822  0.27208581
## 136 -0.413906570  0.06708590  0.054346372 -0.2551817932 -0.06656474
## 113  0.071060184  0.33927001  0.387417867 -0.0002612912  0.34133162
## 59   0.062622194  0.26652813  0.336979574  0.0524206752  0.26367653
## 106 -0.287983100 -0.02965667 -0.003747393 -0.2465357310 -0.05741774
## 40   0.008203604  0.23386892  0.253029343 -0.0733378886  0.29926730
## 142  0.000000000  0.31491830  0.328334547 -0.0633789026  0.27484320
## 10  -0.314918303  0.00000000 -0.019218679 -0.2019749141 -0.06714378
## 234 -0.328334547  0.01921868  0.000000000 -0.2284335647 -0.12167150
## 6    0.063378903  0.20197491  0.228433565  0.0000000000  0.23144494
## 180 -0.274843200  0.06714378  0.121671504 -0.2314449425  0.00000000
```

```r
library(coda4microbiome)
library(microbiomeutilities)
set.seed(123)

ps <- readRDS("ps1.dna.genus_n0.rds")
```

```
ps <- format_to_besthit(ps)
abundance <- as.data.frame(otu_table(ps))
metadata <- sample_data(ps)

abundance <- abundance[-c(65,78),]
metadata <- metadata[-c(65,78),]

abundance <- as.matrix(abundance)

var_logratios<-explore_logratios(x=abundance, y=metadata$RDW, measure = "glm")
```

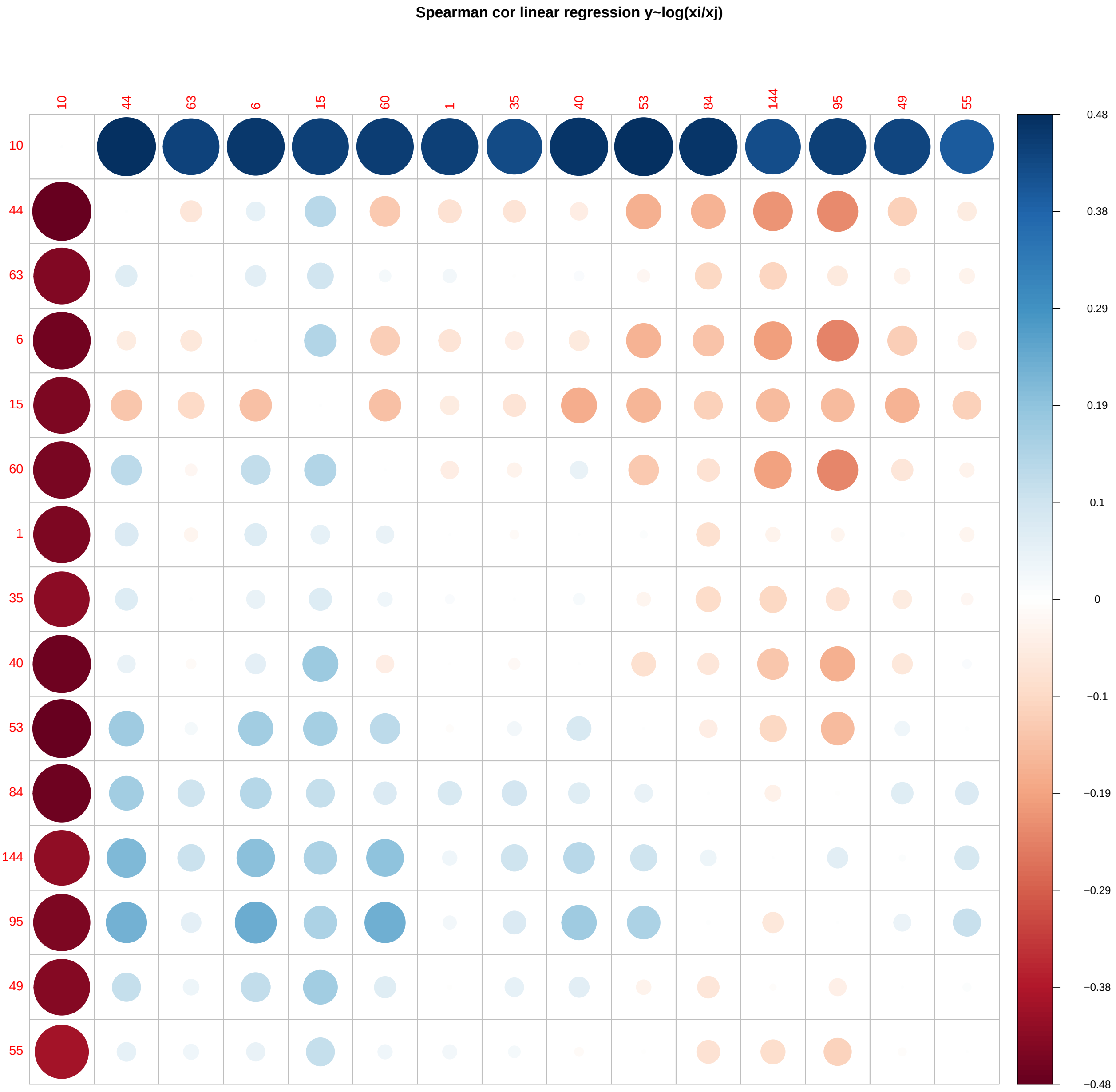

Figure 21: Correlation-like plot of the association of pairwise log-ratios with RDW.

5.1.1.19 Red Cell Distribution Width (RDW)   Results

Name of the most important variables

```
var_logratios$`name of most important variables`
```

```
##  [1] "ASV13:g__Enterococcus"
##  [2] "ASV136:g__Mesorhizobium"
##  [3] "ASV275:g__Catonella"
##  [4] "ASV7:g__Agrobacterium"
##  [5] "ASV38:g__Novosphingobium"
##  [6] "ASV260:g__Sphingomonas"
##  [7] "ASV1:g__Streptococcus"
##  [8] "ASV92:g__Saccharibacteria_(TM7)_[G-1]"
##  [9] "ASV110:g__Achromobacter"
## [10] "ASV203:g__Mycobacterium"
## [11] "ASV465:g__Lawsonella"
## [12] "ASV1291:o__Rhizobiales"
## [13] "ASV543:g__Afipia"
## [14] "ASV169:c__Alphaproteobacteria"
## [15] "ASV212:g__Saccharibacteria_(TM7)_[G-3]"
```

The pair of taxa whose log-ratio is more associated with the variable

```
var_logratios$`max log-ratio`
```

```
## [1] "44" "10"
```

```
var_logratios$`names max log-ratio`
```

```
## [1] "ASV136:g__Mesorhizobium" "ASV13:g__Enterococcus"
```

The correlation value between the log-ratios and the variable

```
var_logratios$`association log-ratio with y`[1:15,1:15]
```

```
##             10          44          63           6          15          60
## 10   0.0000000  0.47749787  0.44065491  0.45895066 0.44443475  0.45064854
## 44  -0.4774979  0.00000000 -0.06373341  0.05000347 0.13180046 -0.12675216
## 63  -0.4406549  0.06373341  0.00000000  0.06037101 0.09492513  0.02001067
## 6   -0.4589507 -0.05000347 -0.06037101  0.00000000 0.14116453 -0.11751045
## 15  -0.4444348 -0.13180046 -0.09492513 -0.14116453 0.00000000 -0.13984563
## 60  -0.4506485  0.12675216 -0.02001067  0.11751045 0.13984563  0.00000000
## 1   -0.4467514  0.07510139 -0.02594579  0.06834486 0.05029133  0.04341137
## 35  -0.4250463  0.06843446  0.00101475  0.04665485 0.07074244  0.02902091
## 40  -0.4665243  0.04386765 -0.01369209  0.05604855 0.17337845 -0.04381548
## 53  -0.4749387  0.17040247  0.02116454  0.16558147 0.16047005  0.12523566
## 84  -0.4672381  0.16262679  0.09760189  0.13434276 0.11195825  0.07226132
## 144 -0.4229020  0.21203453  0.10047769  0.19990237 0.15228195  0.19104762
## 95  -0.4486015  0.22888885  0.05554757  0.23810527 0.15164896  0.22928038
## 49  -0.4387495  0.11290523  0.03522390  0.11890988 0.16249465  0.06450567
## 55  -0.4009823  0.04962797  0.03256563  0.04762884 0.11162977  0.02911048
##                 1          35            40           53           84
## 10   0.4467513811  0.42504625  0.4665243046  0.474938719  0.467238147
## 44  -0.0751013909 -0.06843446 -0.0438676539 -0.170402468 -0.162626792
## 63   0.0259457863 -0.00101475  0.0136920859 -0.021164541 -0.097601890
## 6   -0.0683448644 -0.04665485 -0.0560485523 -0.165581475 -0.134342758
## 15  -0.0502913316 -0.07074244 -0.1733784508 -0.160470053 -0.111958255
## 60  -0.0434113667 -0.02902091  0.0438154767 -0.125235662 -0.072261318
## 1    0.0000000000 -0.01132470  0.0005744415  0.007577157 -0.077331300
```

```
## 35   0.0113247044  0.00000000  0.0182410904 -0.027028916 -0.086531601
## 40  -0.0005744415 -0.01824109  0.0000000000 -0.080860371 -0.062846589
## 53  -0.0075771573  0.02702892  0.0808603710  0.000000000 -0.044325234
## 84   0.0773312997  0.08653160  0.0628465891  0.044325234  0.000000000
## 144  0.0296794788  0.09914284  0.1335872828  0.097879779  0.036034127
## 95   0.0251796869  0.07467634  0.1685430325  0.151192002  0.002154691
## 49  -0.0026533909  0.04946578  0.0575653432 -0.030282739 -0.066043518
## 55   0.0285579501  0.01975498 -0.0110988028 -0.001831711 -0.074793238
##              144          95          49          55
## 10   0.422901959  0.448601465  0.438749460  0.400982333
## 44  -0.212034532 -0.228888849 -0.112905231 -0.049627974
## 63  -0.100477686 -0.055547573 -0.035223900 -0.032565631
## 6   -0.199902370 -0.238105267 -0.118909884 -0.047628843
## 15  -0.152281953 -0.151648959 -0.162494653 -0.111629772
## 60  -0.191047624 -0.229280378 -0.064505673 -0.029110482
## 1   -0.029679479 -0.025179687  0.002653391 -0.028557950
## 35  -0.099142836 -0.074676343 -0.049465784 -0.019754982
## 40  -0.133587283 -0.168543033 -0.057565343  0.011098803
## 53  -0.097879779 -0.151192002  0.030282739  0.001831711
## 84  -0.036034127 -0.002154691  0.066043518  0.074793238
## 144  0.000000000  0.058990301  0.005579413  0.082667561
## 95  -0.058990301  0.000000000  0.041837396  0.105411959
## 49  -0.005579413 -0.041837396  0.000000000  0.009241126
## 55  -0.082667561 -0.105411959 -0.009241126  0.000000000
```

```
library(coda4microbiome)
library(microbiomeutilities)
set.seed(123)

ps <- readRDS("ps1.dna.genus_n0.rds")
ps <- format_to_besthit(ps)
abundance <- as.data.frame(otu_table(ps))
metadata <- sample_data(ps)

abundance <- abundance[-c(65,78),]
metadata <- metadata[-c(65,78),]

metadata$Rod.neutrophils <- ifelse(is.na(metadata$Rod.neutrophils), 0, metadata$Rod.neutrophils)

abundance <- as.matrix(abundance)

var_logratios<-
  explore_logratios(x=abundance, y=metadata$Rod.neutrophils, measure = "glm")
```

5.1.1.20 Rod neutrophils   Results

Name of the most important variables

```
var_logratios$`name of most important variables`
```

```
##  [1] "ASV27:g__Haemophilus"
##  [2] "ASV52:g__Lactobacillus"
##  [3] "ASV947:g__Gardnerella"
##  [4] "ASV136:g__Mesorhizobium"
```

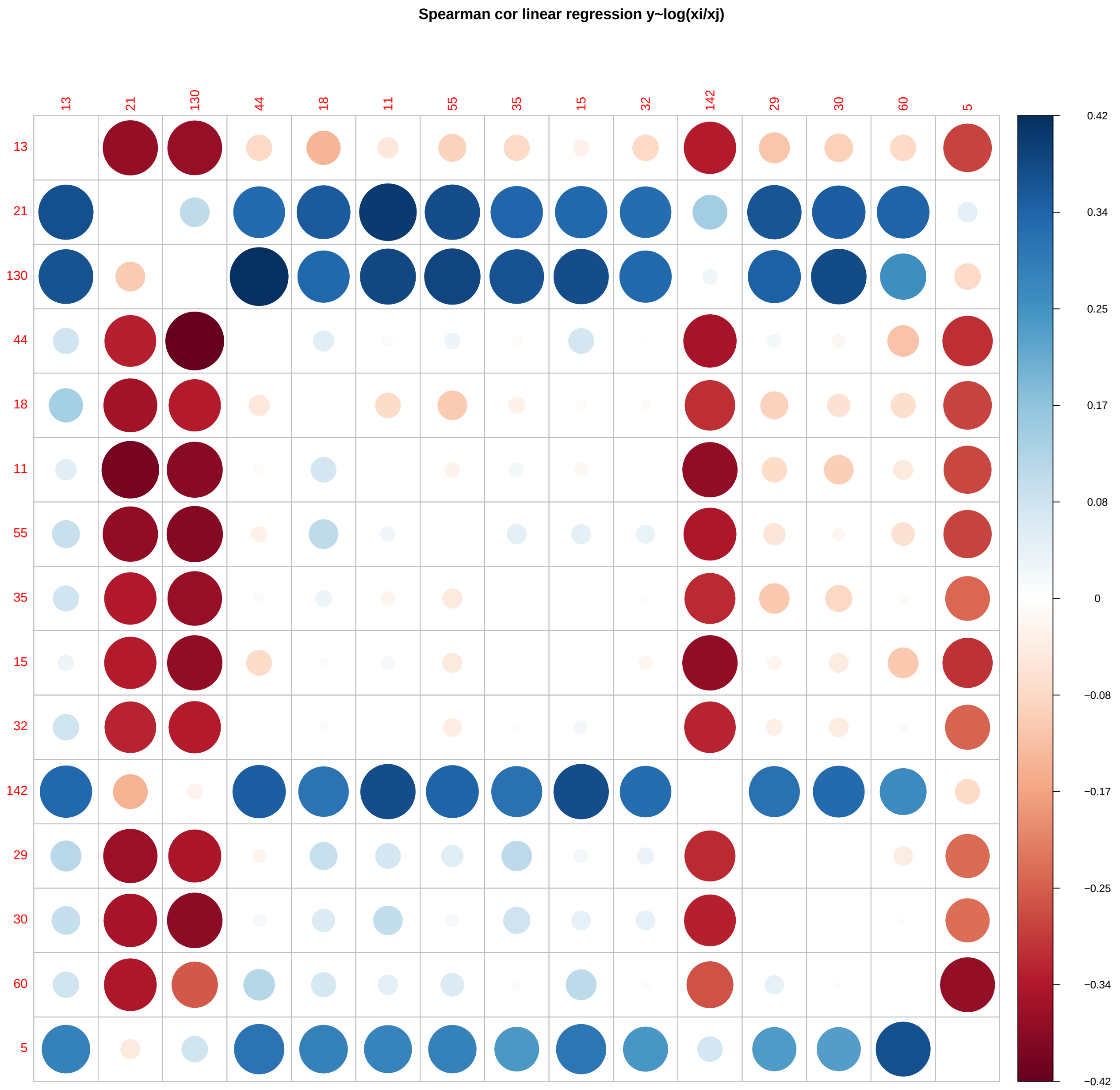

Figure 22: Correlation-like plot of the association of pairwise log-ratios with rod neutrophils.

```
##  [5] "ASV44:g__Gemella"
##  [6] "ASV22:g__Leptotrichia"
##  [7] "ASV212:g__Saccharibacteria_(TM7)_[G-3]"
##  [8] "ASV92:g__Saccharibacteria_(TM7)_[G-1]"
##  [9] "ASV38:g__Novosphingobium"
## [10] "ASV77:g__Alloprevotella"
## [11] "ASV1213:g__Olsenella"
## [12] "ASV69:g__Actinomyces"
## [13] "ASV73:g__Oribacterium"
## [14] "ASV260:g__Sphingomonas"
## [15] "ASV6:g__Staphylococcus"
```

The pair of taxa whose log-ratio is more associated with the variable

```
var_logratios$`max log-ratio`
```

```
## [1] NA NA
```

```
var_logratios$`names max log-ratio`
```

```
## [1] NA NA
```

The correlation value between the log-ratios and the variable

```
var_logratios$`association log-ratio with y`[1:15,1:15]
```

```
##             13          21         130           44          18           11
## 13  0.00000000 -0.36943913 -0.36344349 -0.081140718 -0.13937528 -0.052990239
## 21  0.36943913  0.00000000  0.10520117  0.324680309  0.35176786  0.403065879
## 130 0.36344349 -0.10520117  0.00000000  0.420446993  0.33102822  0.381102851
## 44  0.08114072 -0.32468031 -0.42044699  0.000000000  0.05219548  0.012404769
## 18  0.13937528 -0.35176786 -0.33102822 -0.052195484  0.00000000 -0.077784619
## 11  0.05299024 -0.40306588 -0.38110285 -0.012404769  0.07778462  0.000000000
## 55  0.09335513 -0.37213115 -0.38554504 -0.031273753  0.10518388  0.026729906
## 35  0.08080699 -0.33232992 -0.36166360  0.011353367  0.03290512 -0.024764064
## 15  0.03084413 -0.33169092 -0.37137896 -0.078797035  0.01113219  0.020867732
## 32  0.08347844 -0.32268783 -0.33102864 -0.002750589  0.01022810  0.001379272
## 142 0.33183309 -0.14516290 -0.02827250  0.344807451  0.30885976  0.370569594
## 29  0.11428269 -0.35803125 -0.34109883 -0.022589219  0.09366397  0.077388930
## 30  0.09778150 -0.34667967 -0.37514634  0.020424371  0.06344392  0.103985696
## 60  0.08244906 -0.33687059 -0.25880530  0.118828756  0.07485418  0.047837003
## 5   0.28506364 -0.04631932  0.08286085  0.307265802  0.28529608  0.278989227
##              55           35           15           32         142          29
## 13  -0.09335513 -0.080806987 -0.030844125 -0.083478437 -0.33183309 -0.11428269
## 21   0.37213115  0.332329924  0.331690924  0.322687828  0.14516290  0.35803125
## 130  0.38554504  0.361663599  0.371378961  0.331028640  0.02827250  0.34109883
## 44   0.03127375 -0.011353367  0.078797035  0.002750589 -0.34480745  0.02258922
## 18  -0.10518388 -0.032905117 -0.011132187 -0.010228101 -0.30885976 -0.09366397
## 11  -0.02672991  0.024764064 -0.020867732 -0.001379272 -0.37056959 -0.07738893
## 55   0.00000000  0.046854906  0.046591198  0.041495533 -0.33958818 -0.05795700
## 35  -0.04685491  0.000000000  0.002364665  0.007803869 -0.31486524 -0.11069078
## 15  -0.04659120 -0.002364665  0.000000000 -0.021931578 -0.37206429 -0.02449756
## 32  -0.04149553 -0.007803869  0.021931578  0.000000000 -0.32196823 -0.03384433
## 142  0.33958818  0.314865243  0.372064291  0.321968231  0.00000000  0.31528356
## 29   0.05795700  0.110690782  0.024497558  0.033844329 -0.31528356  0.00000000
## 30   0.01892367  0.086773138  0.044550745  0.045373405 -0.32553933 -0.00145666
## 60   0.06555164  0.010761038  0.112777350  0.009235843 -0.26533723  0.04293625
```

```
## 5    0.28250681  0.241241073  0.305518307  0.245223534  0.07649684  0.23549207
##               30           60            5
## 13  -0.097781500 -0.082449062 -0.28506364
## 21   0.346679671  0.336870589  0.04631932
## 130  0.375146337  0.258805300 -0.08286085
## 44  -0.020424371 -0.118828756 -0.30726580
## 18  -0.063443921 -0.074854175 -0.28529608
## 11  -0.103985696 -0.047837003 -0.27898923
## 55  -0.018923669 -0.065551641 -0.28250681
## 35  -0.086773138 -0.010761038 -0.24124107
## 15  -0.044550745 -0.112777350 -0.30551831
## 32  -0.045373405 -0.009235843 -0.24522353
## 142  0.325539328  0.265337232 -0.07649684
## 29   0.001456660 -0.042936247 -0.23549207
## 30   0.000000000  0.004873645 -0.23532162
## 60  -0.004873645  0.000000000 -0.36590306
## 5    0.235321617  0.365903064  0.00000000
```

```r
library(coda4microbiome)
library(microbiomeutilities)
set.seed(123)

ps <- readRDS("ps1.dna.genus_n0.rds")
ps <- format_to_besthit(ps)
abundance <- as.data.frame(otu_table(ps))
metadata <- sample_data(ps)

abundance <- abundance[-c(65,78),]
metadata <- metadata[-c(65,78),]

abundance <- as.matrix(abundance)

var_logratios<-
  explore_logratios(x=abundance, y=metadata$Segmented.neutrophils, measure = "glm")
```

5.1.1.21 Segmented Neutrophils Results

Name of the most important variables

```r
var_logratios$`name of most important variables`
```

```
##  [1] "ASV12:g__Dolosigranulum"          "ASV150:g__Parvimonas"
##  [3] "ASV1733:g__Lachnospiraceae_[G-7]" "ASV73:g__Oribacterium"
##  [5] "ASV27:g__Haemophilus"             "ASV136:g__Mesorhizobium"
##  [7] "ASV349:g__Dialister"              "ASV53:g__Stomatobaculum"
##  [9] "ASV176:g__Megasphaera"            "ASV1294:f__Sphingomonadaceae"
## [11] "ASV4:g__Corynebacterium"          "ASV1166:g__Veillonellaceae_[G-1]"
## [13] "ASV260:g__Sphingomonas"           "ASV1213:g__Olsenella"
## [15] "ASV9320:f__Aerococcaceae"
```

The pair of taxa whose log-ratio is more associated with the variable

```r
var_logratios$`max log-ratio`
```

```
## [1] "30" "14"
```

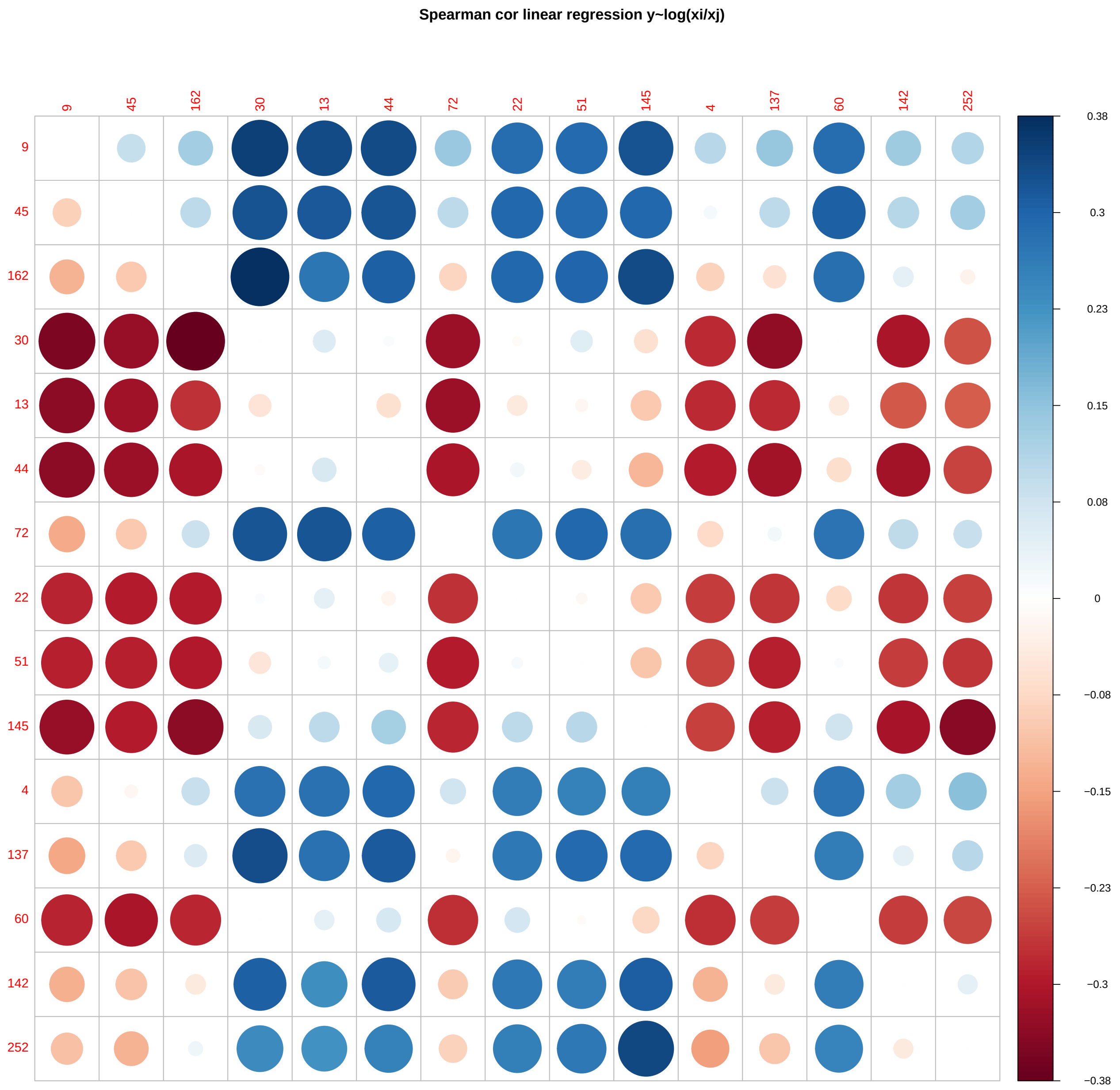

Figure 23: Correlation-like plot of the association of pairwise log-ratios with segmented neutrophils.

```
var_logratios$`names max log-ratio`
```

```
## [1] "ASV73:g__Oribacterium"       "ASV33:g__Peptostreptococcus"
```

The correlation value between the log-ratios and the variable

```
var_logratios$`association log-ratio with y`[1:15,1:15]
```

```
##               9          45         162           30          13          44
## 9    0.00000000  0.08772712  0.13167817  0.3551116947 0.33962238  0.34145975
## 45  -0.08772712  0.00000000  0.10023510  0.3300771780 0.31959094  0.32613910
## 162 -0.13167817 -0.10023510  0.00000000  0.3797536003 0.27667023  0.30844504
## 30  -0.35511169 -0.33007718 -0.37975360  0.0000000000 0.05588670  0.01130600
## 13  -0.33962238 -0.31959094 -0.27667023 -0.0558866955 0.00000000 -0.06325021
## 44  -0.34145975 -0.32613910 -0.30844504 -0.0113060003 0.06325021  0.00000000
## 72  -0.14349707 -0.10227266  0.08293787  0.3236440233 0.32515690  0.30808443
## 22  -0.29108547 -0.29712678 -0.29906625  0.0098996365 0.04521157 -0.02217313
## 51  -0.29312113 -0.29471771 -0.30369871 -0.0528280220 0.01754002  0.04085875
## 145 -0.32957967 -0.29653736 -0.34131791  0.0618599695 0.10118759  0.12873690
## 4   -0.10540275 -0.01880434  0.08597638  0.2829957826 0.28172441  0.29852215
## 137 -0.14595093 -0.10046062  0.05751731  0.3349287697 0.28318282  0.31622058
## 60  -0.29038616 -0.31122780 -0.28544345  0.0006151995 0.04348777  0.06588040
## 142 -0.13596920 -0.10787566 -0.04509968  0.3077154488 0.23423177  0.31802134
## 252 -0.11220934 -0.13100647  0.02331449  0.2394214082 0.22925939  0.25549749
##              72           22           51         145           4         137
## 9    0.14349707  0.291085469  0.293121133  0.32957967  0.10540275  0.14595093
## 45   0.10227266  0.297126780  0.294717707  0.29653736  0.01880434  0.10046062
## 162 -0.08293787  0.299066254  0.303698711  0.34131791 -0.08597638 -0.05751731
## 30  -0.32364402 -0.009899636  0.052828022 -0.06185997 -0.28299578 -0.33492877
## 13  -0.32515690 -0.045211566 -0.017540022 -0.10118759 -0.28172441 -0.28318282
## 44  -0.30808443  0.022173135 -0.040858746 -0.12873690 -0.29852215 -0.31622058
## 72   0.00000000  0.274065581  0.298403997  0.28615044 -0.07271509  0.02119056
## 22  -0.27406558  0.000000000 -0.013729070 -0.10184184 -0.26568876 -0.27025151
## 51  -0.29840400  0.013729070  0.000000000 -0.10375878 -0.25477960 -0.29512783
## 145 -0.28615044  0.101841839  0.103758782  0.00000000 -0.26169368 -0.29247443
## 4    0.07271509  0.265688761  0.254779595  0.26169368  0.00000000  0.08006945
## 137 -0.02119056  0.270251507  0.295127831  0.29247443 -0.08006945  0.00000000
## 60  -0.27822774  0.069930264 -0.008093734 -0.07851966 -0.28078498 -0.26322008
## 142 -0.09651701  0.272057857  0.265378306  0.31192492 -0.13197900 -0.04500210
## 252 -0.08720468  0.259864237  0.269714001  0.34470313 -0.15706405 -0.10337224
##                 60         142         252
## 9    0.2903861597  0.13596920  0.11220934
## 45   0.3112278004  0.10787566  0.13100647
## 162  0.2854434542  0.04509968 -0.02331449
## 30  -0.0006151995 -0.30771545 -0.23942141
## 13  -0.0434877712 -0.23423177 -0.22925939
## 44  -0.0658804007 -0.31802134 -0.25549749
## 72   0.2782277394  0.09651701  0.08720468
## 22  -0.0699302635 -0.27205786 -0.25986424
## 51   0.0080937342 -0.26537831 -0.26971400
## 145  0.0785196614 -0.31192492 -0.34470313
## 4    0.2807849785  0.13197900  0.15706405
## 137  0.2632200814  0.04500210  0.10337224
## 60   0.0000000000 -0.26245664 -0.25215194
## 142  0.2624566371  0.00000000  0.04247775
```

```
## 252  0.2521519366 -0.04247775  0.00000000
```

```r
library(coda4microbiome)
library(microbiomeutilities)
set.seed(123)

ps <- readRDS("ps1.dna.genus_n0.rds")
ps <- format_to_besthit(ps)
abundance <- as.data.frame(otu_table(ps))
metadata <- sample_data(ps)

abundance <- abundance[-c(66,78),]
metadata <- metadata[-c(66,78),]

abundance <- as.matrix(abundance)

var_logratios<-
  explore_logratios(x=abundance, y=metadata$Serum.creatinine, measure = "glm")
```

5.1.1.22 Serum creatinine Results

Name of the most important variables

```r
var_logratios$`name of most important variables`
```

```
##  [1] "ASV22:g__Leptotrichia"
##  [2] "ASV293:g__Mogibacterium"
##  [3] "ASV349:g__Dialister"
##  [4] "ASV57:g__Capnocytophaga"
##  [5] "ASV516:g__Tannerella"
##  [6] "ASV660:f__Streptococcaceae"
##  [7] "ASV8:g__Rothia"
##  [8] "ASV40:g__Acinetobacter"
##  [9] "ASV33:g__Peptostreptococcus"
## [10] "ASV876:c__Bacteroidetes"
## [11] "ASV527:c__Actinobacteria"
## [12] "ASV810:g__Peptostreptococcaceae_[XI][G-7]"
## [13] "ASV64:g__Porphyromonas"
## [14] "ASV51:g__Pseudomonas"
## [15] "ASV92:g__Saccharibacteria_(TM7)_[G-1]"
```

The pair of taxa whose log-ratio is more associated with the variable

```r
var_logratios$`max log-ratio`
```

```
## [1] "73" "45"
```

```r
var_logratios$`names max log-ratio`
```

```
## [1] "ASV359:g__Aggregatibacter" "ASV150:g__Parvimonas"
```

The correlation value between the log-ratios and the variable

```r
var_logratios$`association log-ratio with y`[1:15,1:15]
```

```
##               11          64          72          24          91         103
## 11   0.000000000  0.17665076  0.113423490  0.03732823  0.113245731  0.189007926
```

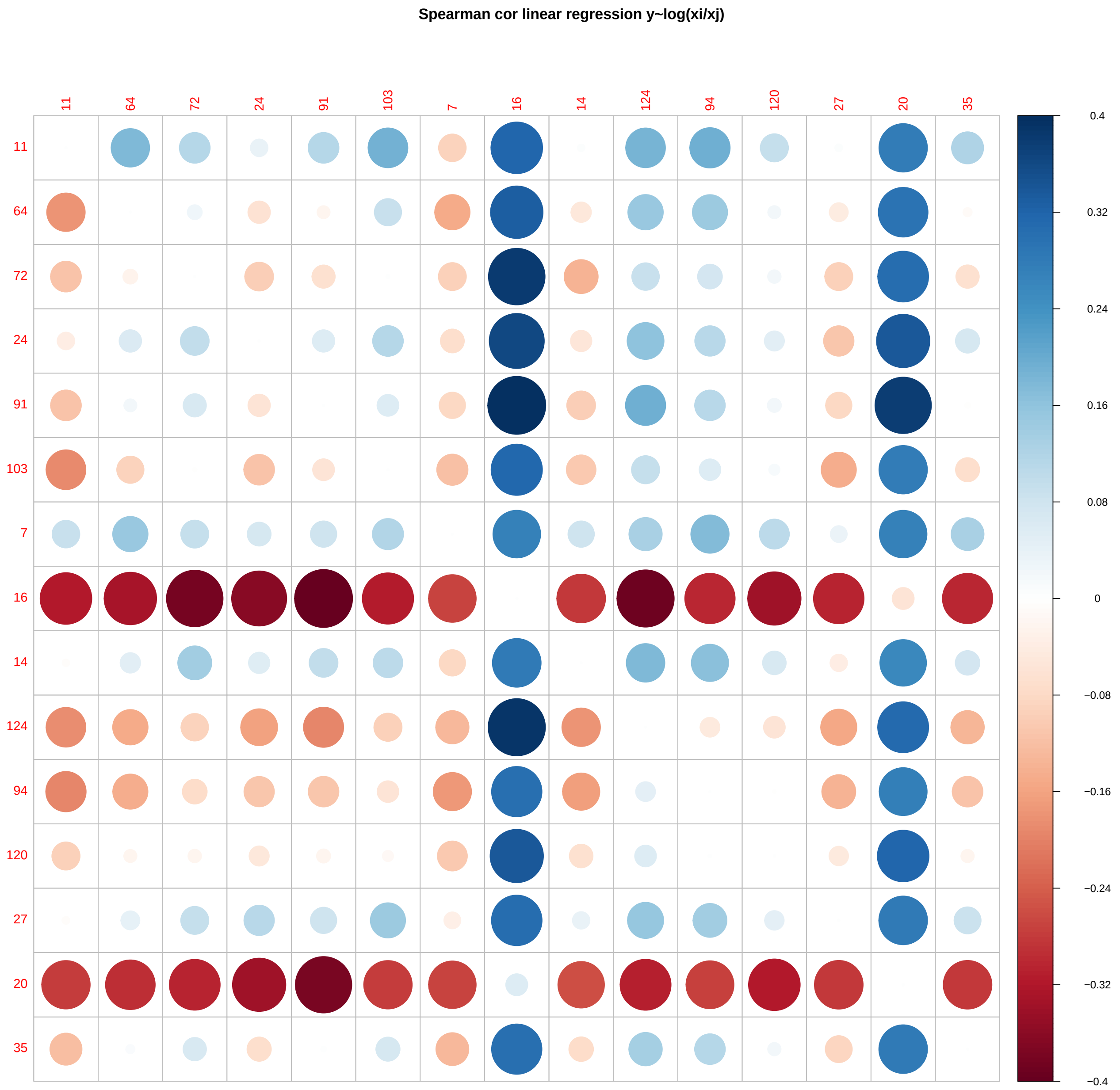

Figure 24: Correlation-like plot of the association of pairwise log-ratios with serum creatinine.

```
## 64  -0.176650761  0.00000000  0.026699248 -0.06043719 -0.020289927  0.088759968
## 72  -0.113423490 -0.02669925  0.000000000 -0.09935170 -0.064666683  0.002032953
## 24  -0.037328229  0.06043719  0.099351698  0.00000000  0.060013675  0.113003467
## 91  -0.113245731  0.02028993  0.064666683 -0.06001368  0.000000000  0.057182148
## 103 -0.189007926 -0.08875997 -0.002032953 -0.11300347 -0.057182148  0.000000000
## 7    0.091472796  0.14897414  0.093607073  0.06821538  0.083133363  0.116516604
## 16  -0.318167216 -0.32875261 -0.381763729 -0.36133046 -0.400862924 -0.313586209
## 14  -0.006864206  0.05005133  0.137840234  0.05467350  0.099273630  0.105138975
## 124 -0.188374687 -0.14958595 -0.090826379 -0.16254487 -0.192512904 -0.094676741
## 94  -0.194243525 -0.14757405 -0.073562291 -0.11041301 -0.112151194 -0.056186920
## 120 -0.094765991 -0.02058712 -0.021803302 -0.04849488 -0.023805413 -0.014991115
## 27  -0.007322076  0.04302281  0.094019491  0.11070088  0.082794273  0.147687490
## 20  -0.280161073 -0.29443575 -0.308176664 -0.33909198 -0.379444726 -0.280135642
## 35  -0.122860035  0.01014339  0.065472194 -0.07053338  0.003785609  0.069765389
##               7          16            14         124           94          120
## 11  -0.09147280 0.31816722  0.006864206  0.18837469  0.194243525  0.094765991
## 64  -0.14897414 0.32875261 -0.050051328  0.14958595  0.147574050  0.020587119
## 72  -0.09360707 0.38176373 -0.137840234  0.09082638  0.073562291  0.021803302
## 24  -0.06821538 0.36133046 -0.054673504  0.16254487  0.110413008  0.048494883
## 91  -0.08313336 0.40086292 -0.099273630  0.19251290  0.112151194  0.023805413
## 103 -0.11651660 0.31358621 -0.105138975  0.09467674  0.056186920  0.014991115
## 7    0.00000000 0.27109583  0.083200592  0.13215106  0.174716795  0.107547687
## 16  -0.27109583 0.00000000 -0.282897620 -0.39079289 -0.302563756 -0.339192376
## 14  -0.08320059 0.28289762  0.000000000  0.17723795  0.165694674  0.067018234
## 124 -0.13215106 0.39079289 -0.177237952  0.00000000 -0.047164393 -0.056686452
## 94  -0.17471680 0.30256376 -0.165694674  0.04716439  0.000000000 -0.001395005
## 120 -0.10754769 0.33919238 -0.067018234  0.05668645  0.001395005  0.000000000
## 27  -0.03423735 0.30487930  0.036577026  0.15602396  0.137433599  0.045719991
## 20  -0.27005668 0.05775253 -0.258600420 -0.30967650 -0.274292242 -0.318056886
## 35  -0.12927640 0.30197387 -0.072352359  0.13352673  0.112894292  0.021272691
##              27          20           35
## 11   0.007322076  0.28016107  0.122860035
## 64  -0.043022811  0.29443575 -0.010143395
## 72  -0.094019491  0.30817666 -0.065472194
## 24  -0.110700880  0.33909198  0.070533376
## 91  -0.082794273  0.37944473 -0.003785609
## 103 -0.147687490  0.28013564 -0.069765389
## 7    0.034237351  0.27005668  0.129276398
## 16  -0.304879304 -0.05775253 -0.301973870
## 14  -0.036577026  0.25860042  0.072352359
## 124 -0.156023958  0.30967650 -0.133526728
## 94  -0.137433599  0.27429224 -0.112894292
## 120 -0.045719991  0.31805689 -0.021272691
## 27   0.000000000  0.28289569  0.087206241
## 20  -0.282895686  0.00000000 -0.282487425
## 35  -0.087206241  0.28248743  0.000000000
```

```
library(coda4microbiome)
library(microbiomeutilities)
set.seed(123)

ps <- readRDS("ps1.dna.genus_n0.rds")
ps <- format_to_besthit(ps)
```

```r
abundance <- as.data.frame(otu_table(ps))
metadata <- sample_data(ps)

abundance <- abundance[-c(66,78),]
metadata <- metadata[-c(66,78),]

abundance <- as.matrix(abundance)

var_logratios<-explore_logratios(x=abundance, y=metadata$Urea, measure = "glm")
```

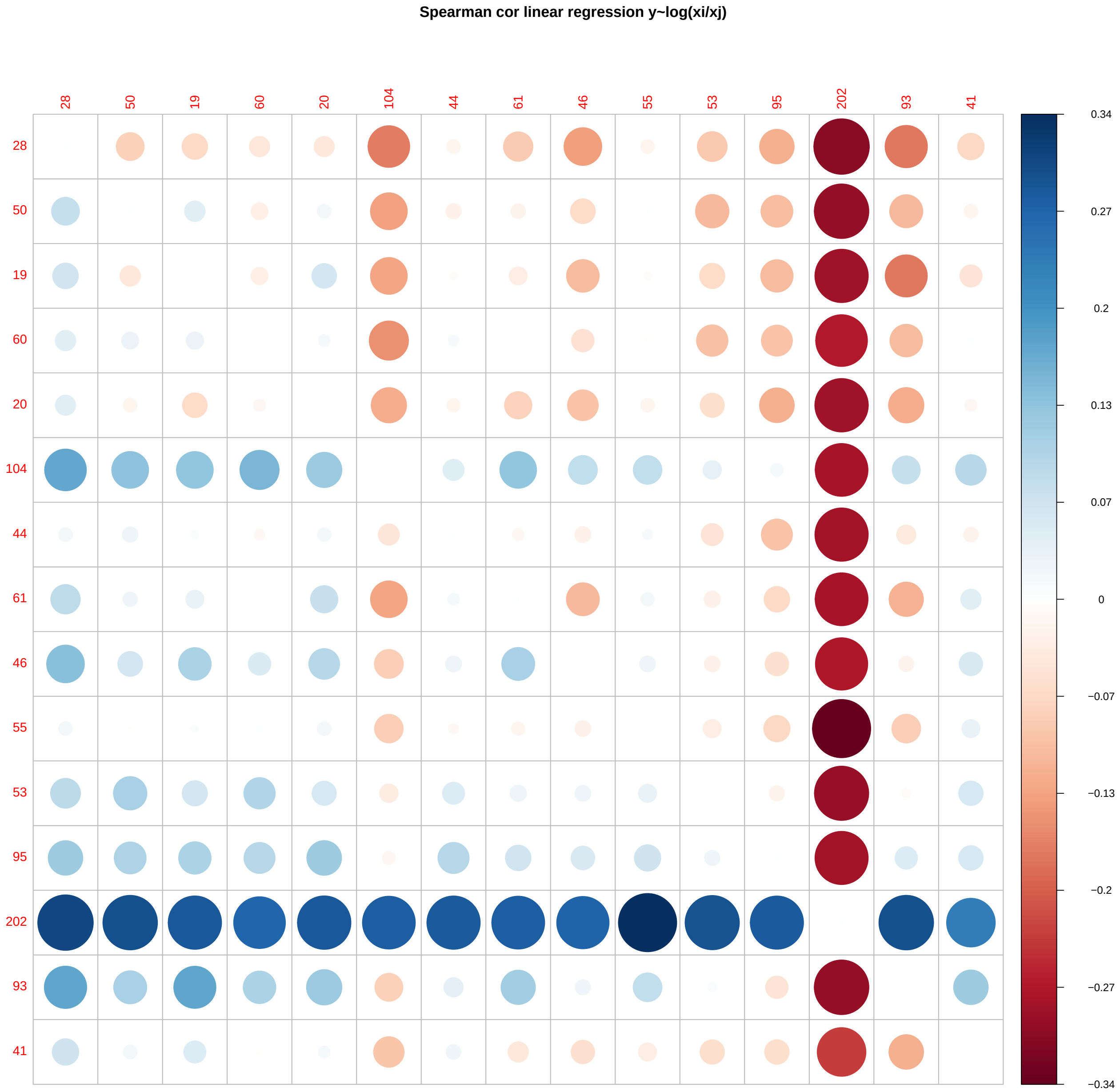

Figure 25: Correlation-like plot of the association of pairwise log-ratios with serum urea.

5.1.1.23 Urea Results

Name of the most important variables

```r
var_logratios$`name of most important variables`
```

```
##  [1] "ASV68:f__Comamonadaceae"
##  [2] "ASV170:g__Ralstonia"
##  [3] "ASV45:g__Leptothrix"
##  [4] "ASV260:g__Sphingomonas"
##  [5] "ASV51:g__Pseudomonas"
##  [6] "ASV664:g__Flavitalea"
##  [7] "ASV136:g__Mesorhizobium"
##  [8] "ASV261:g__Brevundimonas"
##  [9] "ASV152:g__Delftia"
## [10] "ASV212:g__Saccharibacteria_(TM7)_[G-3]"
## [11] "ASV203:g__Mycobacterium"
## [12] "ASV543:g__Afipia"
## [13] "ASV2953:g__Turicella"
## [14] "ASV524:g__Caulobacter"
## [15] "ASV116:g__Acidovorax"
```

The pair of taxa whose log-ratio is more associated with the variable

```r
var_logratios$`max log-ratio`
```

```
## [1] "176" "255"
```

```r
var_logratios$`names max log-ratio`
```

```
## [1] "ASV2099:f__Ruminococcaceae"    "ASV11251:f__Carnobacteriaceae"
```

The correlation value between the log-ratios and the variable

```r
var_logratios$`association log-ratio with y`[1:15,1:15]
```

```
##             28           50           19            60          20         104
## 28  0.00000000 -0.077199970 -0.065210111 -0.0413458448 -0.04066570 -0.17205228
## 50  0.07719997  0.000000000  0.041868169 -0.0286105610  0.01912619 -0.13439030
## 19  0.06521011 -0.041868169  0.000000000 -0.0298936304  0.06069478 -0.13399913
## 60  0.04134584  0.028610561  0.029893630  0.0000000000  0.01423403 -0.15199283
## 20  0.04066570 -0.019126189 -0.060694778 -0.0142340253  0.00000000 -0.12329762
## 104 0.17205228  0.134390296  0.133999126  0.1519928288  0.12329762  0.00000000
## 44  0.01889271  0.023833024  0.006456865 -0.0115017525  0.01808494 -0.04474578
## 61  0.08602710  0.021342424  0.032447374  0.0004794489  0.07414832 -0.13329917
## 46  0.14081201  0.061210673  0.105546864  0.0504506232  0.09392444 -0.08306383
## 55  0.01876556 -0.001013039  0.006114786  0.0023438971  0.01887038 -0.08124706
## 53  0.08844278  0.110557724  0.063170790  0.0978749211  0.05811496 -0.03396516
## 95  0.11824831  0.101073902  0.103949771  0.0954560506  0.11922728 -0.01668130
## 202 0.30447069  0.294208868  0.281749333  0.2649794409  0.28259504  0.27598078
## 93  0.17731428  0.108295186  0.175544701  0.1051321934  0.12365923 -0.07769700
## 41  0.06969135  0.018989253  0.048882422 -0.0032649989  0.01385745 -0.09263688
##               44            61          46           55           53
## 28  -0.018892706 -0.0860270977 -0.14081201 -0.018765565 -0.088442776
## 50  -0.023833024 -0.0213424239 -0.06121067  0.001013039 -0.110557724
## 19  -0.006456865 -0.0324473736 -0.10554686 -0.006114786 -0.063170790
## 60   0.011501753 -0.0004794489 -0.05045062 -0.002343897 -0.097874921
## 20  -0.018084941 -0.0741483154 -0.09392444 -0.018870379 -0.058114964
## 104  0.044745781  0.1332991719  0.08306383  0.081247059  0.033965162
## 44   0.000000000 -0.0139450547 -0.02561576  0.010354875 -0.048659152
## 61   0.013945055  0.0000000000 -0.10764571  0.017425990 -0.026799412
```

```
## 46   0.025615764  0.1076457131  0.00000000  0.025087791 -0.025023785
## 55  -0.010354875 -0.0174259896 -0.02508779  0.000000000 -0.032967499
## 53   0.048659152  0.0267994122  0.02502379  0.032967499  0.000000000
## 95   0.096224874  0.0650997960  0.05579670  0.068916714  0.023396938
## 202  0.281115669  0.2767287990  0.27091494  0.335280575  0.291124609
## 93   0.036949625  0.1167808654  0.02305644  0.082108879  0.007792007
## 41   0.021693029 -0.0411746202 -0.05464627 -0.032580511 -0.059400183
##              95        202           93           41
## 28  -0.11824831 -0.3044707 -0.177314283 -0.069691351
## 50  -0.10107390 -0.2942089 -0.108295186 -0.018989253
## 19  -0.10394977 -0.2817493 -0.175544701 -0.048882422
## 60  -0.09545605 -0.2649794 -0.105132193  0.003264999
## 20  -0.11922728 -0.2825950 -0.123659234 -0.013857447
## 104  0.01668130 -0.2759808  0.077696996  0.092636885
## 44  -0.09622487 -0.2811157 -0.036949625 -0.021693029
## 61  -0.06509980 -0.2767288 -0.116780865  0.041174620
## 46  -0.05579670 -0.2709149 -0.023056437  0.054646270
## 55  -0.06891671 -0.3352806 -0.082108879  0.032580511
## 53  -0.02339694 -0.2911246 -0.007792007  0.059400183
## 95   0.00000000 -0.2798373  0.050192919  0.059743370
## 202  0.27983732  0.0000000  0.294978735  0.234415315
## 93  -0.05019292 -0.2949787  0.000000000  0.118631658
## 41  -0.05974337 -0.2344153 -0.118631658  0.000000000
```

6.0 Session info

```
sessionInfo()
```

```
## R version 4.4.2 (2024-10-31)
## Platform: x86_64-pc-linux-gnu
## Running under: Ubuntu 22.04.5 LTS
##
## Matrix products: default
## BLAS:   /usr/lib/x86_64-linux-gnu/blas/libblas.so.3.10.0
## LAPACK: /usr/lib/x86_64-linux-gnu/lapack/liblapack.so.3.10.0
##
## locale:
##  [1] LC_CTYPE=pt_BR.UTF-8       LC_NUMERIC=C
##  [3] LC_TIME=pt_BR.UTF-8        LC_COLLATE=pt_BR.UTF-8
##  [5] LC_MONETARY=pt_BR.UTF-8    LC_MESSAGES=pt_BR.UTF-8
##  [7] LC_PAPER=pt_BR.UTF-8       LC_NAME=C
##  [9] LC_ADDRESS=C               LC_TELEPHONE=C
## [11] LC_MEASUREMENT=pt_BR.UTF-8 LC_IDENTIFICATION=C
##
## time zone: America/Sao_Paulo
## tzcode source: system (glibc)
##
## attached base packages:
## [1] stats     graphics  grDevices utils     datasets  methods   base
##
## other attached packages:
##  [1] microbiomeutilities_1.00.17 microbiome_1.26.0
##  [3] coda4microbiome_0.2.4       DT_0.33
```

```
##  [5] ecodist_2.1.3            easyCODA_0.40.2
##  [7] zCompositions_1.5.0-4    truncnorm_1.0-9
##  [9] NADA_1.6-1.1             survival_3.8-3
## [11] MASS_7.3-64              ggrepel_0.9.6
## [13] vegan_2.6-8              lattice_0.22-5
## [15] permute_0.9-7            rio_1.2.3
## [17] lubridate_1.9.3          forcats_1.0.0
## [19] stringr_1.5.1            dplyr_1.1.4
## [21] purrr_1.0.2              readr_2.1.5
## [23] tidyr_1.3.1              tibble_3.2.1
## [25] tidyverse_2.0.0          ggpubr_0.6.0
## [27] ggplot2_3.5.1            phyloseq_1.48.0
##
## loaded via a namespace (and not attached):
##   [1] RColorBrewer_1.1-3      rstudioapi_0.15.0       jsonlite_1.8.8
##   [4] shape_1.4.6.1           magrittr_2.0.3          corrplot_0.95
##   [7] farver_2.1.2            rmarkdown_2.29          GlobalOptions_0.1.2
##  [10] zlibbioc_1.50.0         vctrs_0.6.5             multtest_2.60.0
##  [13] rstatix_0.7.2           tinytex_0.54            htmltools_0.5.8.1
##  [16] broom_1.0.7             Rhdf5lib_1.26.0         pROC_1.18.5
##  [19] Formula_1.2-5           rhdf5_2.48.0            htmlwidgets_1.6.4
##  [22] plyr_1.8.9              zoo_1.8-12              igraph_2.1.3
##  [25] lifecycle_1.0.4         iterators_1.0.14        pkgconfig_2.0.3
##  [28] Matrix_1.7-1            R6_2.5.1                fastmap_1.2.0
##  [31] GenomeInfoDbData_1.2.12 clue_0.3-66             digest_0.6.37
##  [34] colorspace_2.1-1        S4Vectors_0.42.1        ellipse_0.5.0
##  [37] labeling_0.4.3          km.ci_0.5-6             timechange_0.3.0
##  [40] httr_1.4.7              abind_1.4-8             mgcv_1.9-1
##  [43] compiler_4.4.2          withr_3.0.2             doParallel_1.0.17
##  [46] backports_1.5.0         carData_3.0-5           R.utils_2.12.3
##  [49] ggsignif_0.6.4          rjson_0.2.23            biomformat_1.32.0
##  [52] tools_4.4.2             ape_5.8-1               R.oo_1.27.0
##  [55] glue_1.8.0              nlme_3.1-166            rhdf5filters_1.16.0
##  [58] grid_4.4.2              Rtsne_0.17              cluster_2.1.8
##  [61] reshape2_1.4.4          ade4_1.7-22             generics_0.1.3
##  [64] gtable_0.3.6            KMsurv_0.1-5            tzdb_0.4.0
##  [67] R.methodsS3_1.8.2       ca_0.71.1               survminer_0.5.0
##  [70] data.table_1.15.0       hms_1.1.3               car_3.1-3
##  [73] XVector_0.44.0          BiocGenerics_0.50.0     foreach_1.5.2
##  [76] pillar_1.10.1           circlize_0.4.16         splines_4.4.2
##  [79] gghalves_0.1.4          tidyselect_1.2.1        ComplexHeatmap_2.20.0
##  [82] Biostrings_2.72.1       knitr_1.49              gridExtra_2.3
##  [85] IRanges_2.38.1          stats4_4.4.2            xfun_0.50
##  [88] Biobase_2.64.0          matrixStats_1.5.0       pheatmap_1.0.12
##  [91] stringi_1.8.4           UCSC.utils_1.0.0        yaml_2.3.8
##  [94] evaluate_1.0.3          codetools_0.2-19        cli_3.6.3
##  [97] xtable_1.8-4            munsell_0.5.1           survMisc_0.5.6
## [100] Rcpp_1.0.14             GenomeInfoDb_1.40.1     png_0.1-8
## [103] parallel_4.4.2          glmnet_4.1-8            scales_1.3.0
## [106] crayon_1.5.3            writexl_1.5.1           GetoptLong_1.0.5
## [109] rlang_1.1.5             formatR_1.14
```
