# Supplementary material for: Unveiling the role of the upper respiratory tract microbiome in susceptibility and severity to COVID-19
Source: Front Cell Infect Microbiol. 2025 May 13;15:1531084. doi: 10.3389/fcimb.2025.1531084 (PMC12106449; doi:10.3389/fcimb.2025.1531084)
Supplement: Supplementary file 5 [file Table2.docx]

Supplementary Table 1. Differentially abundant taxa between experimental groups and their association with clinical variables. *A positive association means the biomarker value directly correlates with the taxon abundance. **A negative association implies that the biomarker value is inverse correlated with the taxon abundance. ***Absolute counts. NA, No Association.

| Taxa | Positive association* | Negative association** |
| --- | --- | --- |
| *Abiotrophia* sp. | eosinophils (%) | LDH |
| *Actinomyces* sp. | leukocytes, rod neutrophils*** | MCH, MCV |
| *Alloprevotella* sp. | age, ALT, AST | NA |
| *Bifidobacterium* sp. | lymphocytes (%) | age |
| *Campylobacter* sp. | AST | NA |
| *Catonella* sp. | NA | CPK, lymphocytes (%) |
| *Dialister* sp. | NA | leukocytes, monocytes***, segmented neutrophils*** |
| *Dolosigranulum* sp. | CPK | age, d-dimer, leukocytes, segmented neutrophils*** |
| *Enterococcus* sp. | lymphocytes (%), MCH, MCHC, MCV, monocytes (%), monocytes*** | d-dimer, RDW |
| *Fusobacterium* sp. | LDH | eosinophils (%), monocytes (%) |
| *Gemella* sp. | CRP, LDH, rod neutrophils*** | eosinophils (%), lymphocytes (%), monocytes (%) |
| *Granulicatella* sp. | age | NA |
| *Haemophilus* sp. | CRP, LDH, rod neutrophils***, segmented neutrophils*** | eosinophils (%), erythrocytes, hematocrit, hemoglobin, lymphocytes (%), monocytes (%) |
| *Lachnoanaerobaculum* sp. | age | MCH, MCV |
| *Lactobacillus* sp. | NA | CRP, rod neutrophils*** |
| *Leptotrichia* sp. | rod neutrophils*** | CPK, serum creatinine |
| *Mesorhizobium* sp. | d-dimer, LDH, RDW, rod neutrophils***, segmented neutrophils*** | lymphocytes (%), monocytes (%) |
| *Neisseria* sp. | CRP | NA |
| *Novosphingobium* sp. | CRP, RDW, rod neutrophils*** | ALT, erythrocytes, hematocrit, hemoglobin, MCHC |
| *Oribacterium* sp. | d-dimer, leukocytes, rod neutrophils***, segmented neutrophils*** | ALT, CPK, lymphocytes (%) |
| *Saccharibacteria*_(TM7)_[G-1] | rod neutrophils*** | CPK, erythrocytes |
| *Saccharibacteria*_(TM7)_[G-3] | LDH, rod neutrophils*** | CPK, erythrocytes, lymphocytes (%), monocytes (%), monocytes*** |
| *Staphylococcus* sp. | monocytes (%) | LDH, rod neutrophils*** |
| *Treponema* sp. | NA | Monocytes*** |
| *Veillonella* sp. | d-dimer | MCH, MCHC |
